# Misremembering Solitude: The Role of Personality and Cultural Self‐Concepts in Shaping Discrepancies Between Recalled and Concurrent Affect in Solitude

**DOI:** 10.1111/jopy.12971

**Published:** 2024-08-16

**Authors:** Jennifer C. Lay, Yuen Wan Ho, Dwight C. K. Tse, Jimmy T. K. Tse, Da Jiang

**Affiliations:** ^1^ Department of Psychology University of Exeter Exeter UK; ^2^ Department of Psychology Lingnan University Hong Kong SAR China; ^3^ Department of Psychological Sciences and Health University of Strathclyde Glasgow UK; ^4^ Centre for Psychiatry Wolfson Institute of Preventive Medicine London UK; ^5^ Barts the London School of Medicine and Dentistry Queen Mary University of London London UK; ^6^ Department of Special Education and Counselling The Education University of Hong Kong Hong Kong SAR China

**Keywords:** affect, culture, experience sampling method, memory, retrospective reports, self‐concept, solitude

## Abstract

**Background:**

Affect recall is key to psychological assessment and decision‐making. However, self‐concepts (self‐beliefs) may bias retrospective affect reports such that they deviate from lived experiences. Does this experience‐memory gap apply to solitude experiences? We hypothesized that individuals misremember how they feel overall and when in solitude, in line with self‐concepts of introversion, self‐determined/not‐self‐determined solitude motivations, and independent/interdependent self‐construal. A pilot study comparing retrospective to daily affect reports captured over 2 weeks (*N* = 104 UK university students) provided preliminary evidence of introversion and not‐self‐determined solitude shaping affect recall.

**Methods:**

In the main pre‐registered study, participants aged 18–49 in the UK (*N* = 160) and Hong Kong (*N* = 159) reported their momentary affective states and social situations 5 times per day over 7 days, then recalled how they felt over the week.

**Results and Discussion:**

Individuals higher in self‐determined solitude were more prone to retrospectively overestimate their high‐ and low‐arousal positive affect in solitude and showed less overestimation/more underestimation of negative affect in solitude. Higher not‐self‐determined solitude was associated with overestimating loneliness, and higher interdependent self‐construal with overestimating loneliness and energy levels, in solitude. Comparisons based on residence/ethnicity suggest culture influences solitude‐seeking and affective memory. Implications for well‐being and affect measurement are discussed.

## 1 Introduction

Recalling our past feelings is key to guiding our daily life behavior—for example, balancing solitary and social activities to maximize well‐being (Levine, Lench, and Safer 2009). However, this affect recall is not always accurate. Previous research suggests that self‐concepts (beliefs about ourselves, including self‐reported personality traits) bias our affect recall such that recalled experiences align more with expectations than with actual lived experiences (Lay et al. 2017; Robinson and Clore 2002). It is unclear, however, to what extent self‐concepts shape our recall of affective experiences in specific social situations. This study investigates whether individuals misremember how they feel in solitude (time without social contact). Solitude is a ubiquitous experience with significant well‐being impacts (Long and Averill 2003; Lay et al. 2017, 2019; Nguyen, Weinstein, and Ryan 2021). We examine how self‐concepts related to solitude experiences (introversion, self‐determined and not‐self‐determined solitude motivation) and to culture (independent and interdependent self‐construal) shape affect recall among individuals in the United Kingdom (UK) and Hong Kong (HK). This Stage 2 Registered Report builds on the Stage 1 study design pre‐registered on the Open Science Framework (OSF) at: https://osf.io/v4yca.

Affect self‐reports capture unique and useful information about subjective experiences that have important well‐being implications. For example, feelings of loneliness (stemming from the perception that one's social needs are not being met) have significant impacts on subsequent behavior and long‐term health, beyond the effects of objective social isolation (Hawkley and Cacioppo 2010). To capture affective experiences, many researchers adopt ecological momentary assessments or experience sampling methods (ESM; e.g., Larson 1990), as these methods capture fleeting experiences while minimizing the influence of memory retrieval processes (Schwarz 2007). However, global assessments of affective experiences over a given period (*retrospective reports*) are often needed. For example, clinical measures of anxiety may involve reporting affective symptoms over the preceding 2 weeks. How accurate are such retrospective affect measures?

Previous research has shown that individuals systematically exaggerate certain affective states when recalling how they had felt over a given period (Barrett 1997; Robinson and Clore 2002). If we compare *retrospective reports* of overall affect (e.g., “How happy have you felt, on average, over the past two weeks?”) with *concurrent reports* obtained via ESM (e.g., “How happy do you feel right now?”, asked repeatedly over several days), individuals across the adult lifespan have been shown to retrospectively report higher levels of positive and/or negative affect than their average concurrent ratings (e.g., Colombo et al. 2020; Lay et al. 2017; Neubauer et al. 2020). Affect circumplex models (e.g., Russell 1980; Tsai, Knutson, and Fung 2006) define affect in terms of two underlying dimensions: valence (positive vs. negative) and activation (high vs. low arousal). Previous research suggests that individuals may specifically exaggerate high‐arousal positive and negative affective states (e.g., “excited” and “anxious”) in retrospective reports, although findings are mixed regarding retrospective reporting of low‐arousal states (e.g., “calm” and “sleepy”; Lay et al. 2017; Mill, Realo, and Allik 2016).

### 1.1 Sources of Affect Recall Bias

How do biases emerge in retrospective affect reports? When recalling affective experiences over a relatively recent period (of several hours or days), reports tend to be biassed in the direction of peak (high‐intensity) and recent experiences (Fredrickson 2000; Kahneman 2000). Recall in favor of such emotionally salient experiences may serve important functions for goal pursuit and coping with challenges (Levine, Lench, and Safer 2009).

Peak and recency effects fade over time, as memories of the affective experiences fade (Kahneman 2000; Robinson and Clore 2002). When recalling affect over longer periods (several weeks or more), individuals rely increasingly on abstract knowledge and heuristics to fill gaps in memory. These may include situation‐specific beliefs (e.g., “summer is a happy time of year”), and identity‐related beliefs or *self‐concepts* (e.g., “I am a happy person”; Robinson and Clore 2002). A self‐concept may be defined as a stable set of beliefs or knowledge about oneself (e.g., self‐schema; Markus 1977) which links concepts of self with concepts describing personality (Asendorpf, Banse, and Mücke 2002). For example, someone with a self‐concept of being extraverted (reflected by a high score on a self‐report measure of extraversion) may have a greater tendency to exaggerate their positive affect and underreport their negative affect in retrospective as compared to concurrent reports (Barrett 1997; Lay et al. 2017; Mill, Realo, and Allik 2016). Retrospective report biases may also be arousal‐specific; for example, extraversion has been specifically linked with overreporting high‐arousal positive affective states (e.g., “happy”; Lay et al. 2017; Mill, Realo, and Allik 2016) and with underreporting low‐arousal positive states (e.g., “calm”; Lay et al. 2017). These recall biases align with key features of extraversion (positive affectivity, high energy) captured in personality inventories (e.g., Big Five Inventory; Costa and McCrae 1980). In general, self‐concepts fill gaps in memory in ways that maintain a coherent sense of self (Conway and Pleydell‐Pearce 2000).

Most previous research linking self‐concept with affect recall biases has focused on recalling overall affect across situations (e.g., Barrett 1997; Lay et al. 2017; Oishi 2002). However, self‐concept may also shape situation‐specific affective beliefs. For example, previous research has shown that individuals who report being more introverted (less extraverted) tend to underestimate how much they will enjoy a social situation when made to act extraverted (Zelenski et al. 2013). It is unclear, however, whether such affect recall biases extend to situations of solitude, which are just as ubiquitous in daily life as social situations, yet are understudied. The proposed study focuses on self‐concepts as a key information source shaping how individuals recall feeling in solitude.

### 1.2 Recalling Affect in Solitude

Working‐age adults spend an estimated 30%–40% of their time in solitude (e.g., Larson 1990; Papp et al. 2013), which is conceptualized as “the absence of social interaction” (Burger 1995) or “communicative separation from others” (Larson 1990). Much research treats physical aloneness (no one else present) as a precondition for solitude, defining solitude as a complete absence of social stimuli (e.g., Hatano et al. 2022; Nguyen, Ryan, and Deci 2018; Pauly et al. 2017). Other research treats solitude and physical aloneness separately, emphasizing how one can be with others but not interacting (e.g., Epley and Schroeder 2014; Lay et al. 2019; Long and Averill 2003; Ratner and Hamilton 2015). We define solitude here as the absence of in‐person or virtual social interaction, regardless of whether others are physically present. This definition accounts for the ways in which modern digital communication disrupts solitude (Campbell and Ross 2021) and also for the possibility that one may experience “solitude alone” and “solitude with others” differently (with the latter providing a form of ambient sociality or feeling of connection; McGonigal 2011; Weinstein, Hansen, and Nguyen 2023).

Previous ESM and daily survey studies suggest that spending time alone or in solitude (versus with others or interacting) has a deactivating effect on affect, with an overall pattern of findings suggesting reduced high‐arousal positive and negative affect, and increased low‐arousal positive and negative affect (e.g., Nguyen, Ryan, and Deci 2018; Pauly et al. 2017). Hence, solitude can bring both risks (e.g., loneliness) and benefits (e.g., calm), depending in large part on whether this solitude happens by choice (Lay et al. 2019; Long and Averill 2003; Nguyen, Ryan, and Deci 2018). Previous research suggests that health and longevity may be maximized by striking an optimal balance between solitary and social time (Stavrova and Ren 2021). Individuals who can accurately remember how solitude feels for them may be better able to achieve a balance that enhances their overall health and well‐being.

Although no previous research has compared retrospective to concurrent reports of affect while in solitude, *affect forecasting* studies offer insight into how people expect to feel. In experiments with US community samples, individuals tended to overestimate how much they would enjoy keeping to themselves in public places (versus talking to a stranger; Epley and Schroeder 2014). In contrast, a quasi‐experimental study of US university students showed those who were by themselves (rather than with a companion) were more prone to underestimate how much they would enjoy an art gallery (Ratner and Hamilton 2015). Moreover, Japanese and UK university students in a set of experiments underestimated how intrinsically motivated they would feel while waiting in a quiet room alone (Hatano et al. 2022). Hence, findings are mixed but suggest overall that individuals underestimate how good solitude will feel.

How might self‐concept shape recall of solitary experiences? Introversion is a key personality self‐concept related to social motivation. For example, affect forecasting studies suggest that individuals with high introversion scores may underestimate the pleasures of socializing, and even expect it will make them feel worse, even though most individuals in fact report feeling better after socializing (Duffy et al. 2018; Zelenski et al. 2013). In these studies, however, introversion did *not* consistently predict how individuals expected to feel when acting introverted or when keeping to themselves in public (Epley and Schroeder 2014; Zelenski et al. 2013). This may be because introversion captures a range of reasons for being in solitude, including genuine enjoyment of quiet and contemplation, but also shyness and low sociability (Zelenski, Sobocko, and Whelan 2021). These different motivations may have opposing effects on how introverts recall feeling in solitude. For example, those who enjoy solitude for its own sake may remember their solitude experiences positively, whereas those who seek solitude due to shyness may recall solitude experiences tinged with anxiety or regret over lost social opportunities.

To better understand how self‐concepts shape recall of solitude experiences, we need to examine self‐concepts that capture more specific reasons for being in solitude. Drawing on self‐determination theory (Ryan and Deci 2017), Thomas and Azmitia (2019) refined and validated the Motivation for Solitude Scale, which measures two distinct motivations. The self‐determined solitude (SDS) subscale captures intrinsic motivations for solitude, such as self‐attunement, contemplation, and leisure, whereas not self‐determined solitude (NSDS) captures extrinsic motivations, such as peer rejection and social anxiety. In ESM research across the adult lifespan, self‐determined or autonomous motivations for solitude have been linked with experiencing solitude more positively in the moment, and not self‐determined motivations such as social anxiety with experiencing solitude more negatively (e.g., Lay et al. 2019; Long and Averill 2003; Nguyen, Ryan, and Deci 2018). To the extent that self‐concepts bias retrospective reports in line with expectations about social situations, we expect that individuals with higher SDS would retrospectively exaggerate how good they felt in solitude.

### 1.3 Affect Recall Biases Across Cultures

In addition to personality‐related beliefs, culture is a key source of self‐concepts that shape affect recall. We focus here on independent and interdependent self‐construal (SC) (Markus and Kitayama 1991), primary dimensions on which comparisons are made across and within cultures. Members of more individualistic cultures (e.g., Western Europe, North America) tend to have a more independent SC, seeing themselves in terms of more stable, internal personal attributes that are independent of their relationships with others (Suh 2002). Members of more collectivistic cultures (e.g., East Asia, Latin America) tend instead to have more interdependent SC, seeing themselves in terms of more fluid, context‐dependent personal attributes that are defined in relation to social roles (Suh 2002).

Independent SC has been linked with motivation to pursue personal happiness (e.g., Kitayama, Markus, and Kurokawa 2000) and to be consistent across social contexts (English and Chen 2007). Together, these motivations may produce an overall positivity bias in memory. For example, in a study of US university students, European Americans (but not Asians) exaggerated their positive affect in retrospective as compared to concurrent reports (Oishi 2002). Moreover, in an adult lifespan sample, White Americans retrospectively exaggerated their high‐arousal positive affect (HAPA) more than non‐White Americans (Lay et al. 2017). Hence, people with a more independent SC may retrospectively exaggerate how good they felt *overall*.

Individuals with a more interdependent SC may have a self‐concept that varies more across social contexts, but be motivated to be consistent within a given context (English and Chen 2007). For example, East Asian university students in one study were more likely than American students to report being both extraverted and introverted (Choi and Choi 2002). In an ESM study comparing affective experiences when alone and when with various social partners, Japanese and Hispanic‐American individuals showed more affect variability across these different situations compared with a broader US sample (Oishi et al. 2004). Hence, among individuals with a more interdependent SC, affective experiences seem to be more strongly shaped by social context (e.g., being in solitude versus being with others), compared with individuals with a more independent SC. We suggest that this situational specificity of affective experiences may be magnified in retrospective reports, such that individuals with a more interdependent SC will show greater biases when recalling how they felt in specific social contexts (such as solitude).

### 1.4 Affect Recall in Solitude Across Cultures

How might independent/interdependent SC shape affect recall for solitude? In line with cultural norms emphasizing individual autonomy and distinctiveness, individuals higher in independent SC and lower in interdependent SC might remember solitude more positively. Participants in one cross‐sectional study recalled the frequency with which they felt various emotions; for Japanese undergraduates, positive emotions and interpersonally engaged emotions (e.g., closeness) were more strongly linked, whereas for US American undergraduates, positive emotions and interpersonally *dis*engaged emotions (e.g., pride) were more strongly linked (Kitayama, Markus, and Kurokawa 2000). This also aligns with previous research linking unsociability or unsociable behavior (non‐fearful preference for solitude) with maladaptive outcomes among Chinese but not among North American children (e.g., Liu et al. 2015).

However, research using trait self‐reports has suggested the opposite, that there may exist a positive association between interdependent SC and positive recall of solitude. In one study, South African adolescents from collectivistic (African) cultures scored *higher* in SDS than those from individualistic (European) cultures (van Zyl, Dankaert, and Guse 2018). In another study, Chinese adolescents reported more positive attitudes toward being alone than did Belgian adolescents (Maes et al. 2016). Such positive evaluations of being alone may be linked with an appreciation for quiet contemplation which is rooted in Taoist and Confucian traditions among East Asians (Averill and Sundararajan 2014). Moreover, solitude is conducive to low‐arousal forms of positive affect, which are more preferred among East Asians than among European Americans (whereas European Americans have a stronger preference for high‐arousal positive affect; Tsai, Knutson, and Fung 2006).

If we turn from retrospective reports to concurrent reports of how individuals actually feel in moments of solitude, previous ESM findings are mixed regarding cultural differences. In a study of university students, Hispanic Americans felt better when alone, but Japanese felt worse when alone, compared with a broader US sample (Oishi et al. 2004). In studies comparing North American and East Asian older adults, one linked Chinese culture with feeling *less* badly in moments of solitude (Jiang et al. 2019), and one showed no such cultural differences (Lay et al. 2020). Although no previous research has examined whether SC shapes how we recall feeling in solitude, on balance, we may expect individuals with a more interdependent SC to retrospectively report feeling better in solitude than their concurrent reports would suggest.

### 1.5 Present Study

The aim of the present (registered report) study is to examine biases in individuals' recall of how they feel overall (across social situations) and in situations of solitude, and the moderating roles of self‐concepts (introversion, SDS, NSDS, independent SC, and interdependent SC) in recall biases. Individuals living in the UK and in HK reported their current social situations and affective states five times daily for 7 days and then retrospectively reported how they felt overall and when in solitude over this 7‐day period. Retrospective reports were compared with mean concurrent reports to derive indices of affect recall accuracy. By sampling across these two locations, we aim to capture a range of self‐concepts exhibited among Western Europeans and East Asians, and to explore whether place of residence exhibits similar effects as independent/interdependent SC.

We made seven hypotheses (H1–H7). We hypothesized that individuals would overreport their overall high‐arousal positive affect (HAPA) and high‐arousal negative affect (HANA) in retrospective as compared to concurrent reports (H1), and we did not hypothesize any overall recall bias for low‐arousal positive and negative affect (LAPA, LANA). We also expected that self‐concepts would moderate overall affect recall bias. Specifically, we hypothesized that higher introversion (H2) would be positively associated with underreporting HAPA and overreporting LAPA, HANA, and LANA, in line with previous research on introversion and affect retrospection (e.g., Lay et al. 2017; Mill, Realo, and Allik 2016). We further hypothesized that higher NSDS (H3) would be positively associated with underreporting HAPA and LAPA and overreporting HANA and LANA, given links between NSDS and negative affectivity (Thomas and Azmitia 2019). We also hypothesized that higher independent SC (H4) would be positively associated with overreporting HAPA and underreporting LAPA, HANA, and LANA overall, given previous research linking independent SC with positivity bias in recall (and bias specific to HAPA), preference for high‐arousal over low‐arousal positive affect, and motivation for consistency across contexts (English and Chen 2007; Kitayama, Markus, and Kurokawa 2000; Lay et al. 2017; Tsai, Knutson, and Fung 2006).

When recalling time in solitude specifically, we hypothesized that individuals would retrospectively underreport their HAPA and HANA and overreport their LAPA and LANA (H5), in line with solitude's affect deactivating effects (e.g., Nguyen, Ryan, and Deci 2018). We also hypothesized that higher SDS (H6) and higher interdependent SC (H7) would be associated with further overreporting of LAPA and underreporting of HANA in solitude, in line with beliefs about enjoying solitude common to both these traits, and with interdependent SC‐related preference for low‐arousal positive affect and motivation for consistency within social contexts (English and Chen 2007; Thomas and Azmitia 2019; Tsai, Knutson, and Fung 2006).

To account for well‐established episodic memory influences on affect recall (i.e., peak and recency effects), models controlled for peak (maximum) affect and recent (last day) affect for all affect measures over the ESM period, and for time elapsed from the ESM period to the retrospective report (e.g., Fredrickson 2000; Robinson and Clore 2002). Models also controlled for current affective states at the time of retrospection, given previous research suggesting affect recall may be biassed by current affect (Brose, Lindenberger, and Schmiedek 2013). Finally, models controlled for sociodemographical variables associated with affect recall accuracy; for example, older age has been linked with a positivity bias (or reduced negativity bias) in affect recall (Lay et al. 2017; Robinson and Clore 2002).

## 2 Pilot Study

Prior to our main (pre‐registered) study, we conducted a pilot study (Jan–Apr 2021) with a UK university student sample whose data partially address our hypotheses. Participants provided daily time‐use reports of positive and negative affective states and social situations over 14 days. For each affective state, they then retrospectively estimated (a) their average affect over the 14 days and (b) how much their affect had *differed* from this average on days when they had spent more time in solitude. Models predicted retrospective affect reports from mean daily affect reports to assess retrospective report accuracy and examined whether introversion, SDS, NSDS, independent SC, and interdependent SC shape retrospective reports. This pilot study provided initial evidence for the role of self‐concepts in affect recall, linking higher NSDS with recalling lower energy overall and higher independent SC with recalling higher tiredness overall. Higher introversion was also associated with recalling lower stress on high‐solitude days and higher independent SC with recalling lower loneliness on high‐solitude days. We report the full pilot study methods, results, and discussion in Supplementary Materials A. The pilot study led to refinements in the study design and measures for the main study (reported below), which uses concurrent assessments administered five times daily instead of daily time‐use reports.

## 3 Method

### 3.1 Sample

For the main study, we recruited community‐dwelling participants aged 18–49 years in the UK (SW England and Scotland, *N* = 160) and in HK SAR (*N* = 159). This upper age cutoff was to target working‐age adults acquainted with smartphone technology. Samples were age‐stratified (age groups 18–25, 26–33, 34–41, and 42–49) and represent diverse educational and socioeconomic backgrounds reflecting each location's demographics (Office for National Statistics 2017; Hong Kong Census and Statistics Department 2017). Participants were recruited via newspaper, posters, participant databases, and word of mouth. Participants were excluded if they did not own a smartphone, were unable to read and write in either English or Chinese, or had a major psychological or neurocognitive diagnosis (e.g., major depression or stroke). Ethics approval was obtained from the university research ethics committees at our study sites (University of Exeter, University of Strathclyde, Education University of Hong Kong, and Lingnan University).

Table 1 reports sociodemographic variables for the UK and HK samples. Eighty‐six participants (27%) were born in a country other than their current location of residence. Missing data for sociodemographic covariates (income: *N* = 3, education: *N* = 1, marital status: *N* = 1) were estimated via multiple imputation based on the other sociodemographic covariates used in the planned analyses (10 imputed datasets using *mice* package in *R*; van Buuren and Groothuis‐Oudshoorn 2011). Participants in HK (vs. UK) were more likely to be Asian and reported less agreement with the statement, “Were your days in the study typical of your daily life?” when asked in the study post‐survey.

**TABLE 1:** Participant sociodemographics for United Kingdom (UK) and Hong Kong (HK) samples.

	UK sample (*N* = 160)	HK sample (*N* = 159)	Sample comparison
Age	*M* = 32.8, *SD* = 9.3, *Range* = 18–49 years	*M* = 33.0, *SD* = 8.9, *Range* = 18–49 years	*t*(317) = 0.26, *p* = 0.791
Gender	Female: 53.1% Male: 46.9%	Female: 61.0%, Male: 39.0%	*Χ* ^*2*^(1) = 1.71, *p* = 0.191
Ethnicity	European: 68.1%, South Asian: 16.9%, African/Caribbean: 6.9%, East Asian: 5.0%, Native: 1.3%, Southeast Asian: 0.6%	East Asian: 99.4%, Central Asian: 0.6%	**Asian: *Χ*** ^***2***^ **(1) = 191.92, *p* < 0.001**
Employment	Working FT: 50.0%, Student FT: 28.8%, Homemaking/caretaking: 8.1%, Furlough: 0.0%	Working FT: 52.8%, Student FT: 20.1%, Homemaking/caretaking: 3.8%, Furlough: 9.4%	Working full‐time: *Χ* ^*2*^(1) = 1.06, *p* = 0.304
Education	Primary/secondary: 1.3%, Secondary diploma: 32.0%, Some post‐secondary: 13.8%, Undergrad degree: 46.3%, Grad/professional degree: 18.1%	Primary/secondary: 8.8%, Secondary diploma: 1.9%, Some post‐secondary: 28.3%, Undergrad degree: 25.8%, Grad/professional degree: 35.2%	Post‐secondary: *Χ* ^*2*^(1) = 5.98, *p* = 0.015
Relationship status	Single: 29.6%, In a relationship: 31.4%, Married: 37.1%, Separated/divorced: 1.3%, Widowed: 0.3%	Single: 34.0%, In a relationship: 27.0%, Married: 39.0%, Separated/divorced: 0.0%, Widowed: 0.0%	Married: *Χ* ^*2*^(1) = 0.55, *p* = 0.460
Years in current country	*M* = 26.6, *SD* = 13.3, *Range* = 0–49	*M* = 29.2, *SD* = 12.0, *Range* = 1–49	*t*(317) = 1.83, *p* = 0.069
Living situation	With partner: 48.8%, With other family: 58.1%, With friends/acquaintances: 18.8%, Alone: 10.0%	With partner: 38.4%, With other family: 44.2%, With friends/acquaintances: 15.9%, Alone: 6.9%	Living alone: *Χ* ^*2*^(1) = 0.62, *p* = 0.431
Monthly income before tax	0–2000 GBP: 19.7%, 2001–4000 GBP: 34.5%, 4001–6000 GBP: 23.0%, >6000 GBP: 22.9%	0–8500 HKD: 7.6%, 8501–20,000 HKD: 18.2%, 20,001–59,999 HKD: 51.0%, >= 60,000 HKD: 23.2%	At or above median: *Χ* ^*2*^(1) = 1.53, *p* = 0.217
Perceived social status	*M* = 5.61, *SD* = 1.63, *Range* = 1–10	*M* = 5.36, *SD* = 1.60, *Range* = 1–10	*t*(317) = −1.37, *p* = 0.172
Physical health rating	*M* = 3.31, *SD* = 1.02, *Range* = 1–5	*M* = 3.21, *SD* = 1.01,*Range* = 1–5	*t*(317) = −0.92, *p* = 0.357
Mental health rating	*M* = 3.01, *SD* = 1.09, *Range* = 1–5	*M* = 3.09, *SD* = 1.01, *Range* = 1–5	*t*(317) = 0.75, *p* = 0.454
Overall well‐being rating	*M* = 3.24, *SD* = 0.97, *Range* = 1–5	*M* = 3.06, *SD* = 0.98, *Range* = 1–5	*t*(317) = −1.71, *p* = 0.088
Days in study typical of daily life?	*M* = 3.99, *SD* = 0.96, *Range* = 1–5	*M* = 3.50, *SD* = 0.99, *Range* = 1–5	***t*(317) = −4.51, *p* < 0.001**

*Note*: Bold values indicate statistical significant at *p* < 0.01.

### 3.2 Procedure

The study was completed online and via participants' smartphones. Interested participants were directed to an online pre‐survey consisting of informed consent and trait measures. Participants were then instructed how to download the ESM app (PIEL survey; Jessup et al. 2012) and questionnaire script to their smartphone. Each day for the next 7 days, the app prompted participants to complete five questionnaires (of about 5 min in length) asking about their current affect and social situations. Questionnaires were administered on a pseudo‐randomized schedule (random time within each of five 2‐h blocks throughout the day, with at least 1 h between blocks). To maximize compliance, participants were offered a choice of schedules to accommodate their daily routines and were sent two notification reminders (after 10 and after 20 min) for missed questionnaires.

After the 7‐day ESM period, participants emailed their ESM data to the research team. Participants then completed an online post‐survey consisting of retrospective affect and trait measures, study feedback, and debriefing. Participants were reimbursed 30 GBP (UK sample) or 300 HKD (HK sample) for completing the pre‐survey, post‐survey, and at least 25 of the 35 ESM questionnaires, with pro‐rated reimbursement for partial completion.

### 3.3 Power Analysis and Data Inclusion

Our a priori power analysis was based on previous research examining relationships between self‐concepts (e.g., Extraversion) and discrepancies between retrospective and mean momentary affect reports (e.g., Lay et al. 2017; Mill, Realo, and Allik 2016) which has reported between‐person effect sizes of approximately 0.1 to 0.3, and likewise for our pilot study. We conducted a power analysis via simulation in *R* using a template from Kirtley et al. (2021), for a linear mixed effects model with autocorrelation and a correlated random intercept and random slope. By conducting 10,000 Monte Carlo simulations, we determined our planned sampling design (300 participants × 35 ESM assessments) was sufficient to detect between‐person effect sizes of *β* = 0.1 (*f* ^*2*^ = 0.01) with power = 0.8, *α*_2‐tailed_ = 0.05. We exceeded the planned sample size by recruiting and retaining 319 participants; this over‐sampling was done to ensure a sufficient sample size even after participant exclusions (e.g., due to missing data on key variables). All participants provided data on the key person‐level variables (retrospective and mean momentary affect overall and in solitude, self‐concepts); hence, none were excluded.

Participants with missing data for ESM assessments were included in analyses if they provided data for at least 3 separate days. Of the 319 study participants, 303 (95.0%) completed at least 7 days of ESM assessments. Participants each completed an average of 30.8 of the 35 assessments (*SD* = 11.0) over the ESM period, a completion rate of 88% (in line with expected completion rates of 88%–93% based on previous research using similar ESM schedules with adult lifespan samples; Lay et al. 2017, 2019). A total of 11,941 ESM assessments were collected across the sample; of these, 3193 (26.7%) captured instances of solitude. Each participant reported an average of 9.5 solitude instances (*SD* = 7.3, *Range* = 1–43), slightly lower than our initial estimate (based on previous adult lifespan studies; Larson 1990; Pauly et al. 2017) that each participant would be in solitude at least 30% of the time and hence provide data on at least 10 solitude instances.

### 3.4 Measures

ESM measures were administered during the 7‐day study period via smartphone, and trait measures were administered in the online pre‐ and post‐surveys. The measures used in the analysis are described below; in addition to these, the full set of study measures are listed on the study OSF page. We used validated English and Hong Kong Chinese versions of each measure when possible. When Chinese measures did not exist in the literature, these were translated from English using a standardized forward and backward translation procedure with two bilingual researchers. Participants had the choice to complete the study in English, traditional Chinese, or simplified Chinese.

#### 3.4.1 Experience Sampling Method (ESM) measures

##### 3.4.1.1 Momentary Affect

Participants were asked, at each assessment, to report their current affective states (“At this moment, I feel ____”) on a scale from 0 (“not at all”) to 10 (“very much”). Affect items included high‐arousal positive states (“happy” and “energized”), low‐arousal positive states (“calm” and “relaxed”), high‐arousal negative states (“irritated” and “anxious”), and low‐arousal negative states (“lonely” and “tired”), in line with validated cross‐cultural measures based on the affect circumplex model (Russell 1980; Tsai, Knutson, and Fung 2006). Items were updated from those used in the pilot study; specific items (e.g., “stressed”) were removed due to translation difficulties, and items were added to better differentiate high‐ and low‐arousal affective states. Composite scores were computed at each assessment by taking the mean of the respective items for HAPA (*M* = 5.3, *SD* = 1.5), LAPA (*M* = 5.9, *SD* = 1.5), HANA (*M* = 2.9, *SD* = 1.6), and LANA (*M* = 3.8, *SD* = 1.4). Between‐ and within‐person reliability coefficients were computed for each composite (Lai 2021; Shrout and Lane 2012): HAPA between‐person reliability (*R*_*bet*_) = 0.96, within‐person reliability (*R*_*wit*_) = 0.49; LAPA *R*_*bet*_ = 0.96, *R*_*wit*_ = 0.50; HANA *R*_*bet*_ = 0.97, *R*_*wit*_ = 0.61; LANA *R*_*bet*_ = 0.92, *R*_*wit*_ = 0.55.

Each participant's *mean momentary affect* was computed for HAPA, LAPA, HANA, and LANA by taking the mean of their momentary affect scores over the 7‐day ESM period. *Peak affect* for HAPA, LAPA, HANA, and LANA was derived from the participant's maximum affect scores over the 7‐day period. *Recent affect* for HAPA, LAPA, HANA, and LANA was computed as the mean of the participant's affect scores on the last ESM day with available data. Descriptive statistics are in Supplementary Materials B, Table S4.

##### 3.4.1.2 Current Social Context

At each assessment, participants were asked, “In the last 15 min, which of these in‐person situations have you been in?” and given the response options, (a) “Alone (no one nearby, no one you can see),” (b) “One or more people nearby who you were NOT interacting with in‐person,” and (c) “One or more people nearby who you WERE interacting with in‐person.” Participants could select one or more responses; instances in which they selected only option (a) were coded as *alone* (29.5% of assessments), and instances in which they selected only (a) and/or (b) as *in‐person solitude* (45.1% of assessments). Instances in which participants selected option (c), regardless of whether other options were selected, were coded as *in‐person interaction*, and a follow‐up question asked who they were interacting with, with response options, “Spouse/partner,” “Family member,” “Friend,” “Colleague/classmate,” “Service provider,” “Stranger,” and “Other.”

Participants were then asked, “In the last 15 min, which of these virtual situations have you been in?”, with the response options, (a) “Video/phone interaction,” (b) “Texting (e.g., WhatsApp, WeChat),” (c) “Active social media use (e.g., posting or commenting on Facebook, Instagram, Snapchat),” (d) “Passive social media use (e.g., scrolling or liking others' posts),” and (e) “None of the above.” Instances in which participants selected only (d) and/or (e) were coded as *virtual solitude* (54.4% of assessments). Selecting either (a), (b), or (c) was coded as *virtual interaction*, and a follow‐up question was shown asking who they were interacting with virtually (same response options given as for in‐person interaction).

For analyses, instances coded as both in‐person and virtual solitude were considered instances of *solitude* (no in‐person or virtual interaction; 26.7% of assessments). *Proportion of time in solitude* was computed for each participant as the proportion of ESM beeps when they had been in solitude in the last 15 min (*M* = 28.5%, *SD* = 21.9, *Range* = 0.0–96.3). For each participant, *mean momentary HAPA in solitude* was computed by averaging their scores for HAPA across solitude instances (*M* = 4.9, *SD* = 1.7), and likewise for LAPA (*M* = 5.9, *SD* = 1.7), HANA (*M* = 2.9, *SD* = 1.9), and LANA (*M* = 3.9, *SD* = 1.6). *Peak HAPA in solitude* was derived from the participant's maximum HAPA scores in solitude, and *recent HAPA in solitude* from their mean HAPA scores in solitude on the last day of the ESM period (or the last day with any solitude instances), and likewise for LAPA, HANA, and LANA. Descriptive statistics are in Table SM4. Analyses involving affect in solitude exclude 29 participants who did not report any solitude situations during the ESM period.

#### 3.4.2 Pre‐ and Post‐Survey Measures

##### 3.4.2.1 Current Affect at Retrospection

In the post‐survey, participants first reported their affect via eight items of the form “How [happy] do you feel right now,” from 0 (not at all) to 10 (very much), and likewise for energized, calm, relaxed, irritated, anxious, lonely, and tired. Affective states match those used in the ESM measures, and composite scores were derived for current HAPA (*α* = 0.50), LAPA (*α* = 0.68), HANA (*α* = 0.53), and LANA (*α* = 0.31).

##### 3.4.2.2 Retrospective Affect

In the post‐survey, participants were then asked to recall their affective experiences over the 7‐day ESM period. The first eight items were of the form, “How [happy] did you feel, on average?”, on a 0–10 scale, with affective states matching those used in the momentary affect measures. *Overall retrospective HAPA* (*M* = 5.6, *SD* = 1.2, *α* = 0.60), *LAPA* (*M* = 5.9, *SD* = 2.0, *α* = 0.75), *HANA* (*M* = 3.4, *SD* = 2.1, *α* = 0.51), and *LANA* (*M* = 4.2, *SD* = 1.9, *α* = 0.33) scores were computed by averaging the items in each scale. The next eight items were of the form, “How [happy] did you feel, on average, at times when you were not interacting with anyone in person or virtually?”, with the same set of affective states (0–10 scale). A score for *retrospective HAPA in solitude* (*M* = 5.2, *SD* = 2.0, *α* = 0.57) was derived by averaging the HAPA items, and likewise for LAPA (*M* = 6.3, *SD* = 2.2, *α* = 0.76), HANA (*M* = 2.6, *SD* = 2.1, *α* = 0.62), and LANA (*M* = 3.8, *SD* = 2.3, *α* = 0.37). Analyses of retrospective irritation in solitude exclude one participant who skipped this item.

Eight additional items were of the form “How [happy] did you feel, on average, at times when you were interacting in‐person with someone you are close to” on a 0–10 scale; an aggregate score was computed for *retrospective HAPA close interaction* (*M* = 6.4, *SD* = 1.2, *α* = 0.53) and likewise for LAPA (*M* = 6.6, *SD* = 2.0, *α* = 0.67), HANA (*M* = 2.3, *SD* = 1.8, *α* = 0.55), and LANA (*M* = 2.8, *SD* = 1.7, *α* = 0.23). Eight final items were of the form “How [happy] did you feel, on average, at times when you were interacting in‐person with someone you are NOT close to” (0–10 scale); a score was computed for *retrospective HAPA not‐close interaction* (*M* = 4.3, *SD* = 2.0, *α* = 0.65) and likewise for LAPA (*M* = 4.6, *SD* = 2.2, *α* = 0.63), HANA (*M* = 3.5, *SD* = 2.2, *α* = 0.49), and LANA (*M* = 3.9, *SD* = 2.1, *α* = 0.42).

We computed retrospective affect report discrepancies by subtracting each participant's mean momentary affect score from their respective retrospective affect score for each affect scale: overall HAPA (*α* = 0.36), overall LAPA (*α* = 0.47), overall HANA (*α* = 0.39), overall LANA (*α* = 0.36), HAPA in solitude (*α* = 0.48), LAPA in solitude (*α* = 0.64), HANA in solitude (*α* = 0.40), and LANA in solitude (*α* = 0.27). Descriptive statistics are in Table SM4.

##### 3.4.2.3 Introversion

The 8‐item Extraversion‐Introversion scale from the Big Five Inventory (John, Donahue, and Kentle 1991) was administered in the pre‐survey. Participants responded on a 5‐point scale to items including “I see myself as someone who is outgoing, sociable.” Responses were reverse‐coded and averaged to derive a score for each participant, with higher scores indicating greater introversion (*M* = 3.2, *SD* = 0.8, *α* = 0.80).

##### 3.4.2.4 Self‐Determined and Not‐Self‐Determined Motivation for Solitude

The Motivation for Solitude Scale (MSS; Thomas and Azmitia 2019) was administered in the post‐survey to assess two types of solitude‐seeking motivations based on self‐determination theory. Items are of the form “When I spend time alone, I do so because…,” on a 5‐point scale. Eight items assessed SDS, for example, “I enjoy the quiet” (*M* = 2.7, *SD* = 0.7, *α* = 0.83), and six items assessed NSDS, for example, “I feel uncomfortable when I'm with others” (*M* = 1.9, *SD* = 0.7, *α* = 0.87); higher scores indicate higher SDS/NSDS.

##### 3.4.2.5 Independent and Interdependent SC

The Cultural Orientation scale (Triandis and Gelfland 1998) was administered in the post‐survey to capture vertical and horizontal dimensions of individualism and collectivism. We used the scale's individualism and collectivism dimensions to measure independent and interdependent SC, respectively, at the person level (Guo, Schwartz, and McCabe 2008). Participants responded to items on a 6‐point scale; eight items assessed independent SC, for example, “I often ‘do my own thing’” (*M* = 4.2, *SD* = 0.6, *α* = 0.61), and eight items assessed interdependent SC, for example, “I feel good when I cooperate with others” (*M* = 4.3, *SD* = 0.7, *α* = 0.78), with higher scores indicating higher independent/interdependent SC.

##### 3.4.2.6 Sociodemographics

Sociodemographic variables were assessed in the pre‐survey: age (years), gender (1 = Male, 0 = Female), location (1 = HK, 0 = UK), years lived in current country, ethnicity (1 = Asian, 0 = White or other ethnicities), living situation (1 = alone, 0 = not alone), subjective social status (same as pilot study; Adler and Stewart 2007), marital status (1 = married, 0 = not married), education (1 = at least some post‐secondary, 0 = no post‐secondary), and monthly income before tax (1 = at or above sample median, 0 = below median). Descriptive statistics are provided in Table 1 (for each sample separately) and in Supplementary Materials B, Table S4 (for the two samples combined).

### 3.5 Analysis Plan

#### 3.5.1 Analytical Approach and Data Checks

We conducted the confirmatory and exploratory analyses described below using a significance level alpha of 0.01 to adjust for multiple tests. Hence, results significant at *p* < 0.01 are reported as main study findings, whereas those significant at *p* < 0.05 are interpreted only as being suggestive of relationships. We examined bivariate correlations among all study variables; when predictors were correlated at *r* > 0.7, we conducted sensitivity analyses by including and excluding each predictor in the reported models. ESM assessments completed beyond the 7‐day study period were also excluded in sensitivity analyses.

#### 3.5.2 Planned Confirmatory Analyses

To test Hypothesis 1 (overall retrospective report biases), we examined regression models predicting retrospective HAPA minus mean momentary HAPA, and likewise for the other affect scales. If model intercepts (*β*_0_) were significantly different from 0, this indicated a retrospective report discrepancy, with positive scores indicating retrospective overreporting, and negative scores indicating retrospective underreporting, of affective states. We tested Hypotheses 2–4 regarding self‐concept predictors of retrospective report biases by adding self‐concept predictors to the models: Introversion (H2), NSDS (H3), and Independent SC (H4). We also included SDS and Interdependent SC in models, along with covariates: peak (maximum) affect, recent affect, and current affect at retrospection for the respective outcome (HAPA, LAPA, HANA, or LANA); days elapsed from last ESM assessment to post‐survey, age, gender, ethnicity, location of residence, years in current country, living situation, marital status, education, income, and subjective social status.

To test H5 (retrospective report biases in solitude), we examined models predicting the difference between retrospective and mean momentary reports of affect when in solitude for HAPA, LAPA, HANA, and LANA. Model intercepts (*β*_0_) that were significantly different from 0 indicate a retrospective report discrepancy for affect in solitude. Hypotheses 6–7 pertaining to self‐concepts were tested by adding SDS (H6) and Interdependent SC (H7) to the above models, alongside introversion, NSDS, and Independent SC. We included the same covariates used in the models predicting overall affect discrepancies, except that peak affect and recent affect were computed based only on instances of solitude, and models also controlled for the individual's proportion of ESM assessments spent in solitude.

#### 3.5.3 Planned Exploratory Analyses

We broke down the HAPA, HANA, LAPA, and LANA scales into their constituent affect items and conducted parallel analyses examining retrospective report discrepancies for each item. This enabled us to pinpoint which affective states (e.g., “lonely” versus “tired”) were impacted by retrospection and self‐concepts (e.g., Mill, Realo, and Allik 2016), which can provide important theoretical insights given solitude's links with specific affective states. For example, loneliness is a well‐documented correlate of solitude that has negative well‐being implications, whereas tiredness in solitude may reflect a more benign need for restful reprieve (e.g., Larson 1990). Moreover, the low Cronbach's alphas for the retrospective report discrepancy scales (ranging from *α* = 0.27 for LANA in solitude to *α* = 0.64 for LAPA in solitude) suggest that the constituent affect items should indeed be examined separately in our sample. We also included ethnicity, location of residence, and years in current country in our models to disentangle effects of independent and interdependent SC from effects of ethnicity, British vs. Chinese cultural exposure, and immigration (e.g., Lay et al. 2020).

Next, we conducted descriptive analyses comparing two sub‐types of solitude—“solitude alone” (no one physically present) and “solitude with others” (others present but no social interaction)—in terms of their associations with specific affective states and self‐concepts. This enabled us to examine, for example, whether solitude with others was experienced more positively than solitude alone, or was experienced more frequently among high‐extraversion individuals, potentially to maintain social connection (McGonigal 2011).

Finally, in addition to examining affect recall for situations of solitude, we examined a parallel set of models for situations of in‐person social interaction, to determine whether findings were specific to solitude or were related to recall of affect during social interaction.

## 4 Results

### 4.1 Descriptive Analyses

Bivariate correlations are presented in Supplementary Materials B, Table S4. Here, we highlight theoretically meaningful correlations (*p*s < 0.01, with |*r*|s of 0.15–0.36). SDS was positively correlated with retrospective report discrepancies for LAPA in solitude. Retrospective discrepancies for LANA in solitude were positively correlated with interdependent SC and negatively correlated with older age and years in current location of residence, and were higher for individuals living in the UK (HK *M* = −0.6, UK *M* = 0.4) and of non‐Asian ethnicity (Asian *M* = −0.4, Non‐Asian *M* = 0.4). Introversion was positively correlated with NSDS but not with SDS. NSDS and SDS were positively correlated. Independent SC was positively correlated with both SDS and NSDS, and interdependent SC was negatively correlated with both NSDS and introversion. When examining peak and recent affect overall and in solitude, and current affect at retrospection, not all associations with affect variables were significant, but overall patterns linked higher introversion, NSDS, and independent SC with lower positive affect and higher negative affect, and linked higher SDS and interdependent SC with higher positive affect and lower negative affect.

Individuals living in HK (vs. UK) had higher introversion (HK *M* = 3.3 vs. UK *M* = 3.0), SDS (2.9 vs. 2.6), NSDS (2.1 vs. 1.7), and independent SC (4.3 vs. 4.0), and lower interdependent SC (4.2 vs. 4.5), and they tended to report lower recent HAPA and LAPA overall, and lower recent HANA in solitude. A similar but less‐consistent pattern was found for individuals of Asian (vs. non‐Asian) ethnicity; they tended to have higher introversion (Asian *M* = 3.3 vs. Non‐Asian *M* = 2.9), SDS (2.9 vs. 2.5), NSDS (2.1 vs. 1.7), and independent SC (4.3 vs. 4.0), and lower recent HAPA and LAPA overall. Individuals with higher rates of solitude had higher introversion, lower peak HAPA and LAPA overall, higher peak HANA and LANA in solitude, and lower recent HAPA in solitude.

### 4.2 Data Checks and Planned Confirmatory Analyses

Regression models predicted retrospective report discrepancies for overall and solitude‐specific affective experiences. Models included all planned covariates with two exceptions: (1) Because location of residence and ethnicity (coded 1 = East, Southeast, or South Asian; 0 = other ethnicities) were strongly correlated (*r* = 0.78, with Asian participants mainly residing in HK), we retained only location of residence as a covariate. (2) Because age and years in current country were strongly correlated (*r* = 0.72), we retained only age as a covariate. We conducted sensitivity analyses by replacing location of residence with ethnicity, and age with years in current country, in study models (findings reported below). All predictors (including covariates) were grand mean centered; hence, model intercepts capture overall retrospective report discrepancies. There were no outliers (>3 *SD* from the mean) for any variables. Some ESM assessments (15.1%) were completed after the planned 7‐day period, so we conducted sensitivity analyses to only include each participant's last 7 days of assessments in the models; this did not substantively affect reported findings.

#### 4.2.1 Retrospective Report Discrepancies for Overall Affect

We tested Hypotheses 1–4 pertaining to retrospective discrepancies for overall affect using one regression model for each affect scale (HAPA/LAPA/HANA/LANA), reported in Table 2. Full results for the eight constituent affective states are reported in Supplementary Materials B, Tables S5–S12. We first hypothesized that individuals would retrospectively overestimate their overall HAPA (H1a) and HANA (H1b), but not their LAPA (H1c) or LANA (H1d). H1a, H1b, and H1c were confirmed; individuals retrospectively overestimated their HAPA (*b* = 0.28 on a 0–10 scale) and HANA (*b* = 0.45) and accurately reported their LAPA (*b* = −0.09). H1d was not supported, as individuals retrospectively overestimated their LANA (*b* = 0.36); examination of affective constituents of LANA showed this effect was specific to tiredness and was nonsignificant for loneliness (Tables SM11 and SM12).

**TABLE 2:** Models predicting discrepancy between retrospective and mean momentary reports of overall HAPA, LAPA, HANA, and LANA.

Variable	HAPA					LAPA					HANA					LANA				
	*b*	*β*	*p*	95% CI(l)	95% CI(u)	*b*	*β*	*p*	95% CI(l)	95% CI(u)	*b*	*β*	*p*	95% CI(l)	95% CI(u)	*b*	*β*	*p*	95% CI(l)	95% CI(u)
Intercept	**0.28**	**0.00**	**0.000**	**0.14**	**0.41**	−0.09	0.00	0.183	−0.23	0.04	**0.45**	**0.00**	**0.000**	**0.30**	**0.61**	**0.36**	**0.00**	**0.000**	**0.21**	**0.51**
Introversion (1–5)	0.06	0.03	0.586	−0.14	0.25	**0.34**	**0.20**	**0.001**	**0.14**	**0.54**	−0.03	−0.02	0.768	−0.26	0.19	0.12	0.06	0.284	−0.10	0.33
Self‐determined solitude (1–5)	0.04	0.02	0.755	−0.20	0.27	−0.03	−0.01	0.813	−0.26	0.20	0.06	0.03	0.623	−0.19	0.32	−0.12	−0.05	0.343	−0.36	0.13
Not‐self‐determined solitude (1–5)	−0.05	−0.03	0.678	−0.27	0.18	−0.23	−0.12	0.052	−0.46	0.00	0.07	0.03	0.602	−0.20	0.35	0.08	0.04	0.517	−0.17	0.34
Independent SC (1–6)	0.17	0.08	0.165	−0.07	0.42	0.06	0.03	0.641	−0.19	0.31	−0.09	−0.04	0.535	−0.38	0.20	−0.17	−0.07	0.221	−0.45	0.10
Interdependent SC (1–6)	0.02	0.01	0.878	−0.19	0.23	0.26	0.15	0.015	0.05	0.47	−0.10	−0.05	0.393	−0.34	0.13	0.14	0.07	0.220	−0.08	0.36
Peak affect (0–10)	**−0.18**	**−0.18**	**0.007**	**−0.30**	**−0.05**	−0.17	−0.14	0.020	−0.31	−0.03	0.04	0.07	0.325	−0.04	0.13	0.03	0.04	0.527	−0.07	0.14
Recent affect (0–10)	−0.06	−0.10	0.167	−0.15	0.03	−0.02	−0.03	0.650	−0.11	0.07	**−0.29**	**−0.39**	**0.000**	**−0.40**	**−0.19**	**−0.28**	**−0.37**	**0.000**	**−0.38**	**−0.18**
Current affect (0–10)	**0.20**	**0.33**	**0.000**	**0.12**	**0.28**	**0.18**	**0.32**	**0.000**	**0.11**	**0.25**	**0.25**	**0.36**	**0.000**	**0.16**	**0.35**	**0.27**	**0.42**	**0.000**	**0.20**	**0.35**
Days since ESM	−0.00	−0.03	0.573	−0.02	0.01	−0.01	−0.10	0.080	−0.03	0.00	0.01	0.06	0.291	−0.01	0.03	0.01	0.04	0.506	−0.01	0.02
Age (years)	−0.00	−0.02	0.761	−0.02	0.01	0.00	−0.03	0.577	−0.02	0.01	−0.01	−0.06	0.326	−0.03	0.01	−0.02	−0.13	0.028	−0.04	0.00
Gender (1 = male)	−0.37	−0.14	0.017	−0.67	−0.07	**−0.44**	**−0.16**	**0.005**	**−0.75**	**−0.13**	−0.30	−0.10	0.087	−0.64	0.04	0.13	0.04	0.429	−0.19	0.45
Location (1 = HK)	0.25	0.10	0.126	−0.07	0.57	0.16	0.06	0.335	−0.17	0.48	**−0.49**	**−0.16**	**0.010**	**−0.87**	**−0.12**	−0.15	−0.05	0.395	−0.50	0.20
Living alone	0.35	0.08	0.178	−0.16	0.85	0.34	0.07	0.190	−0.17	0.86	0.16	0.03	0.583	−0.42	0.75	0.20	0.04	0.486	−0.36	0.75
Social status (1–10)	−0.01	−0.02	0.753	−0.10	0.07	−0.05	−0.06	0.274	−0.14	0.04	−0.05	−0.06	0.300	−0.16	0.05	−0.04	−0.05	0.387	−0.14	0.05
Married	0.19	0.07	0.235	−0.13	0.51	0.17	0.06	0.303	−0.15	0.49	−0.16	−0.05	0.399	−0.53	0.21	−0.22	−0.07	0.203	−0.57	0.12
Education (1 = post‐secondary)	**−0.55**	**−0.16**	**0.005**	**−0.94**	**−0.17**	0.05	0.01	0.812	−0.34	0.44	0.16	0.04	0.467	−0.28	0.61	0.40	0.10	0.059	−0.01	0.82
Income (1 = at/above median)	0.13	0.05	0.393	−0.16	0.42	−0.01	0.00	0.968	−0.30	0.29	0.34	0.11	0.050	0.00	0.68	0.28	0.09	0.085	−0.04	0.60

*Note*: Positive values: retrospective report > mean momentary report; Negative values: retrospective report < mean momentary report; 0 = no discrepancy. Bold values indicate statistical significant at *p* < 0.01.

Regarding self‐concepts, we first hypothesized that introversion would be positively associated with retrospectively underreporting HAPA (H2a) and overreporting LAPA (H2b), HANA (H2c), and LANA (H2d). H2a, H2c, and H2d were not supported, but H2b was supported: Individuals higher in introversion tended to retrospectively overestimate their LAPA. We next hypothesized that NSDS would be positively associated with retrospectively underreporting HAPA (H3a) and LAPA (H3b), and overreporting HANA (H3c) and LANA (H3d). These hypotheses were not supported; associations were nonsignificant for all affect scales. Finally, we hypothesized that higher independent SC would be positively associated with retrospectively overreporting HAPA (H4a) and underreporting LAPA (H4b), HANA (H4c), and LANA (H4d) overall. None of these hypotheses were supported. However, individuals higher in *inter*dependent SC tended to retrospectively overestimate their relaxation (Table SM8).

We next examine cultural variables to inform the study results. Although location of residence showed no association with retrospective reporting of HAPA, LAPA, or LANA, individuals in HK (vs. the UK) were more prone to retrospectively overreport their overall happiness (Table SM5). When residence location was replaced with ethnicity in sensitivity analyses, Asian (vs. non‐Asian) participants were also more prone to retrospectively overreport their overall happiness (*b* = 0.50, *p* = 0.009). Individuals in the UK (but not those in HK) overreported their HANA overall; however, the specific effects for anxiety and irritation were nonsignificant (Tables SM9 and SM10). The association with HANA also lost significance when residence location was replaced with ethnicity in sensitivity analyses.

Higher peak HAPA during the ESM period was associated with less retrospective overestimation of HAPA, and higher recent HANA and LANA were associated with less retrospective overestimation of HANA and LANA, respectively (Table 2). These negative associations are due to operationalizing retrospective discrepancy as retrospective report *minus* mean of momentary reports; as peak and recent affect are positively correlated with mean momentary affect, higher peak/recent affect will result in smaller retrospective report discrepancies on average. Current HAPA, LAPA, HANA, and LANA at retrospection were associated with greater retrospective overestimation of these affective states.

#### 4.2.2 Retrospective Report Discrepancies for Affect in Solitude

We tested Hypotheses 5–7 on affect in solitude using one regression model for each affect scale (HAPA/LAPA/HANA/LANA), reported in Table 3. Results for the eight constituent affective states are in Supplementary Materials B, Tables S13–S20. We first hypothesized that, when recalling solitude, individuals would retrospectively underestimate their HAPA (H5a) and HANA (H5b) and overestimate their LAPA (H5c) and LANA (H5d). H5b and H5c were supported; participants retrospectively underestimated their HANA (*b* = −0.39) and overestimated their LAPA (*b* = 0.53) in solitude. The effect for HANA was specific to irritation and nonsignificant for anxiety (Tables S17 and S18). H5a was not supported; participants retrospectively overestimated their HAPA in solitude (*b* = 0.32). This effect was specific to energized and nonsignificant for happy (Tables S13 and S14). H5d was also not supported, as participants accurately reported their LANA in solitude (*b* = −0.13). However, analysis of affect constituents showed participants in fact retrospectively overestimated their loneliness and underestimated their tiredness in solitude (Tables S19 and S20).

**TABLE 3:** Models predicting discrepancy between retrospective and mean momentary reports of HAPA, LAPA, HANA, and LANA in solitude.

	HAPA					LAPA					HANA					LANA				
Variable	*b*	*β*	*p*	95% CI(l)	95% CI(u)	*b*	*β*	*p*	95% CI(l)	95% CI(u)	*b*	*β*	*p*	95% CI(l)	95% CI(u)	*b*	*β*	*p*	95% CI(l)	95% CI(u)
Intercept	**0.32**	**0.00**	**0.002**	**0.12**	**0.52**	**0.53**	**0.00**	**0.000**	**0.31**	**0.75**	**−0.39**	**−0.01**	**0.000**	**−0.58**	**−0.20**	−0.13	0.00	0.207	−0.34	0.07
Introversion (1–5)	0.05	0.02	0.715	−0.24	0.35	0.17	0.07	0.296	−0.15	0.49	0.00	0.00	0.995	−0.27	0.27	0.12	0.05	0.450	−0.19	0.42
Self‐determined solitude (1–5)	**0.69**	**0.25**	**0.000**	**0.34**	**1.04**	**0.88**	**0.30**	**0.000**	**0.52**	**1.24**	**−0.40**	**−0.15**	**0.009**	**−0.70**	**−0.10**	−0.32	−0.11	0.061	−0.66	0.02
Not‐self‐determined solitude (1–5)	0.02	0.01	0.900	−0.31	0.35	−0.28	−0.10	0.125	−0.63	0.08	0.18	0.07	0.244	−0.13	0.49	0.36	0.13	0.043	0.01	0.70
Independent SC (1–6)	−0.16	−0.05	0.390	−0.53	0.21	−0.18	−0.06	0.389	−0.59	0.23	0.13	0.05	0.451	−0.21	0.48	−0.13	−0.04	0.527	−0.52	0.27
Interdependent SC (1–6)	0.29	0.12	0.073	−0.03	0.60	−0.11	−0.04	0.536	−0.45	0.23	0.09	0.04	0.535	−0.20	0.38	0.36	0.14	0.025	0.05	0.68
Peak affect in solitude (0–10)	**−0.25**	**−0.25**	**0.001**	**−0.40**	**−0.11**	−0.16	−0.14	0.064	−0.33	0.01	**−0.16**	**−0.24**	**0.003**	**−0.26**	**−0.05**	−0.15	−0.17	0.048	−0.30	0.00
Recent affect in solitude (0–10)	**−0.21**	**−0.24**	**0.002**	**−0.34**	**−0.08**	**−0.20**	**−0.22**	**0.004**	**−0.33**	**−0.06**	**−0.23**	**−0.30**	**0.000**	**−0.35**	**−0.11**	−0.20	−0.20	0.013	−0.35	−0.04
Current affect in solitude (0–10)	0.12	0.15	0.033	0.01	0.24	0.14	0.17	0.013	0.03	0.25	0.22	0.28	**0.000**	**0.12**	**0.33**	**0.22**	**0.26**	**0.000**	**0.11**	**0.33**
Days since ESM	0.01	0.05	0.462	−0.01	0.03	0.00	−0.02	0.806	−0.03	0.02	−0.01	−0.07	0.271	−0.03	0.01	0.00	−0.01	0.928	−0.02	0.02
Age (years)	0.02	0.09	0.143	−0.01	0.04	−0.01	−0.04	0.554	−0.03	0.02	−0.01	−0.06	0.296	−0.03	0.01	**−0.05**	**−0.24**	**0.000**	**−0.08**	**−0.03**
Gender (1 = male)	−0.17	−0.05	0.447	−0.61	0.27	0.06	0.02	0.793	−0.42	0.55	−0.03	−0.01	0.883	−0.43	0.37	0.06	0.01	0.799	−0.39	0.50
Location (1 = HK)	−0.31	−0.08	0.193	−0.77	0.16	−0.37	−0.10	0.149	−0.88	0.14	−0.19	−0.05	0.413	−0.64	0.26	**−0.80**	**−0.21**	**0.001**	**−1.29**	**−0.32**
Living alone	0.75	0.11	0.053	−0.01	1.51	0.34	0.05	0.426	−0.50	1.18	−0.26	−0.04	0.465	−0.97	0.45	−0.15	−0.02	0.702	−0.94	0.63
Social status (1–10)	−0.04	−0.04	0.539	−0.17	0.09	−0.10	−0.08	0.180	−0.25	0.05	0.08	0.07	0.223	−0.05	0.20	**0.20**	**0.17**	**0.004**	**0.07**	**0.34**
Married	0.31	0.08	0.206	−0.17	0.78	0.27	0.06	0.314	−0.26	0.79	−0.30	−0.08	0.186	−0.75	0.15	−0.25	−0.06	0.312	−0.74	0.24
Education (1 = post‐second.)	−0.01	0.00	0.974	−0.57	0.56	0.31	0.06	0.331	−0.31	0.93	−0.39	−0.08	0.145	−0.93	0.14	−0.27	−0.05	0.354	−0.86	0.31
Income (1 = at/above median)	0.00	0.00	0.985	−0.44	0.43	0.28	0.07	0.250	−0.20	0.76	−0.06	−0.02	0.774	−0.46	0.35	−0.23	−0.06	0.302	−0.68	0.21
Proportion assessments in solitude (0–1)	0.25	0.03	0.648	−0.81	1.30	0.33	0.04	0.575	−0.81	1.47	0.91	0.12	0.081	−0.11	1.93	0.65	0.07	0.252	−0.46	1.75

*Note*: Positive values: retrospective report > mean momentary report; Negative values: retrospective report < mean momentary report; 0 = no discrepancy. Bold values indicate statistical significant at *p* < 0.01.

We next hypothesized that SDS would be positively associated with retrospectively overreporting LAPA (H6a) and underreporting HANA (H6b) in solitude; no associations were expected for HAPA (H6c) or LANA (H6d). H6a, H6b, and H6d were supported; individuals higher in SDS were more prone to overreporting their LAPA and underreporting their HANA in solitude, and SDS showed no association with retrospective discrepancies for LANA in solitude. Further examination of LANA items revealed higher SDS was associated with a lesser tendency to retrospectively overestimate loneliness in solitude, though there was no association for tiredness (Tables S19 and S20). Counter to H6c, individuals higher in SDS were more prone to overestimate their HAPA in solitude. Individuals higher in NSDS were more prone to retrospectively overestimate their loneliness in solitude (Table S19). Introversion showed no significant associations with retrospective report discrepancies for solitude.

Finally, we hypothesized that higher interdependent SC would be associated with retrospectively overreporting LAPA (H7a) and underreporting HANA (H7b) in solitude; no associations were expected for HAPA (H7c) and LANA (H7d). H7a and H7b were not supported. H7c was supported, as interdependent SC showed no significant association with HAPA report discrepancies. However, examination of HAPA constituents showed that individuals higher in interdependent SC were more prone to retrospectively overreport their energy levels in solitude, but not their happiness (Tables S13 and S14). In support of H7d, individuals higher in interdependent SC did not significantly overestimate their LANA in solitude. Further examination revealed a significant association with overreporting loneliness, but not tiredness (Tables S19 and S20). Independent SC showed no associations with retrospective report discrepancies for any affective states in solitude.

An examination of cultural and sociodemographic variables follows. Individuals living in HK (unlike those in the UK) tended to retrospectively underestimate their LANA in solitude (Table 3). This effect was specific to tiredness and nonsignificant for loneliness (Tables S19 and S20). When location of residence was replaced with ethnicity, Asian (unlike non‐Asian) individuals tended to underestimate their LANA in solitude. Again, this effect was significant for tiredness (*b* = −1.43, *p* < 0.001) but not for loneliness. Older (vs. younger) individuals were more prone to underestimate their LANA in solitude (Table 3), an effect specific to loneliness and nonsignificant for tiredness (Tables S19 and S20). Replacing age with years in current country revealed a similar pattern, with a significant effect for loneliness (*b* = −0.03, *p* = 0.008) but not for tiredness. Peak, recent, and current affect showed the same pattern of associations with retrospective discrepancies for solitude as they had for overall affect, except for peak LAPA (association was nonsignificant).

### 4.3 Planned Exploratory Analyses

We first compared two types of solitude situations: *solitude alone* (time spent alone and not engaged in in‐person or virtual interaction; 19.2% of assessments) and *solitude with others* (time spent in the physical presence of others but not engaged in in‐person or virtual interaction; 10.4% of assessments). Momentary affective states reported in each solitude situation are compared in Table 4, and relationships between person‐level variables and overall time spent in each solitude situation are shown in Table 5. Individuals reported more calm, relaxation, and loneliness in moments of solitude alone compared with solitude with others, suggesting aloneness brings more low‐arousal affect of mixed valence. Spending more time in solitude alone was associated with living in the UK (vs. HK), whereas spending more time in solitude with others was associated with living in HK (vs. the UK), living with others (vs. alone), being male (vs. female), and being more introverted.

**TABLE 4:** Momentary affect states during solitude alone (*n* = 2145) and solitude with others (*n* = 1203).

Momentary affect (0–10)	Solitude alone *M* (*SD*)	Solitude with others *M* (*SD*)	Comparison of means	Momentary affect (0–10)	Solitude alone *M* (*SD*)	Solitude with others *M* (*SD*)	Comparison of means
Happy	5.34 (2.56)	5.16 (2.37)	*t*(3347) = −2.07, *p* = 0.038	Anxious	2.94 (2.72)	3.04 (2.44)	*t*(3346) = 1.05, *p* = 0.296
Energized	4.49 (2.56)	4.67 (2.48)	*t*(3347) = 1.92, *p* = 0.055	Irritated	2.53 (2.53)	2.54 (2.41)	*t*(3346) = 0.08, *p* = 0.939
Calm	**6.11 (2.51)**	**5.74 (2.39)**	***t*(3347) = −4.16, *p* < 0.001**	Lonely	**2.84 (2.89)**	**2.41 (2.34)**	***t*(3347) = −4.47, *p* < 0.001**
Relaxed	**5.77 (2.59)**	**5.49 (2.44)**	***t*(3346) = −3.07, *p* = 0.002**	Tired	5.05 (2.88)	5.05 (2.69)	*t*(3347) = 0.02, *p* = 0.981

Bold values indicate statistical significant at *p* < 0.01.

**TABLE 5:** Relationships between person‐level variables and percentage of assessments in solitude alone and solitude with others (*N* = 319 participants).

Variable	% Sol alone	% Sol with others	Variable	Comparing % assessments solitude alone	Comparing % assessments solitude with others
Introversion	*r* = 0.07 *p* = 0.210	***r* = 0.16, *p* = 0.003**	Location	**UK: 22.6%, HK 15.9%, *t*(310) = −3.10, *p* = 0.002**	**UK: 8.7%, HK 12.1%, *t*(297) = 2.70, *p* = 0.007**
SDS	*r* = −0.10, *p* = 0.085	*r* = −0.02, *p* = 0.715	Ethnicity	Asian: 21.0%, Other: 18.1%, *t*(264) = −1.26, *p* = 0.209	Asian: 9.4%, Other: 11.1%, *t*(286) = 1.31, *p* = 0.190
NSDS	*r* = 0.04, *p* = 0.437	*r* = −0.01, *p* = 0.897	Gender	M: 17.4%, F: 21.7%, *t*(237) = 1.83, *p* = 0.068	**M: 12.7%, F: 7.4%, *t*(313) = −4.33, *p* < 0.001**
Independent SC	*r* = 0.10, *p* = 0.086	*r* = −0.01, *p* = 0.876	Living situation	With others: 18.2%, Alone: 30.5%, *t*(29) = 2.49, *p* = 0.019	**With others: 10.8%, Alone: 5.6%, *t*(43) = −3.58, *p* < 0.001**
Interdependent SC	*r* = −0.03, *p* = 0.551	*r* = 0.05, *p* = 0.375	Marital status	Unmarried: 20.7%, Married: 15.3%, *t*(210) = −2.44, *p* = 0.016	Unmarried: 11.2%, Married: 8.6%, *t*(201) = −1.90, *p* = 0.059
Age	*r* = −0.05, *p* = 0.378	*r* = 0.12, *p* = 0.034	Education	Some post‐secondary: 19.0%, None: 19.7%, *t*(70) = −0.20, *p* = 0.839	Some post‐secondary: 10.6%, None: 9.4%, *t*(74) = 0.72, *p* = 0.475
Years in country	*r* = 0.00, *p* = 0.966	*r* = 0.07, *p* = 0.229	Income	At/above median: 16.8%, Below: 22.0%, *t*(255) = −2.31, *p* = 0.022	At/above median: 11.1%, Below: 9.6%, *t*(310) = 1.17, *p* = 0.242
Social status	*r* = −0.12, *p* = 0.029	*r* = −0.08, *p* = 0.143			

Bold values indicate statistical significant at *p* < 0.01.

We also examined whether solitude‐related self‐concepts shape participants' recall of how they felt during in‐person social interaction (instead of solitude), in a set of models that parallel our main study models. These analyses are reported in Supplementary Materials C.

## 5 Discussion

Individuals do not always remember their past affective experiences accurately, and this memory‐experience gap may be partially explained by self‐concepts (how individuals view themselves). We discuss the results of our main (registered report) study, which examined how self‐concepts related to solitude and culture may shape how young and middle‐aged adults in the UK and HK recall feeling over a 7‐day ESM period. (The results of our initial pilot study, in which university students recalled their daily affect reported over a 14‐day period, are discussed in Supplementary Materials A). We expected that self‐concepts of introversion, NSDS, and independent SC would be associated with inaccurate recall of overall affect (Hypotheses 1–4), and that SDS and interdependent SC would be associated with inaccurate recall of affect while in solitude (Hypotheses 5–7).

### 5.1 Self‐Concepts Shape the Memory‐Experience Gap for Overall Affect

As hypothesized (H1), individuals retrospectively overestimated their overall levels of HAPA (happiness, energy) and HANA (anxiety, irritation) when recalling how they felt over the 7‐day sampling period, and accurately recalled their LAPA (calm, relaxation) and loneliness (a form of LANA). Findings align with previous research suggesting that people most consistently overestimate their high‐arousal affective states in retrospective reports (e.g., Lay et al. 2017; Mill, Realo, and Allik 2016). Individuals also retrospectively overestimated their overall tiredness, an unexpected finding that was nevertheless in line with previous research showing that individuals recall feeling more fatigued/less energetic at end‐of‐day as compared to mean momentary reports (sample of rheumatology patients; Broderick et al. 2009).

Turning to self‐concepts, more‐introverted individuals retrospectively exaggerated their overall LAPA as hypothesized, but the expected links with HAPA, HANA, and LANA report discrepancies were not supported (H2). This runs counter to previous work (based on Western samples) linking extraversion‐introversion with biassed recall of positive and negative states of both high‐ and low‐arousal (Barrett 1997; Lay et al. 2017; Mill, Realo, and Allik 2016). It may be that introversion only shapes recall of certain affective states over periods longer than the 7‐day retrospection period used in the present study. For example, only one previous study (Mill, Realo, and Allik 2016) linked introversion with exaggerated recall of negative affect (fear and sadness), and these effects only emerged over a 1‐month retrospection period. Moreover, these previous studies have used different affect items in different combinations, leading to conceptual fuzziness that impedes our ability to draw firm conclusions regarding which affective states or dimensions are recalled inaccurately across the literature (Weidman, Steckler, and Tracy 2017). Hence, findings need to be confirmed in future research using a consistent set of affect items. However, our finding that there is a specific link between introversion and retrospective overestimation of LAPA aligns with previous research showing introversion mediates cultural differences in preference for LAPA but not HAPA (Tsai, Knutson, and Fung 2006). If calm and relaxed are more salient affective states for introverted people in our cross‐cultural sample, it may explain their exaggerated LAPA recall. A similar mechanism may explain the unexpected link between interdependent SC and exaggerated recall of feeling “relaxed.” Individuals with higher interdependent SC tend to prioritize group harmony and adjustment, which are facilitated by LAPA states such as relaxation (Markus and Kitayama 1991; Tsai, Knutson, and Fung 2006); hence, they may more readily recall such affective states. However, further work is needed to clarify which affective states are relevant for interdependent SC as our reported association was significant for “relaxed” but not for “calm.”

Hypotheses linking NSDS (H3) and independent SC (H4) with retrospective report discrepancies (Lay et al. 2017; Thomas and Azmitia 2019) were not supported; we found no associations between NSDS and negative affect reports, nor between independent SC and HAPA reports. As a motivation for being in solitude, NSDS may be more relevant to recall of affect in solitude than recall of overall affect; this suggestion is supported by associations of SDS and NSDS with solitude recall reported below. Our study's ability to detect significant associations with independent SC may have been limited by the measure's relatively low internal consistency (*α* = 0.61) compared with our other self‐concept measures. Moreover, previous research on affect recall has not measured independent and interdependent SC directly, but has instead found cultural differences based on ethnicity (e.g., European American vs. Asian American; Oishi 2002); our findings may differ from previous research as ethnicity is an imprecise indicator of independent vs. interdependent SC, and self‐construals may also have shifted in recent years due to sociohistorical changes in the UK and HK (Lay et al. 2020).

### 5.2 Self‐Concepts Shape the Memory‐Experience Gap for Affect in Solitude

Our main aim was to examine how individuals recall feeling in solitude. As expected (H5), participants retrospectively overestimated how calm, relaxed, and lonely they felt in solitude, and underestimated how irritated they felt, reflecting solitude's established affect deactivation effect (Nguyen, Ryan, and Deci 2018; Pauly et al. 2017). Counter to hypotheses, however, participants also retrospectively overestimated how energized they felt, and underestimated how tired they felt, in solitude. Recalling solitude as being more energizing than it actually was may be due to the perception that time to oneself is rejuvenating and replenishes energy for later activities (Larson 1990; Nguyen, Weinstein, and Ryan 2021). Overall, memories of solitude seem to come with a mix of positive and negative states of high and low arousal, affirming the need to examine individual differences (i.e., self‐concepts) that may shape affect recall biases.

Generally in line with hypotheses (H6), higher SDS was associated with recalling solitude more positively in retrospective compared with momentary reports—associations were found for all 8 affective states except for tiredness. Participants higher in NSDS also retrospectively overreported their loneliness in solitude. Findings align with these distinct motivations for solitude, suggesting individuals misremember solitude in ways that reinforce views of themselves as solitude‐seekers by choice (SDS) versus being forced into solitude (NSDS; Lay et al. 2019; Thomas and Azmitia 2019).

The link between SDS motivation and recalling solitude more positively aligns with conceptualizations of solitude as a space for needed reprieve, privacy, and contemplation (e.g., Larson 1990; Long and Averill 2003). The importance of SDS is now established in the literature; both quantitative and qualitative studies asking participants to recollect previous solitude experiences suggest that autonomy and choice are key to remembering solitude positively (e.g., Long and Averill 2003; Weinstein, Nguyen, and Hansen 2021). NSDS motivation, in contrast, may reflect social anxiety or perceived social rejection (e.g., “I don't feel liked when I'm with others”; Thomas and Azmitia 2019), which are in turn strongly linked with loneliness (Hawkley and Cacioppo 2010; Ren, Wesselmann, and van Beest 2021). This may explain why NSDS is associated specifically with recalling solitude as being more lonely. Notably, despite loneliness and tiredness both being classed as LANA states, self‐concepts shaped recall of loneliness, but not tiredness, in solitude, a further testament to the central role of loneliness in individuals' understanding of their experiences of solitude.

Introversion was positively correlated with NSDS, but showed no links with how individuals recalled their solitude experiences in regression models. Hence, as expected, introversion was less consistently linked with solitude recall than were the more specific self‐concepts of SDS and NSDS.

Counter to hypotheses (H7), individuals with higher interdependent SC were more prone to retrospectively overestimate how energized and how lonely they felt in solitude (rather than overreporting LAPA and underreporting HANA in solitude, as would be expected if they enjoyed solitude more for its positive, deactivating effects; Nguyen, Ryan, and Deci 2018; van Zyl, Dankaert, and Guse 2018). Notably, individuals with high interdependent SC also overestimated their HAPA when recalling times of in‐person social interaction (Supplementary Materials C). It may be that those high in interdependent SC remember solitude as a non‐normative situation that feels lonely due to communal cultural norms but also energizing due to relief from social expectations (Liu et al. 2015; Long and Averill 2003). Indeed, individuals with high (vs. low) interdependent SC are more sensitive to social expectations and obligations (Markus and Kitayama 1991; Kitayama, Markus, and Kurokawa 2000). Our overall pattern of findings linking interdependent SC with more inaccurate recall of solitude (and of social interaction) also aligns with the context‐dependent nature of this self‐concept (English and Chen 2007). Specifically, because individuals with high interdependent SC tend to define themselves in relation to social roles and situations, they may be more likely to misremember specific social situations in a way that aligns with expected behaviors or experiences in these situations (e.g., social interaction as pleasurable; solitude as energizing but lonely; Oishi et al. 2004). We must also consider the potential role of individual differences within our sample; for example, among highly interdependent individuals, some may think of solitude as being energizing, and others may think of solitude as being lonely, leading to mixed biases in affect recall when taken together. Further research is needed to test this possibility.

### 5.3 Cultural Considerations in the Experience‐Memory Gap

Findings regarding cultural self‐construals must be interpreted in light of our cross‐cultural sample's characteristics. Counter to prevailing findings that East Asian individuals are more interdependent and Western individuals more independent (e.g., Markus and Kitayama 1991), levels of interdependent SC were higher among our UK (vs. HK) participants, and levels of independent SC were higher among our HK (vs. UK) participants and among our Asian (vs. Non‐Asian) participants. In previous work using multidimensional SC measures, British university students were shown to be highly interdependent on 2 of 7 dimensions (connectedness, commitment to others), and both British and Chinese students scored near the midpoint on the self‐reliance dimension (Yang and Vignoles 2020). Moreover, in the 2020 Gallup World Poll, individuals in HK (vs. the UK) scored higher on measures of self‐orientation and self‐care, and lower on measures of other‐orientation and caring for (non‐kin) others (Lomas et al. 2023). Although such findings do not fully negate prevailing ideas about cultural differences, they reaffirm that differences in independent and interdependent SC cannot be assumed from individuals' ethnicities or locations of residence.

In our study, HK (vs. UK) and Asian (vs. non‐Asian) individuals were more prone to retrospectively overestimate their happiness overall (across social situations) and to underestimate their tiredness in solitude. Notably, although they did not differ in overall time spent in solitude, individuals in HK spent more time in solitude with others present, whereas those in the UK spent more time in solitude alone (no one present). Individuals across both samples reported feeling less calm, less relaxed, and less lonely at moments of solitude with others compared with solitude alone. If solitude with others is more stimulating than complete aloneness (McGonigal 2011), those who more often spend solitude with others (i.e., those in HK) may retrospectively underestimate how tired they felt. Notably, HK (vs. UK) individuals were also more prone to underestimate their irritation and their tiredness when recalling in‐person social interaction (Supplementary Materials C, Tables SM27 and SM29), in line with interdependent cultural norms (Markus and Kitayama 1991).

Acculturation processes must also be considered as over a quarter of our study participants were immigrants. Those who had lived more years in their current country were more prone to retrospectively underestimate their loneliness in solitude. This suggests that greater integration within one's host culture may help individuals emotionally cope with solitude, regardless of their cultural background and current residence (Lay et al. 2020).

### 5.4 Limitations and Future Directions

A key strength of the present study was to assess affect recall accuracy directly by comparing retrospective reports to concurrent affect reports captured during the period of retrospection. However, participants did not always provide retrospective reports immediately after the 1‐week ESM period; reporting delays may have shaped our findings given that influences of self‐concepts (semantic memory) on affect recall have been shown to grow with time (Lay et al. 2017; Robinson and Clore 2002). Although our models did control for time elapsed between ESM and retrospection, future work should examine how solitude experience recall may change over different retrospection periods (e.g., 1 week vs. 1 month).

Moreover, our study participants reported their affect over 7 consecutive days, including weekdays and weekends, but analyses made no day of the week distinctions. Previous ESM research has found that on weekends (vs. weekdays) HAPA during social interaction is higher, as is overall affective well‐being (e.g., de Vries, Baselmans, and Bartels 2021; White et al. 2022); hence, future work on affect retrospection should also account for weekday/weekend effects.

As mentioned, our study also revealed unexpected positive associations between East Asian cultural background and independent (rather than interdependent) SC. Future research examining cultural influences on affect retrospection and solitude should include additional cultures that vary in level and type of individualism and collectivism to account for a fuller range of participant self‐construals (Lomas et al. 2023). Moreover, as the present study is limited by its correlational design, future work should manipulate self‐concepts (e.g., by priming independent/interdependent SC; Yang and Vignoles 2020) to establish causal relationships with retrospective affect report biases and solitude recall.

Finally, our study of participants aged 18–49 revealed that older (vs. younger) individuals were more prone to retrospectively underestimate their tiredness in solitude, in line with previous research indicating that negativity biases in memory decreases with age (e.g., Lay et al. 2017; Mill, Realo, and Allik 2016). As affective experiences in solitude have also been shown to improve from young to middle adulthood, and even more so in old age (potentially due to enhanced emotion regulation skills; Pauly et al. 2017), future research examining affect and solitude recall should include participants age 50+ to better understand how these processes unfold in the second half of life.

### 5.5 Implications and Conclusion

This study is the first to bridge the experience‐memory gap literature and the solitude literature. Findings align with the accessibility model of emotional self‐report (Robinson and Clore 2002) and extend it to situations of solitude by suggesting that self‐concepts help guide recall of how one feels in solitude when information from current experience is no longer accessible. The information provided by self‐concepts is a double‐edged sword. While on the one hand, self‐concepts reflect useful self‐knowledge built over a lifetime, on the other hand, self‐concepts may also reflect biases in how one views oneself. We identify specific self‐concepts (SDS, NSDS, and interdependent SC) that may distort recall of solitude experiences. Hence, our findings point to potential pitfalls (inaccuracies) of relying on retrospective reports to assess how individuals feel in solitude, and underscore the utility of ESM for capturing lived experiences of solitude (e.g., Larson 1990; Schwarz 2007).

If we consider implications for well‐being, to what extent is biassed affect recall adaptive (healthy) versus maladaptive (unhealthy)? In cases when individuals recall solitude more positively than they actually experience it in the moment, this may suggest that they have benefited from engaging in solitary problem‐solving or self‐reflection that feels difficult at the time but that ultimately results in personal growth and well‐being (Lay et al. 2019; Levine, Lench, and Safer 2009; Long and Averill 2003). Moreover, such positive biases in recall may make people more resilient to adversity (Colombo et al. 2020), including the challenges of self‐isolation. Future research should pay attention to potential discrepancies between immediate experiences of solitude and longer‐term regulatory benefits of solitude, given that individuals may report low enjoyment during solitude but nevertheless benefit from taking time to themselves (e.g., Lay et al. 2019; Nguyen, Weinstein, and Deci 2022). Moreover, recalling solitude more negatively than it is actually experienced may also be useful if it discourages individuals from engaging in unhealthy self‐isolating behaviors (Levine, Lench, and Safer 2009). However, such negative expectations may also prevent individuals from pursuing solitude's potential benefits (Ratner and Hamilton 2015). Future research can explore these possibilities to examine the adaptiveness of affect recall in solitude.

## Author Contributions

The authors take full responsibility for this article.

## Ethics Statement

This study was approved by the Psychology Research Ethics Committee at the University of Exeter, the Psychological Sciences and Health Department Ethics Committee at the University of Strathclyde, the Faculty Human Research Ethics Committee at The Education University of Hong Kong, and the Research Ethics Committee at Lingnan University.

## Conflicts of Interest

The authors have no conflicts of interest to declare.

## Supporting information


**Table S1.** Pilot study: Regression models predicting differences between retrospective and mean daily reports of overall affective states across situations.
**Table S2.** Pilot study: Multilevel models predicting daily affective states from daily hours spent in solitude (*N* = 104 individuals, *n* = 1370 assessments).
**Table S3.** Pilot study: Regression models predicting retrospective solitude‐positive affect and solitude‐negative affect slopes.
**Table S4.** Main study sample descriptive statistics and bivariate correlations for variables used in planned confirmatory analyses.
**Table S5.** Model predicting discrepancy between retrospective and mean momentary reports of overall happiness.
**Table S6.** Model predicting discrepancy between retrospective and mean momentary reports of overall energy.
**Table S7.** Model predicting discrepancy between retrospective and mean momentary reports of overall calm.
**Table S8.** Model predicting discrepancy between retrospective and mean momentary reports of overall relaxation.
**Table S9.** Model predicting discrepancy between retrospective and mean momentary reports of overall irritation.
**Table S10.** Model predicting discrepancy between retrospective and mean momentary reports of overall anxiety.
**Table S11.** Model predicting discrepancy between retrospective and mean momentary reports of overall loneliness.
**Table S12.** Model predicting discrepancy between retrospective and mean momentary reports of overall tiredness.
**Table S13.** Model predicting discrepancy between retrospective and mean momentary reports of happiness in solitude (no in‐person or virtual interaction).
**Table S14.** Model predicting discrepancy between retrospective and mean momentary reports of energy in solitude (no in‐person or virtual interaction).
**Table S15.** Model predicting discrepancy between retrospective and mean momentary reports of calm in solitude (no in‐person or virtual interaction).
**Table S16.** Model predicting discrepancy between retrospective and mean momentary reports of relaxation in solitude (no in‐person or virtual interaction).
**Table S17.** Model predicting discrepancy between retrospective and mean momentary reports of irritation in solitude (no in‐person or virtual interaction).
**Table S18.** Model predicting discrepancy between retrospective and mean momentary reports of anxiety in solitude (no in‐person or virtual interaction).
**Table S19.** Model predicting discrepancy between retrospective and mean momentary reports of loneliness in solitude (no in‐person or virtual interaction).
**Table S20.** Model predicting discrepancy between retrospective and mean momentary reports of tiredness in solitude (no in‐person or virtual interaction).
**Table S21.** Retrospective report discrepancies (retrospective report minus mean of momentary reports) during in‐person social interaction for eight affective states.
**Table S22.** Model predicting discrepancy between retrospective and mean momentary reports of happiness during social interaction (in‐person interaction).
**Table S23.** Model predicting discrepancy between retrospective and mean momentary reports of feeling energised during social interaction (in‐person interaction).
**Table S24.** Model predicting discrepancy between retrospective and mean momentary reports of calm during social interaction (in‐person interaction).
**Table S25.** Model predicting discrepancy between retrospective and mean momentary reports of relaxation during social interaction (in‐person interaction).
**Table S26.** Model predicting discrepancy between retrospective and mean momentary reports of irritation during social interaction (in‐person interaction).
**Table S27.** Model predicting discrepancy between retrospective and mean momentary reports of anxiety during social interaction (in‐person interaction).
**Table S28.** Model predicting discrepancy between retrospective and mean momentary reports of loneliness during social interaction (in‐person interaction).
**Table S29.** Model predicting discrepancy between retrospective and mean momentary reports of tiredness during social interaction (in‐person interaction).

## References

[jopy12971-bib-0001] Adler, N. , and J. Stewart . 2007. The MacArthur Scale of Subjective Social Status, edited by D. John and T. Catherine . San Francisco, CA: MacArthur Research Network on Socioeconomic Status and Health http://www.macses.ucsf.edu.

[jopy12971-bib-0002] Asendorpf, J. B. , R. Banse , and D. Mücke . 2002. “Double Dissociation Between Implicit and Explicit Personality Self‐Concept: The Case of shy Behavior.” Journal of Personality and Social Psychology 83, no. 2: 380–393. 12150235

[jopy12971-bib-0003] Averill, J. R. , and L. Sundararajan . 2014. “Experiences of Solitude: Issues of Assessment, Theory, and Culture.” In The Handbook of Solitude: Psychological Perspectives on Social Isolation, Social Withdrawal, and Being Alone, edited by R. J. Coplan and J. C. Bowker , 90–108. Malden, MA: John Wiley.

[jopy12971-bib-0004] Barrett, L. F. 1997. “The Relationships Among Momentary Emotion Experiences, Personality Descriptions, and Retrospective Ratings of Emotion.” Personality and Social Psychology Bulletin 23, no. 10: 1100–1110.

[jopy12971-bib-0006] Broderick, J. E. , J. E. Schwartz , S. Schneider , and A. A. Stone . 2009. “Can end‐Of‐Day Reports Replace Momentary Assessment of Pain and Fatigue?” The Journal of Pain 10, no. 3: 274–281. 19070550 10.1016/j.jpain.2008.09.003 PMC2757263

[jopy12971-bib-0007] Brose, A. , U. Lindenberger , and F. Schmiedek . 2013. “Affective States Contribute to Trait Reports of Affective Well‐Being.” Emotion 13, no. 5: 940–948. 23627720 10.1037/a0032401

[jopy12971-bib-0073] Burger, J. M. 1995. “Individual Differences in Preference for Solitude.” Journal of Research in Personality 29, no. 1: 85–108.

[jopy12971-bib-0008] Campbell, S. W. , and M. Q. Ross . 2021. “Re‐Conceptualizing Solitude in the Digital Era: From “Being Alone” to “Noncommunication”.” Communication Theory 32, no. 3: 387–406.

[jopy12971-bib-0010] Choi, I. , and Y. Choi . 2002. “Culture and Self‐Concept Flexibility.” Personality and Social Psychology Bulletin 28, no. 11: 1508–1517.

[jopy12971-bib-0011] Colombo, D. , C. Suso‐Ribera , J. Fernández‐Álvarez , et al. 2020. “Affect Recall Bias: Being Resilient by Distorting Reality.” Cognitive Therapy and Research 44, no. 5: 906–918.

[jopy12971-bib-0012] Conway, M. A. , and C. W. Pleydell‐Pearce . 2000. “The Construction of Autobiographical Memories in the Self‐Memory System.” Psychological Review 107, no. 2: 261–288. 10789197 10.1037/0033-295x.107.2.261

[jopy12971-bib-0013] Costa, P. T. , and R. R. McCrae . 1980. “Influence of Extraversion and Neuroticism on Subjective Well‐Being: Happy and Unhappy People.” Journal of Personality and Social Psychology 38, no. 4: 668–678. 7381680 10.1037//0022-3514.38.4.668

[jopy12971-bib-0014] de Vries, L. P. , B. M. Baselmans , and M. Bartels . 2021. “Smartphone‐Based Ecological Momentary Assessment of Well‐Being: A Systematic Review and Recommendations for Future Studies.” Journal of Happiness Studies 22, no. 5: 2361–2408. 34720691 10.1007/s10902-020-00324-7 PMC8550316

[jopy12971-bib-0015] Duffy, K. A. , E. G. Helzer , R. H. Hoyle , J. Fukukura Helzer , and T. L. Chartrand . 2018. “Pessimistic Expectations and Poorer Experiences: The Role of (Low) extraversion in Anticipated and Experienced Enjoyment of Social Interaction.” PLoS ONE 13, no. 7: e0199146. 29975736 10.1371/journal.pone.0199146 PMC6033406

[jopy12971-bib-0016] English, T. , and S. Chen . 2007. “Culture and Self‐Concept Stability: Consistency Across and Within Contexts Among Asian Americans and European Americans.” Journal of Personality and Social Psychology 93, no. 3: 478–490. 17723061 10.1037/0022-3514.93.3.478

[jopy12971-bib-0017] Epley, N. , and J. Schroeder . 2014. “Mistakenly seeking solitude.” Journal of Experimental Psychology: General 143, no. 5: 1980–1999. 25019381 10.1037/a0037323

[jopy12971-bib-0018] Fredrickson, B. L. 2000. “Extracting Meaning From Past Affective Experiences: The Importance of Peaks, Ends, and Specific Emotions.” Cognition and Emotion 14, no. 4: 577–606.

[jopy12971-bib-0019] Guo, X. , S. J. Schwartz , and B. E. McCabe . 2008. “Aging, Gender, and Self: Dimensionality and Measurement Invariance Analysis on Self‐Construal.” Self and Identity 7, no. 1: 1–24.

[jopy12971-bib-0020] Hatano, A. , C. Ogulmus , H. Shigemasu , and K. Murayama . 2022. Thinking About Thinking: People Underestimate How Enjoyable and Engaging Just Waiting Is. PsyArXiv. 10.1037/xge0001255 35901414

[jopy12971-bib-0021] Hawkley, L. C. , and J. T. Cacioppo . 2010. “Loneliness Matters: A Theoretical and Empirical Review of Consequences and Mechanisms.” Annals of Behavioral Medicine 40, no. 2: 218–227. 20652462 10.1007/s12160-010-9210-8 PMC3874845

[jopy12971-bib-0022] Hong Kong Census and Statistics Department . 2017. 2016 Population by‐Census: Summary Results. 12292262

[jopy12971-bib-0023] Jessup, G. , S. Bian , Y.‐W. R. Chen , and A. Bundy . 2012. P.I.E.L. Survey Application Manual. Sydney, Australia: The University of Sydney.

[jopy12971-bib-0024] Jiang, D. , H. H. Fung , J. C. Lay , M. C. Ashe , P. Graf , and C. A. Hoppmann . 2019. “Everyday Solitude, Affective Experiences, and Well‐Being in old Age: The Role of Culture Versus Immigration.” Aging & Mental Health 23, no. 9: 1095–1104. 30621431 10.1080/13607863.2018.1479836

[jopy12971-bib-0025] John, O. P. , E. M. Donahue , and R. L. Kentle . 1991. The Big Five Inventory–Versions 4a and 54. Berkeley, CA: University of California, Berkeley Institute of Personality and Social Research.

[jopy12971-bib-0026] Kahneman, D. 2000. “Experienced Utility and Objective Happiness: A Moment‐Based Approach.” In Choices, Values, and Frames, edited by D. Kahnemannm and A. Tversky , 673–692. New York: Cambridge University Press.

[jopy12971-bib-0027] Kirtley, O. J. , G. Lafit , R. Achterhof , A. P. Hiekkaranta , and I. Myin‐Germeys . 2021. “Making the Black Box Transparent: A Template and Tutorial for Registration of Studies Using Experience‐Sampling Methods.” Advances in Methods and Practices in Psychological Science 4, no. 1: 251524592092468.

[jopy12971-bib-0028] Kitayama, S. , H. R. Markus , and M. Kurokawa . 2000. “Culture, Emotion, and Well‐Being: Good Feelings in Japan and the United States.” Cognition and Emotion 14, no. 1: 93–124.

[jopy12971-bib-0029] Lai, M. H. C. 2021. “Composite Reliability of Multilevel Data: It's About Observed Scores and Construct Meanings.” Psychological Methods 26, no. 1: 90–102. 32673041 10.1037/met0000287

[jopy12971-bib-0030] Larson, R. W. 1990. “The Solitary Side of Life: An Examination of the Time People Spend Alone From Childhood to old Age.” Developmental Review 10, no. 2: 155–183.

[jopy12971-bib-0031] Lay, J. C. , H. H. Fung , D. Jiang , et al. 2020. “Solitude in Context: On the Role of Culture, Immigration, and Acculturation in the Experience of Time to Oneself.” International Journal of Psychology 55, no. 4: 562–571. 31853988 10.1002/ijop.12641

[jopy12971-bib-0032] Lay, J. C. , D. Gerstorf , S. B. Scott , T. Pauly , and C. A. Hoppmann . 2017. “Neuroticism and Extraversion Magnify Discrepancies Between Retrospective and Concurrent Affect Reports.” Journal of Personality 85, no. 6: 817–829. 27859246 10.1111/jopy.12290

[jopy12971-bib-0033] Lay, J. C. , T. Pauly , P. Graf , J. C. Biesanz , and C. A. Hoppmann . 2019. “By Myself and Liking It? Predictors of Distinct Types of Solitude Experiences in Daily Life.” Journal of Personality 87, no. 3: 633–647. 30003553 10.1111/jopy.12421

[jopy12971-bib-0034] Levine, L. J. , H. C. Lench , and M. A. Safer . 2009. “Functions of Remembering and Misremembering Emotion.” Applied Cognitive Psychology 23, no. 8: 1059–1075.

[jopy12971-bib-0035] Liu, J. , X. Chen , R. J. Coplan , X. Ding , L. Zarbatany , and W. Ellis . 2015. “Shyness and Unsociability and Their Relations With Adjustment in Chinese and Canadian Children.” Journal of Cross‐Cultural Psychology 46, no. 3: 371–386.

[jopy12971-bib-0036] Lomas, T. , P. Diego‐Rosell , K. Shiba , et al. 2023. “Complexifying Individualism Versus Collectivism and West Versus East: Exploring Global Diversity in Perspectives on Self and Other in the Gallup World Poll.” Journal of Cross‐Cultural Psychology 54, no. 1: 61–89.

[jopy12971-bib-0037] Long, C. R. , and J. R. Averill . 2003. “Solitude: An Exploration of Benefits of Being Alone.” Journal for the Theory of Social Behaviour 33, no. 1: 21–44.

[jopy12971-bib-0038] Maes, M. , J. M. Wang , W. Van den Noortgate , and L. Goossens . 2016. “Loneliness and Attitudes Toward Being Alone in Belgian and Chinese Adolescents: Examining Measurement Invariance.” Journal of Child and Family Studies 25, no. 5: 1408–1415.

[jopy12971-bib-0039] Markus, H. 1977. “Self‐Schemata and Processing Information About the Self.” Journal of Personality and Social Psychology 35, no. 2: 63–78.

[jopy12971-bib-0040] Markus, H. R. , and S. Kitayama . 1991. “Culture and the Self: Implications for Cognition, Emotion, and Motivation.” Psychological Review 98, no. 2: 224–253.

[jopy12971-bib-0041] McGonigal, J. 2011. Reality Is Broken: Why Games Make Us Better and how They can Change the World. New York: Penguin Press.

[jopy12971-bib-0042] Mill, A. , A. Realo , and J. Allik . 2016. “Retrospective Ratings of Emotions: The Effects of Age, Daily Tiredness, and Personality.” Frontiers in Psychology 6: Article 2020. 26793142 10.3389/fpsyg.2015.02020 PMC4707248

[jopy12971-bib-0043] Neubauer, A. B. , S. B. Scott , M. J. Sliwinski , and J. M. Smyth . 2020. “How Was Your day? Convergence of Aggregated Momentary and Retrospective end‐Of‐Day Affect Ratings Across the Adult Life Span.” Journal of Personality and Social Psychology 119, no. 1: 185–203. 31070397 10.1037/pspp0000248 PMC6842033

[jopy12971-bib-0044] Nguyen, T.‐V. T. , R. M. Ryan , and E. L. Deci . 2018. “Solitude as an Approach to Affective Self‐Regulation.” Personality and Social Psychology Bulletin 44, no. 1: 92–106. 29072531 10.1177/0146167217733073

[jopy12971-bib-0045] Nguyen, T.‐v. T. , N. Weinstein , and E. Deci . 2022. “Alone With our Thoughts: Investigation of Autonomy Supportive Framing as a Driver of Enjoyment During Quiet Time in Solitude.” Collabra: Psychology 8, no. 1: 31629.

[jopy12971-bib-0046] Nguyen, T.‐V. T. , N. Weinstein , and R. M. Ryan . 2021. “The Possibilities of Aloneness and Solitude: Developing an Understanding Framed Through the Lens of Human Motivation and Needs.” In The Handbook of Solitude: Psychological Perspectives on Social Isolation, Social Withdrawal, and Being Alone, edited by R. J. Coplan , J. C. Bowker , and L. J. Nelson , 2nd ed., 224–239. Hoboken, NJ: Wiley.

[jopy12971-bib-0047] Office for National Statistics . (2017). 2011 Census: Aggregate Data [Data set]. Colchester, UK: UK Data Service. 10.5257/census/aggregate-2011-2.

[jopy12971-bib-0048] Oishi, S. 2002. “The Experiencing and Remembering of Well‐Being: A Cross‐Cultural Analysis.” Personality and Social Psychology Bulletin 28, no. 10: 1398–1406.

[jopy12971-bib-0049] Oishi, S. , E. Diener , C. Napa Scollon , and R. Biswas‐Diener . 2004. “Cross‐Situational Consistency of Affective Experiences Across Cultures.” Journal of Personality and Social Psychology 86, no. 3: 460–472. 15008649 10.1037/0022-3514.86.3.460

[jopy12971-bib-0050] Papp, L. M. , P. Pendry , C. D. Simon , and E. K. Adam . 2013. “Spouses' Cortisol Associations and Moderators: Testing Physiological Synchrony and Connectedness in Everyday Life.” Family Process 52, no. 2: 284–298. 23763687 10.1111/j.1545-5300.2012.01413.x PMC3684984

[jopy12971-bib-0051] Pauly, T. , J. C. Lay , U. M. Nater , S. B. Scott , and C. A. Hoppmann . 2017. “How We Experience Being Alone: Age Differences in Affective and Biological Correlates of Momentary Solitude.” Gerontology 63, no. 1: 55–66. 27760422 10.1159/000450608

[jopy12971-bib-0052] Ratner, R. K. , and R. W. Hamilton . 2015. “Inhibited From Bowling Alone.” Journal of Consumer Research 42, no. 2: 266–283.

[jopy12971-bib-0053] Ren, D. , E. D. Wesselmann , and I. van Beest . 2021. “Seeking Solitude After Being Ostracized: A Replication and Beyond.” Personality and Social Psychology Bulletin 47, no. 3: 426–440. 32515281 10.1177/0146167220928238 PMC7897794

[jopy12971-bib-0054] Robinson, M. D. , and G. L. Clore . 2002. “Belief and Feeling: Evidence for an Accessibility Model of Emotional Self‐Report.” Psychological Bulletin 128, no. 6: 934–960. 12405138 10.1037/0033-2909.128.6.934

[jopy12971-bib-0055] Russell, J. A. 1980. “A Circumplex Model of Affect.” Journal of Personality and Social Psychology 39, no. 6: 1161–1178.

[jopy12971-bib-0056] Ryan, R. M. , and E. L. Deci . 2017. Self‐Determination Theory: Basic Psychological Needs in Motivation, Development, and Wellness. New York: Guilford Press.

[jopy12971-bib-0057] Schwarz, N. 2007. “Retrospective and Concurrent Self‐Reports: The Rationale for Real‐Time Data Capture.” In The Science of Real‐Time Data Capture: Self‐Reports in Health Research, edited by A. A. Stone , S. Shiffman , A. A. Atienza , and L. Nebeling , 11–26. Oxford, UK: Oxford University Press.

[jopy12971-bib-0058] Shrout, P. E. , and S. P. Lane . 2012. “Psychometrics.” In Handbook of Research Methods for Studying Daily Life, edited by M. R. Mehl and T. S. Conner , 302–320. New York: Guilford Press.

[jopy12971-bib-0059] Stavrova, O. , and D. Ren . 2021. “Is More Always Better? Examining the Nonlinear Association of Social Contact Frequency With Physical Health and Longevity.” Social Psychological and Personality Science 12, no. 6: 1058–1070.

[jopy12971-bib-0060] Suh, E. M. 2002. “Culture, Identity Consistency, and Subjective Well‐Being.” Journal of Personality and Social Psychology 83, no. 6: 1378–1391. 12500819 10.1037//0022-3514.83.6.1378

[jopy12971-bib-0061] Thomas, V. , and M. Azmitia . 2019. “Motivation Matters: Development and Validation of the Motivation for Solitude Scale–Short Form (MSS‐SF).” Journal of Adolescence 70: 33–42. 30472399 10.1016/j.adolescence.2018.11.004

[jopy12971-bib-0062] Triandis, H. C. , and M. J. Gelfland . 1998. “Converging Measurement of Horizontal and Vertical Individualism and Collectivism.” Journal of Personality and Social Psychology 74, no. 1: 118–128.

[jopy12971-bib-0063] Tsai, J. L. , B. Knutson , and H. H. Fung . 2006. “Cultural Variation in Affect Valuation.” Journal of Personality and Social Psychology 90, no. 2: 288–307. 16536652 10.1037/0022-3514.90.2.288

[jopy12971-bib-0064] van Buuren, S. , and K. Groothuis‐Oudshoorn . 2011. “Mice: Multivariate Imputation by Chained Equations in R.” Journal of Statistical Software 45, no. 3: 1–67.

[jopy12971-bib-0065] van Zyl, C. J. J. , E. Dankaert , and T. Guse . 2018. “Motivation for Solitude: A Cross‐Cultural Examination of Adolescents From Collectivist and Individualist Cultures in South Africa.” Journal of Child and Family Studies 27, no. 3: 697–706.

[jopy12971-bib-0066] Weidman, A. C. , C. M. Steckler , and J. L. Tracy . 2017. “The Jingle and Jangle of Emotion Assessment: Imprecise Measurement, Casual Scale Usage, and Conceptual Fuzziness in Emotion Research.” Emotion 17, no. 2: 267–295. 27642656 10.1037/emo0000226

[jopy12971-bib-0067] Weinstein, N. , H. Hansen , and T. V. Nguyen . 2023. “Definitions of Solitude in Everyday Life.” Personality and Social Psychology Bulletin 49, no. 12: 1663–1678. 36062325 10.1177/01461672221115941 PMC10637102

[jopy12971-bib-0068] Weinstein, N. , T. V. Nguyen , and H. Hansen . 2021. “What Time Alone Offers: Narratives of Solitude From Adolescence to Older Adulthood.” Frontiers in Psychology 12: 714518. 34790144 10.3389/fpsyg.2021.714518 PMC8591032

[jopy12971-bib-0069] White, H. I. , J. C. Bowker , R. E. Adams , and R. J. Coplan . 2022. “Solitude and Affect During Emerging Adulthood: When, and for Whom, Spending Time Alone Is Related to Positive and Negative Affect During Social Interactions.” International Journal of Behavioral Development 46, no. 6: 490–499.

[jopy12971-bib-0070] Yang, S. , and V. L. Vignoles . 2020. “Self‐Construal Priming Reconsidered: Comparing Effects of Two Commonly Used Primes in the UK and China.” Open Science Journal 5, no. 3: 1–32.

[jopy12971-bib-0071] Zelenski, J. M. , K. Sobocko , and D. C. Whelan . 2021. “Introversion, Solitude, and Happiness.” In The Handbook of Solitude: Psychological Perspectives on Social Isolation, Social Withdrawal, and Being Alone, edited by R. J. Coplan , J. C. Bowker , and L. J. Nelson , 2nd ed., 311–324. New York: Wiley‐Blackwell.

[jopy12971-bib-0072] Zelenski, J. M. , D. C. Whelan , L. J. Nealis , C. M. Besner , M. S. Santoro , and J. E. Wynn . 2013. “Personality and Affective Forecasting: Trait Introverts Underpredict the Hedonic Benefits of Acting Extraverted.” Journal of Personality and Social Psychology 104, no. 6: 1092–1108. 23586413 10.1037/a0032281

