# Supplementary material for: Misremembering Solitude: The Role of Personality and Cultural Self‐Concepts in Shaping Discrepancies Between Recalled and Concurrent Affect in Solitude
Source: J Pers. 2024 Aug 16;93(1):174–92. doi: 10.1111/jopy.12971 (PMC11705513; doi:10.1111/jopy.12971)
Supplement: Supplementary file 1 — Table S1. Pilot study: Regression models predicting differences between retrospective and mean daily reports of overall affective states across situations. Table S2. Pilot study: Multilevel models predicting daily affective states from daily hours spent in solitude (N = 104 individuals, n = 1370 assessments). Table S3. Pilot study: Regression models predicting retrospective solitude‐positive affect and solitude‐negative affect slopes. Table S4. Main study sample descriptive statistics and bivariate correlations for variables used in planned confirmatory analyses. Table S5. Model predicting discrepancy between retrospective and mean momentary reports of overall happiness. Table S6. Model predicting discrepancy between retrospective and mean momentary reports of overall energy. Table S7. Model predicting discrepancy between retrospective and mean momentary reports of overall calm. Table S8. Model predicting discrepancy between retrospective and mean momentary reports of overall relaxation. Table S9. Model predicting discrepancy between retrospective and mean momentary reports of overall irritation. Table S10. Model predicting discrepancy between retrospective and mean momentary reports of overall anxiety. Table S11. Model predicting discrepancy between retrospective and mean momentary reports of overall loneliness. Table S12. Model predicting discrepancy between retrospective and mean momentary reports of overall tiredness. Table S13. Model predicting discrepancy between retrospective and mean momentary reports of happiness in solitude (no in‐person or virtual interaction). Table S14. Model predicting discrepancy between retrospective and mean momentary reports of energy in solitude (no in‐person or virtual interaction). Table S15. Model predicting discrepancy between retrospective and mean momentary reports of calm in solitude (no in‐person or virtual interaction). Table S16. Model predicting discrepancy between retrospective and mean momentary reports of relaxation [file JOPY-93-174-s001.docx]

# Supplementary Materials A: Pilot Study

## Pilot study: Method

**Sample.** We recruited 190 UK university students through online adverts and a research subject pool. Participants who completed at least 5 of the 14 daily questionnaires (*N* = 104) were included in analyses, providing a total of 1,245 daily assessments (*M* = 13.2 per participant, range = 5-21). The sample were aged 18-45 (*M* = 19.9); 81.7% female; 78.9% White, 10.6% East/Southeast Asian, 10.6% other or mixed ethnicity; and 74.0% had lived mainly in the UK. Most participants (87.5%) were in the UK over the study period, during which a national lockdown was in place due to Covid-19. Most were living with family (54.8%) or with friends/flatmates (34.6%). Participants reported moderately good physical health (*M* = 3.4), mental health (*M* = 2.8), and overall wellbeing (*M* = 3.1) on a 5-point scale.

**Procedure.** Participants first completed an online pre-survey consisting of informed consent and trait measures (e.g., sociodemographics, introversion). Then, for a 14-day period, participants were asked to complete a questionnaire each evening via a smartphone app (*PIEL survey*; Jessup et al., 2012). This questionnaire asked them to reconstruct their day’s activities, hour by hour, and report on their social situations and affective experiences (time-use survey design; Chenu & Lesnard, 2006). Following this 14-day period, participants completed an online post-survey that included trait measures (e.g., motivations for solitude, cultural self-construals) and debriefing. This was completed, on average, 17.4 days since the start of daily assessments (*SD* = 8.2, *range* = 9-73). Participants were reimbursed with their choice of course credits or entry into a ₤20 gift card draw. The study was approved by the university’s Research Ethics Committee.

**Measures.** We describe only the measures used in the present analyses. Additional measures not used in this analysis are listed on the study OSF page: <https://osf.io/v4yca>.

***Daily affect.*** In each evening questionnaire over the 14-day study, participants were first asked about their affective experiences that day, in the form, “How ____ have you felt today?”, on a scale from 0 (“Not at all”) to 100 (“Very much”). Items captured three positive states: *calm* (*M* = 58.0, *SD* = 22.3), *energised* (*M* = 46.3, *SD* = 24.1), *content* (*M* = 59.5, *SD* = 23.0); and three negative states: *tired* (*M* = 52.6, *SD* = 26.1), *stressed* (*M* = 44.7, *SD* = 26.0), *lonely* (*M* = 29.6, *SD* = 25.2). These items included both high-arousal states (e.g. energised, stressed) and low-arousal states (e.g. calm, tired) in line with affect circumplex models (Russell, 1980; Tsai et al., 2006). For each participant, measures of *peak affect* were derived from their maximum affect scores across the 14-day study: *calm* (*M* = 81.5, *SD* = 12.4), *energised* (*M* = 74.4, *SD* = 15.9), *content* (*M* = 82.5, *SD* = 14.0), *tired* (*M* = 84.6, *SD* = 12.2), *stressed* (*M* = 75.9, *SD* = 19.4, *lonely* (*M* = 62.4, *SD* = 25.5). Measures of *recent affect* were derived from each participant’s affect scores on the last study day with available data: *calm* (*M* = 60.8, *SD* = 22.5), *energised* (*M* = 50.5, *SD* = 26.1), *content* (*M* = 60.6, *SD* = 22.8), *tired* (*M* = 51.8, *SD* = 26.5), *stressed* (*M* = 40.1, *SD* = 26.3), *lonely* (*M* = 29.0, *SD* = 25.9).

***Daily solitude.*** In each evening questionnaire, participants were asked to recall their social situations, hour-by-hour, from the time they woke up. For each hour, participants first indicated which in-person situation(s) took up the majority of that hour: (a) “Alone (no one nearby, no one I can see)”, (b) “One or more people nearby who I was NOT interacting with in-person”, (c) “One or more people nearby who I WAS interacting with in-person (e.g. talking, doing an activity together)”. Hours for which only options (a) and/or (b) were selected were coded as *no in-person interaction*. Participants then indicated which virtual social situation(s) took up the majority of that hour: (a) “Video / phone interaction”, (b) “Texting / active social media interaction”, (c) “Passive social media use, i.e. scrolling / looking / liking photos but not posting or interacting”, (d) “None of the above”. Hours for which only options (c) and/or (d) were selected were coded as *no virtual interaction*. For each day, we then computed the number of waking hours when participants were in solitude, defined as no in-person or virtual interaction (*daily solitude hours*; *M* = 4.93, *SD* = 3.53, *Range* = 0-21).

***Retrospective affect.*** In the post-survey, participants were asked to recall their affective experiences over the 14-day study. The first set of items were of the form, “How ____ did you feel at the end of the day, on average?”, on a 0-100 scale. Items were the same as those in the ESM measures: *calm* (*M* = 58.6, *SD* = 20.3), *energised* (*M* = 36.4, *SD* = 21.8), *content* (*M* = 63.3, *SD* = 17.7), *tired* (*M* = 63.9, *SD* = 22.9), *stressed* (*M* = 48.5, *SD* = 23.9), *lonely* (*M* = 33.6, *SD* = 25.5). The next set of items were of the form, “On days when you spent more time than average in solitude, meaning not interacting with anyone in person or online, how ____ did you feel at the end of the day?”. Participants responded on a 5-point scale from -2 “Much less than average” to +2 “Much more than average”, with 0 indicating “About the same as average”. Items captured *retrospective solitude-affect slopes* (relationship between spending more time in solitude and feeling specific affective states, as recalled by participants) for *calm* (*M* = -0.03, *SD* = 0.82)*, energised* (*M* = -0.49, *SD* = 0.85)*, content* (*M* = -0.48, *SD* = 0.92)*, tired* (*M* = 0.44, *SD* = 0.93)*, stressed* (*M* = 0.41, *SD* = 0.83)*, lonely* (*M* = 1.08, *SD* = 0.96)*.*

***Introversion.*** The Big Five Inventory (John et al., 1991) was administered in the pre-survey; this included an 8-item Extraversion-Introversion scale. Participants responded on a 5-point scale and an average was computed, with higher scores indicating greater introversion (*M* = 2.8, *SD* = 0.9, *α* = .90).

***Self-determined and not self-determined motivations for solitude.*** The Motivation for Solitude Scale (MSS; Thomas & Azmitia, 2019) was administered in the post-survey to assess two types of solitude-seeking motivations: *self-determined solitude (SDS;* 8 items, *M* = 2.4, *SD* = 0.6, *α* = .81), and *not-self-determined solitude (NSDS;* 6 items, *M* = 1.67, *SD* = 0.63, *α* = .84), with higher scores indicating higher SDS/NSDS.

***Independent and interdependent self-construal*.** The Cultural Orientation scale (Triandis & Gelfland, 1998) was administered in the post-survey to measure independent and interdependent self-construal (SC). Participants responded to items on a 6-point scale; eight items assessed *independent SC* (*M* = 3.8, *SD* = 0.6, *α* = .62), and eight items assessed *interdependent SC* (*M* = 4.6, *SD* = 0.6, *α* = .72), with higher scores indicating higher independent/interdependent SC.

***Sociodemographics.*** Several sociodemographic variables were assessed in the pre-survey and coded as follows: Age (years), Gender (1 = F, 0 = M or Other), Ethnicity (1 = White European, 0 = Other ethnicity), Living situation (1 = alone, 0 = not alone), Subjective social status (score from 1 to 10 on a ladder scale, with higher scores indicating higher subjective status relative to one’s community or society; Adler & Stewart, 2007).

**Data analytic approach.** To test Hypothesis 1 examining overall affect recall, we used regression models predicting retrospective affect discrepancies (i.e. difference scores, retrospective affect report - mean of daily affect reports) for each of the six affect items (Models 1a-1f). The model intercept (*b_0_*) captures the overall discrepancy between retrospective and mean daily affect reports for the affect item in question; positive numbers indicate that retrospective reports were overestimated compared to daily reports, and negative numbers indicate that retrospective reports were underestimated. Hypotheses 2-4 were tested by adding self-concept predictors (Introversion, SDS, NSDS, Independent SC, and Interdependent SC) to the models predicting retrospective affect discrepancies (Models 2a-2f). We also included the following covariates: peak and recent affect (for the affect item in question), days elapsed between first daily assessment and post-survey, and participant age, gender, ethnicity, subjective social status, and living situation.

To test hypotheses examining affect recall for situations of solitude, the first step was to obtain daily solitude-affect slopes for each participant. To do so, we used multilevel models to predict daily scores for each of the six affect items from daily solitude hours, while accounting for person-level clustering of the data. Using the *lme4* package in R (Bates et al., 2015), we extracted daily solitude-affect slopes for each of the six affect items (*β_1j_* in the model equations below). Models controlled for the person-mean of daily solitude hours, and daily solitude hours were person-mean-centred, in order to measure the effects of within-person deviations from each person’s average hours spent in solitude each day. Model equations are below (same structure for each of the six affect items).

Level 1: *DAILY_CALM_ij_* = *β_0j_* + *β_1j_ DAILY_SOLITUDE_HOURS_ij_* + *e_ij_*

Level 2: *β_0j_* = *γ_00_* + *γ_01_ PERSONMEAN_DAILY_SOL_HRS_j_ + U_0j_* *β_1j_* = *γ_10_ + U_1j_*

We then used the daily solitude-affect slopes as predictors in regression models predicting the corresponding retrospective solitude-affect slopes (e.g. *calm_retro-slope_*)

Hypothesis 5, regarding overall accuracy of affect recall for solitude, could not be tested with this data because retrospective solitude-affect slopes were measured on a different scale (-2, -1, 0, +1, +2) from daily solitude-affect slopes (change in end-of-day affect for a 1-hour increase in solitude time). However, we indirectly tested H6 and H7 by examining whether self-concepts shape retrospective solitude-affect slopes beyond the influence of daily solitude-affect slopes. Regression models predicted retrospective solitude-affect slopes from daily solitude-affect slopes (Models 3a-3f). To produce Models 4a-4f, we added the same set of self-concept predictors and covariates as we had for Models 2a-2f, except that peak and recent affect were replaced with person-mean affect and person-mean daily solitude hours.

## Pilot study: Results

Bivariate correlations (significant at *p* < 0.01) revealed that individuals higher in introversion (compared to those lower in introversion) reported lower overall contentedness in daily reports, *r* = -.29; and lower overall contentedness, *r* = -.32, and energy, *r* = -.35, in retrospective reports. Introversion and NSDS scores were positively correlated, *r* = .51. SDS was not correlated with any affect measures. However, in daily reports, individuals higher in NSDS (compared to those lower in NSDS) reported lower overall calm, *r* = -.34, and contentedness, *r* = -.37; and higher overall stress, *r* = .32, tiredness, *r* = .26, and loneliness, *r* = .30. Moreover, in retrospective reports, high-NSDS individuals reported lower overall calm, *r* = -.29, contentedness, *r* = -.39, and energy, *r* = -.39, and higher overall stress, *r* = .34, and loneliness, *r* = .31. Individuals with higher independent SC (compared to those with lower independent SC) reported less tiredness on high-solitude days, *r* = -.26, and those with higher interdependent SC reported higher stress on high-solitude days, *r* = .26. Higher independent SC was associated with higher SDS, *r* = .38, and higher interdependent SC was associated with lower introversion, *r* = -.30, and lower NSDS, *r* = -.29.

Models predicting overall retrospective affect report discrepancies are reported in Supplementary Materials *Table S1*. We looked first at models with no predictors and found partial support for H1. Specifically, individuals underreported their overall energy (by 9 out of 100 points) in retrospective as compared to mean daily reports (Model 1b, *b* = -9.47, *p* < .001) and overreported their overall contentedness (Model 1c, *b* = 4.05, *p* < .001) and tiredness (Model 1d, *b* = 10.32, *p* < .001). No significant report discrepancies were found for the other affect items. Hence, we found partial support for H1, though sometimes in the opposite direction to what we expected, in that participants retrospectively underreported high-arousal affect (energy) and overreported low-arousal affect (tiredness).

Models that included self-concept predictors and covariates showed no associations between overall retrospective report discrepancies and introversion, hence, H2 was not supported. However, H3 was partially supported; individuals higher in NSDS reported lower overall energy levels in retrospective reports as compared to their mean daily reports (Model 2b, *b* = -8.14, *p* = .038). Turning to H4, individuals higher in independent SC reported higher overall tiredness in retrospective compared to mean daily reports (Model 2d, *b* = 7.38, *p* = .049), though this association was for a different affective state (low-arousal negative affect) than those hypothesised. Additional associations were found for model covariates. Specifically, individuals with higher peak energy levels over the study period were more prone to underreporting their energy levels in retrospective compared to mean daily reports (Model 2b, *b* = -0.38, *p* = .018), and individuals with higher peak tiredness levels were *less* prone to retrospectively overreporting their tiredness (Model 2d, *b* = -0.35, *p* = .033). Greater time elapsed since study start was associated with underreporting of calmness in retrospective reports (Model 2a, *b* = -0.48, *p* = .021), and individuals with higher subjective social status were less prone to overreporting their tiredness (Model 2d, *b* = -2.85, *p* = .048).

*Table S2* reports multilevel models predicting daily affect from daily and person-mean hours in solitude. Individuals reported lower calm and contentedness, and higher stress and loneliness, on days when they spent more hours than average in solitude (slope for solitude-calm *b* = -0.73, *p* = .006; solitude-content *b* = -1.18, *p* < .001; solitude-stressed *b* = 0.90, *p* = .003; solitude-lonely *b* = 1.25, *p* < .001). Moreover, individuals who spent more hours overall in solitude reported lower daily contentedness and higher daily loneliness on average (content *b* = -1.85, *p* < .001; lonely *b* = 1.68, *p* = .004).

Models predicting retrospective solitude-affect slopes from daily solitude-affect slopes, self-concept predictors, and covariates are reported in *Table S3*. As mentioned, H5 could not be tested because retrospective solitude-affect slopes were measured on a different scale from daily solitude-affect slopes. No significant associations between SDS and retrospective solitude-affect slopes were found, hence, H6 was not supported. However, individuals higher in introversion reported significantly lower stress on high-solitude days (Model 4e *b* = -0.26, *p* = .020). H7 was not supported, as retrospective solitude-affect slopes showed no significant associations with interdependent SC. However, individuals higher in independent SC retrospectively reported lower loneliness on high-solitude days (Model 4f *b* = -0.41, *p* = .035). Finally, individuals who reported higher daily levels of stress or loneliness on average over the study also retrospectively reported feeling more of these affective states on high-solitude days (stress: Model 4e *b* = 0.17, *p* = .002; loneliness: Model 4f *b* = 0.17, *p* = .008).

## Pilot study: Discussion

Pilot results pertaining to overall affect recall (*Table S1*, Models 1a-1f) and daily solitude-affect slopes (*Table S2*) suggest that participants’ affect reports may have been shaped by their pandemic lockdown situation. Specifically, rather than retrospectively overreporting high-arousal affective states (in line with H1), participants underreported their overall levels of energy and overreported their tiredness and contentedness in retrospective as compared to mean daily reports, perhaps reflecting that they recalled being under-stimulated during lockdown. Moreover, individuals felt worse (more stressed and lonely; less calm and content) on high-solitude days, consistent with previous daily life research (e.g. Larson, 1990), but the expected affect deactivation effect was not seen. This may reflect that participants’ solitude during lockdown was more likely to be involuntary, and hence less likely to bring calm or other noted affective benefits (Long & Averill, 2003).

This pilot also provided initial evidence linking self-concepts pertaining to solitude motivation with affect recall. Specifically, in partial support of H3, individuals higher in NSDS (a trait associated with negative affectivity; Thomas & Azmitia, 2019) recalled feeling less energised in retrospective as compared to mean daily reports. Interestingly, individuals higher in independent SC retrospectively overreported their tiredness levels, an association that was not hypothesised in H4 but that is in line with the idea that individuals high in this trait are motivated to be consistent (e.g., in their affect recall) across social contexts (English & Chen, 2007). Both NSDS and independent SC seem to be linked with an exaggerated sense of deactivation or low vitality. These self-concepts both contain elements of self-distancing from others, and it may be that, particularly for the pilot study participants under pandemic lockdown, seeing oneself as socially distanced may magnify a felt lack of vitality.

Regarding affect recall for solitude, the data showed that individuals higher in introversion recalled feeling lower stress on high-solitude days; this finding was not hypothesised but is in line with the idea that individuals with an introverted self-concept see themselves as enjoying solitude (Zelenski et al., 2013). Interdependent SC did not show the expected effects on retrospective reports of high-solitude days (H7), and moreover, individuals with higher *independent* SC recalled feeling less lonely on high-solitude days, counter to previous research in non-UK cultures linking high independent SC with less positive attitudes towards solitude (Maes et al., 2016; Van Zyl et al., 2018).

Certain aspects of the pilot study design limited our ability to detect biases in affect recall (and how self-concepts may shape these). First, the daily affect measures involved some retrospection (“how ____ have you felt today?”); although daily summaries overlap to some extent with mean momentary affect reports collected throughout a day, they are subject to memory biases (e.g., Neubauer et al., 2020). Hence, analyses could not fully disentangle retrospective from concurrent affect reports, thereby limiting the power to detect retrospective-concurrent discrepancies. Second, the daily reports necessitated the use of summary measures (daily solitude-affect slopes) rather than capturing affective experiences as solitude occurred. Hence, we could not directly assess the accuracy with which individuals recalled how they felt in solitude. Our main study, described below, enabled direct hypothesis testing by using momentary instead of daily reports of affective experiences and by capturing solitude-specific affective experiences in both momentary and retrospective reports.

A second limitation of the pilot was its university sample consisting mainly of students who had grown up in the UK. This sample’s low variability in independent and interdependent SC (*SD* = 0.6 for both 6-point scales) may have limited the power to detect these self-concepts’ effects on affect recall. The proposed study involves samples in Hong Kong and UK to maximise variability in independent and interdependent SC and to disentangle these self-concepts from participants’ cultural backgrounds. The proposed age-stratified community sample further increases study generalisability given age differences in affect recall bias across adulthood (e.g., Lay et al., 2017).

Due to its relatively small sample size (*N* = 104), the pilot study may also have been under-powered to detect between-person effects smaller than 0.2; hence, our main study increases the sample size to *N* > 300 to detect effect sizes of 0.1. Finally, as the pilot was conducted in the unusual situation of a pandemic lockdown, we conducted our main study in 2022-23, in locations without a government-mandated lockdown, to increase generalisability.

Table S1

*Pilot study: Regression models predicting differences between retrospective and mean daily reports of overall affective states across situations*

|  | **Models 1a, 2a: Difference between retrospective and mean daily calm (*N* = 104)^a^** | | | | | **Models 1b, 2b: Difference between retrospective and mean daily energy (*N* = 103)** | | | | |
| --- | --- | --- | --- | --- | --- | --- | --- | --- | --- | --- |
|  | ***b*** | ***β*** | ***SE*** | ***p*** | ***95% CI*** | ***b*** | ***β*** | ***SE*** | ***p*** | ***95% CI*** |
| Intercept (empty model, no predictors) | 1.59 | 0.00 | 1.58 | .316 | [-1.54, 4.73] | **-9.47** | **0.00** | **1.97** | **<.001** | **[-13.38, -5.56]** |
| Intercept (full model with predictors) | 1.88 | 0.02 | 1.59 | .239 | [-1.27, 5.04] | **-9.28** | **0.01** | **1.84** | **<.001** | **[-12.93, -5.63]** |
| Introversion | 2.86 | 0.15 | 2.28 | .212 | [-1.66, 7.39] | -3.59 | -0.16 | 2.64 | .177 | [-8.84, 1.66] |
| Self-determined solitude | 2.14 | 0.08 | 2.88 | .459 | [-3.58, 7.86] | -0.01 | -0.00 | 3.41 | .998 | [-6.78, 6.76] |
| Not-self-determined solitude | -5.05 | -0.20 | 3.36 | .137 | [-11.73, 1.63] | **-8.14** | **-0.26** | **3.86** | **.038** | **[-15.81, -0.47]** |
| Independent self-construal | -0.14 | -0.01 | 3.24 | .965 | [-6.57, 6.29] | -2.04 | -0.06 | 3.77 | .591 | [-9.53, 5.46] |
| Interdependent self-construal | 0.58 | 0.02 | 3.01 | .847 | [-5.40, 6.57] | -1.99 | -0.06 | 3.46 | .567 | [-8.86, 4.88] |
| Peak affect during study ^b^ | -0.15 | -0.11 | 0.15 | .332 | [-0.45, 0.15] | **-0.38** | **-0.31** | **0.16** | **.018** | **[-0.70, -0.07]** |
| Recent affect during study | 0.04 | 0.06 | 0.09 | .608 | [-0.13, 0.21] | -0.06 | -0.08 | 0.09 | .502 | [-0.25, 0.12] |
| Days elapsed since study start | **-0.48** | **-0.25** | **0.21** | **.021** | **[-0.89, -0.07]** | 0.18 | 0.07 | 0.24 | .444 | [-0.29, 0.65] |
| Age (years) | -0.04 | -0.01 | 0.45 | .925 | [-0.94, 0.86] | 1.02 | 0.19 | 0.53 | .058 | [-0.04, 2.07] |
| Gender (1 = Female) | 4.99 | 0.12 | 4.81 | .302 | [-4.57, 14.56] | -3.12 | -0.06 | 5.42 | .566 | [-13.89, 7.65] |
| Ethnicity (1 = European) | 3.51 | 0.09 | 4.57 | .444 | [-5.56, 12.59] | -7.96 | -0.16 | 5.28 | .136 | [-18.46, 2.54] |
| Subjective social status (1-10) | 1.87 | 0.17 | 1.21 | .124 | [-0.52, 4.27] | 1.10 | 0.08 | 1.38 | .428 | [-1.65, 3.85] |
| Living alone (1 = yes) | 11.63 | 0.18 | 7.78 | .139 | [-3.83, 27.09] | -13.29 | -0.17 | 8.97 | .142 | [-31.11, 4.53] |

|  | **Models 1c, 2c: Difference between retrospective and mean daily contentedness (*N* = 104)** | | | | | **Models 1d, 2d: Difference between retrospective and mean daily tiredness (*N* = 103)** | | | | |
| --- | --- | --- | --- | --- | --- | --- | --- | --- | --- | --- |
|  | ***b*** | ***β*** | ***SE*** | ***p*** | ***95% CI*** | ***b*** | ***β*** | ***SE*** | ***p*** | ***95% CI*** |
| Intercept (empty model, no predictors) | **4.05** | **0.00** | **1.17** | **<.001** | **[1.73, 6.37]** | **10.32** | **0.00** | **1.93** | **<.001** | **[6.50, 14.15]** |
| Intercept (full model with predictors) | **4.27** | **0.02** | **1.18** | **<.001** | **[1.91, 6.62]** | **10.36** | **0.00** | **1.86** | **<.001** | **[6.66, 14.05]** |
| Introversion | -0.65 | -0.05 | 1.70 | .702 | [-4.03, 2.72] | -0.54 | -0.02 | 2.62 | .836 | [-5.75, 4.66] |
| Self-determined solitude | 0.32 | 0.02 | 2.22 | .885 | [-4.09, 4.73] | -3.03 | -0.10 | 3.35 | .368 | [-9.69, 3.63] |
| Not-self-determined solitude | -2.81 | -0.15 | 2.53 | .270 | [-7.84, 2.22] | 0.16 | 0.01 | 3.94 | .967 | [-7.67, 8.00] |
| Independent self-construal | -0.68 | -0.03 | 2.47 | .785 | [-5.59, 4.23] | **7.38** | **0.22** | **3.70** | **.049** | **[0.03, 14.72]** |
| Interdependent self-construal | -0.21 | -0.01 | 2.25 | .926 | [-4.67, 4.26] | 4.00 | 0.12 | 3.52 | .258 | [-2.98, 10.99] |
| Peak affect during study ^b^ | -0.15 | -0.17 | 0.11 | .204 | [-0.37, 0.08] | **-0.35** | **-0.22** | **0.16** | **.033** | **[-0.68, -0.03]** |
| Recent affect during study | -0.01 | -0.02 | 0.07 | .852 | [-0.15, 0.12] | -0.00 | -0.00 | 0.08 | .981 | [-0.15, 0.15] |
| Days elapsed since study start | 0.18 | 0.12 | 0.15 | .248 | [-0.13, 0.48] | 0.04 | 0.02 | 0.24 | .861 | [-0.44, 0.52] |
| Age (years) | -0.12 | -0.04 | 0.35 | .731 | [-0.80, 0.57] | -0.98 | -0.19 | 0.54 | .072 | [-2.05, 0.09] |
| Gender (1 = Female) | -0.89 | -0.03 | 3.49 | .799 | [-7.84, 6.05] | 4.78 | 0.09 | 5.41 | .379 | [-5.97, 15.52] |
| Ethnicity (1 = European) | 2.16 | 0.07 | 3.42 | .528 | [-4.62, 8.95] | 6.58 | 0.14 | 5.45 | .230 | [-4.24, 17.41] |
| Subjective social status (1-10) | 0.70 | 0.08 | 0.89 | .435 | [-1.08, 2.48] | **-2.85** | **-0.21** | **1.42** | **.048** | **[-5.68, -0.03]** |
| Living alone (1 = yes) | -1.19 | -0.03 | 5.78 | .837 | [-12.68, 10.29] | -9.03 | -0.12 | 9.22 | .330 | [-27.36, 9.30] |

|  | **Models 1e, 2e: Difference between retrospective and mean daily stress (*N* = 102)** | | | | | **Models 1f, 2f: Difference between retrospective and mean daily loneliness (*N* = 99)** | | | | |
| --- | --- | --- | --- | --- | --- | --- | --- | --- | --- | --- |
|  | ***b*** | ***β*** | ***SE*** | ***p*** | ***95% CI*** | ***b*** | ***β*** | ***SE*** | ***p*** | ***95% CI*** |
| Intercept (empty model, no predictors) | 2.81 | 0.00 | 1.60 | .081 | [-0.36, 5.97] | 2.98 | 0.00 | 1.79 | .099 | [-0.57, 6.53] |
| Intercept (full model with predictors) | 2.55 | -0.02 | 1.64 | .125 | [-0.72, 5.82] | 2.84 | -0.01 | 1.82 | .121 | [-0.77, 6.45] |
| Introversion | 1.27 | 0.07 | 2.39 | .597 | [-3.49, 6.02] | -2.34 | -0.11 | 2.66 | .382 | [-7.63, 2.95] |
| Self-determined solitude | -2.24 | -0.09 | 2.97 | .454 | [-8.15, 3.67] | -1.60 | 0.06 | 3.30 | .629 | [-4.96, 8.15] |
| Not-self-determined solitude | 2.66 | 0.10 | 3.51 | .451 | [-4.32, 9.63] | 5.68 | 0.20 | 3.78 | .137 | [-1.84, 13.19] |
| Independent self-construal | 2.32 | 0.08 | 3.39 | .495 | [-4.41, 9.06] | 0.56 | 0.02 | 3.69 | .880 | [-6.78, 7.89] |
| Interdependent self-construal | -1.82 | -0.07 | 3.12 | .561 | [-8.02, 4.38] | -3.73 | -0.13 | 3.37 | .272 | [-10.44, 2.98] |
| Peak affect during study ^b^ | 0.04 | 0.04 | 0.10 | .715 | [-0.16, 0.23] | 0.13 | 0.18 | 0.09 | .154 | [-0.05, 0.30] |
| Recent affect during study | 0.02 | 0.03 | 0.07 | .772 | [-0.12, 0.16] | -0.08 | -0.11 | 0.09 | .379 | [-0.25, 0.10] |
| Days elapsed since study start | 0.33 | 0.17 | 0.21 | .123 | [-0.09, 0.74] | 0.18 | 0.08 | 0.23 | .445 | [-0.28, 0.63] |
| Age (years) | -0.28 | -0.07 | 0.46 | .546 | [-1.20, 0.64] | -0.27 | -0.06 | 0.51 | .597 | [-1.29, 0.74] |
| Gender (1 = Female) | 0.06 | 0.00 | 4.91 | .990 | [-9.70, 9.82] | -2.34 | -0.05 | 5.42 | .667 | [-13.11, 8.44] |
| Ethnicity (1 = European) | -2.89 | -0.07 | 4.70 | .540 | [-12.22, 6.45] | 0.18 | 0.00 | 5.23 | .973 | [-10.22, 10.58] |
| Subjective social status (1-10) | -1.22 | -0.11 | 1.25 | .333 | [-3.71, 1.27] | -1.18 | -0.09 | 1.36 | .390 | [-3.89, 1.53] |
| Living alone (1 = yes) | -0.22 | -0.00 | 7.96 | .978 | [-16.03, 15.60] | 4.56 | 0.06 | 8.86 | .608 | [-13.07, 22.18] |

^a^ For each model, the dependent variable is the retrospective report minus the mean of the daily reports for that affective state, on a 0-100 scale. Sample size is less than 104 for some models due to missing data for specific affect items.

^b^ Peak affect and recent affect refer to the same affective state as the dependent variable (e.g. peak calm, recent calm)

Table S2

*Pilot study: Multilevel models predicting daily affective states from daily hours spent in solitude (*N *= 104 individuals,* n *= 1370 assessments)*

|  | **Daily calm (ICC = 0.28) ^a^** | | | | | **Daily energy (ICC = 0.32)** | | | | |
| --- | --- | --- | --- | --- | --- | --- | --- | --- | --- | --- |
| **Fixed effects** | ***b*** | ***β*** | ***SE*** | ***p*** | ***95% CI*** | ***b*** | ***β*** | ***SE*** | ***p*** | ***95% CI*** |
| Intercept | **57.29** | **-0.03** | **1.27** | **<.001** | **[54.80, 59.78]** | **45.99** | **-0.01** | **1.45** | **<.001** | **[43.15, 48.82]** |
| Daily hours in solitude ^b^ | **-0.73** | **-0.07** | **0.26** | **.006** | **[-1.25, -0.23]** | -0.38 | -0.03 | 0.26 | .148 | [-0.90, 0.14] |
| Person-mean daily hours in solitude | -0.34 | -0.04 | 0.47 | .474 | [-1.25, 0.06] | -0.49 | -0.06 | 0.53 | .355 | [-1.54, 0.55] |
|  | **Daily contentedness (ICC = 0.35)** | | | | | **Daily tiredness (ICC = 0.27)** | | | | |
| **Fixed effects** | ***b*** | ***β*** | ***SE*** | ***p*** | ***95% CI*** | ***b*** | ***β*** | ***SE*** | ***p*** | ***95% CI*** |
| Intercept | **59.36** | **-0.01** | **1.34** | **<.001** | **[56.73, 61.99]** | **53.11** | **0.02** | **1.49** | **<.001** | **[50.19, 56.03]** |
| Daily hours in solitude | **-1.18** | **-0.11** | **0.25** | **<.001** | **[-1.68, -0.69]** | 0.61 | 0.05 | 0.34 | .074 | [-0.05, 1.30] |
| Person-mean daily hours in solitude | **-1.85** | **-0.22** | **0.49** | **<.001** | **[-2.82, -0.89]** | 0.34 | 0.04 | 0.55 | .533 | [-0.73, 1.42] |
|  | **Daily stress (ICC = 0.38)** | | | | | **Daily loneliness (ICC = 0.38)** | | | | |
| **Fixed effects** | ***b*** | ***β*** | ***SE*** | ***p*** | ***95% CI*** | ***b*** | ***β*** | ***SE*** | ***p*** | ***95% CI*** |
| Intercept | **45.03** | **0.01** | **1.66** | **<.001** | **[41.77, 48.29]** | **29.78** | **0.01** | **1.57** | **<.001** | **[26.70, 32.86]** |
| Daily hours in solitude | **0.90** | **0.08** | **0.29** | **.003** | **[0.33, 1.48]** | **1.25** | **0.11** | **0.31** | **<.001** | **[0.64, 1.86]** |
| Person-mean daily hours in solitude | 0.87 | 0.09 | 0.61 | .159 | [-0.34, 2.08] | **1.68** | **0.18** | **0.58** | **.004** | **[0.54, 2.81]** |

^a^ ICC = intraclass correlation coefficient
^b^ Daily hours in solitude are person-mean centered

Table S3

*Pilot study: Regression models predicting retrospective solitude-positive affect and solitude-negative affect slopes*

|  | **Models 3a, 4a: Retrospective solitude-calm slope (*N* = 98) ^a^** | | | | | **Models 3b, 4b: Retrospective solitude-energised slope (*N* = 98)** | | | | |
| --- | --- | --- | --- | --- | --- | --- | --- | --- | --- | --- |
|  | ***b*** | ***β*** | ***SE*** | ***p*** | ***95% CI*** | ***b*** | ***β*** | ***SE*** | ***p*** | ***95% CI*** |
| Intercept (model without predictors) | -0.18 | 0.00 | 0.52 | .730 | [-1.21, 0.85] | -0.64 | 0.00 | 0.38 | .097 | [-1.39, 0.12] |
| Daily solitude-affect slope ^b^ | -0.20 | -0.03 | 0.70 | .772 | [-1.60, 1.19] | -0.38 | -0.04 | 0.98 | .695 | [-2.32, 1.56] |
| Intercept (model with predictors) | -0.16 | -0.02 | 0.56 | .778 | [-1.27, 0.96] | -2.88 | -0.00 | 7.10 | .686 | [-17.01, 11.25] |
| Daily solitude-affect slope | -0.16 | -0.02 | 0.76 | .836 | [-1.67, 1.36] | -6.28 | -0.65 | 18.69 | .738 | [-43.46, 30.91] |
| Introversion | -0.00 | -0.00 | 0.12 | .993 | [-0.23, 0.23] | 0.09 | 0.10 | 0.13 | .467 | [-0.16, 0.35] |
| Self-determined solitude | 0.21 | 0.16 | 0.15 | .157 | [-0.08, 0.50] | -0.27 | -0.20 | 0.16 | .095 | [-0.60, 0.05] |
| Not-self-determined solitude | 0.15 | 0.11 | 0.18 | .402 | [-0.20, 0.50] | -0.18 | -0.13 | 0.19 | .355 | [-0.56, 0.20] |
| Independent self-construal | 0.14 | 0.10 | 0.17 | .406 | [-0.19, 0.48] | 0.13 | 0.09 | 0.18 | .473 | [-0.23, 0.49] |
| Interdependent self-construal | -0.08 | -0.06 | 0.16 | .632 | [-0.39, 0.24] | 0.02 | 0.02 | 0.17 | .902 | [-0.32, 0.37] |
| Mean affect ^b^ | 0.08 | 0.13 | 0.08 | .267 | [-0.07, 0.23] | 0.34 | 0.59 | 1.12 | .763 | [-1.89, 2.57] |
| Mean daily hours in solitude | -0.02 | -0.07 | 0.03 | .562 | [-0.09, 0.05] | 0.02 | 0.05 | 0.07 | .814 | [-0.12, 0.15] |
| Days elapsed since start | -0.00 | -0.01 | 0.01 | .928 | [-0.02, 0.02] | 0.01 | 0.08 | 0.01 | .499 | [-0.01, 0.03] |
| Age (years) | 0.02 | 0.08 | 0.02 | .459 | [-0.03, 0.07] | 0.02 | 0.07 | 0.03 | .521 | [-0.04, 0.07] |
| Gender (1 = Female) | -0.17 | -0.08 | 0.24 | .469 | [-0.65, 0.30] | -0.12 | -0.05 | 0.26 | .654 | [-0.64, 0.40] |
| Ethnicity (1 = European) | -0.47 | -0.24 | 0.24 | .054 | [-0.96, 0.01] | -0.14 | -0.07 | 0.26 | 595 | [-0.67, 0.39] |
| Subjective social status (1-10) | 0.05 | 0.08 | 0.06 | .476 | [-0.08, 0.17] | 0.04 | 0.07 | 0.07 | .542 | [-0.09, 0.18] |
| Living situation (1 = Alone) | 0.06 | 0.02 | 0.40 | .879 | [-0.74, 0.886] | -0.13 | -0.04 | 0.44 | .775 | [-1.00, 0.74] |

|  | **Models 3c, 4c: Retrospective solitude-content slope (*N* = 99)** | | | | | **Models 3d, 4d: Retrospective solitude-tired slope (*N* = 98)** | | | | |
| --- | --- | --- | --- | --- | --- | --- | --- | --- | --- | --- |
|  | ***b*** | ***β*** | ***SE*** | ***p*** | ***95% CI*** | ***b*** | ***β*** | ***SE*** | ***p*** | ***95% CI*** |
| Intercept (model without predictors) | 0.93 | -0.01 | 0.71 | .194 | [-0.48, 2.35] | **0.43** | **0.00** | **0.13** | **.002** | **[0.17, 0.69]** |
| Daily solitude-affect slope ^b^ | **1.21** | **0.20** | **0.60** | **.048** | **[0.01, 2.40]** | 0.02 | 0.01 | 0.15 | .889 | [-0.28, 0.32] |
| Intercept (model with predictors) | 1.17 | -0.03 | 0.84 | .167 | [-0.50, 2.85] | **0.47** | **0.02** | **0.14** | **.001** | **[0.19, 0.76]** |
| Daily solitude-affect slope | **1.42** | **0.24** | **0.71** | **.049** | **[0.01, 2.84]** | -0.03 | -0.02 | 0.17 | .876 | [-0.37, 0.31] |
| Introversion | 0.11 | 0.11 | 0.13 | .377 | [-0.14, 0.37] | -0.08 | -0.07 | 0.14 | .577 | [-0.35, 0.20] |
| Self-determined solitude | 0.21 | 0.15 | 0.16 | .203 | [-0.12, 0.54] | -0.11 | -0.08 | 0.18 | .528 | [-0.46, 0.24] |
| Not-self-determined solitude | -0.21 | -0.14 | 0.19 | .283 | [-0.60, 0.18] | -0.03 | -0.02 | 0.21 | .897 | [-0.45, 0.39] |
| Independent self-construal | 0.30 | 0.17 | 0.19 | .116 | [-0.08, 0.68] | -0.15 | -0.09 | 0.21 | .473 | [-0.56, 0.26] |
| Interdependent self-construal | -0.22 | -0.08 | -0.15 | .200 | [-0.57, 0.12] | 0.02 | 0.01 | 0.19 | .914 | [-0.36, 0.40] |
| Mean affect ^b^ | 0.10 | 0.10 | 0.16 | .225 | [-0.06, 0.26] | 0.11 | 0.18 | 0.07 | .136 | [-0.04, 0.26] |
| Mean daily hours in solitude | 0.03 | -0.00 | 0.08 | .467 | [-0.05, 0.10] | 0.03 | 0.08 | 0.04 | .494 | [-0.05, 0.11] |
| Days elapsed since start | 0.01 | 0.10 | 0.12 | .244 | [-0.01, 0.04] | -0.00 | -0.00 | 0.01 | .969 | [-0.03, 0.02] |
| Age (years) | 0.01 | 0.10 | 0.02 | .830 | [-0.05, 0.06] | -0.01 | -0.05 | 0.03 | .662 | [-0.07, 0.04] |
| Gender (1 = Female) | 0.02 | -0.06 | 0.01 | .944 | [-0.51, 0.54] | 0.22 | 0.09 | 0.28 | .439 | [-0.34, 0.78] |
| Ethnicity (1 = European) | -0.19 | -0.20 | -0.08 | .494 | [-0.73, 0.35] | -0.12 | -0.05 | 0.29 | .679 | [-0.70, 0.46] |
| Subjective social status (1-10) | -0.03 | 0.06 | -0.04 | .685 | [-0.16, 0.11] | 0.07 | 0.11 | 0.07 | .325 | [-0.07, 0.22] |
| Living situation (1 = Alone) | 0.25 | 0.01 | 0.07 | .571 | [-0.63, 1.14] | -0.07 | -0.02 | 0.47 | .876 | [-1.01, 0.87] |

|  | **Models 3e, 4e: Retrospective solitude-stressed slope (*N* = 97)** | | | | | **Models 3f, 4f: Retrospective solitude-lonely slope (*N* = 98)** | | | | |
| --- | --- | --- | --- | --- | --- | --- | --- | --- | --- | --- |
|  | ***b*** | ***β*** | ***SE*** | ***p*** | ***95% CI*** | ***b*** | ***β*** | ***SE*** | ***p*** | ***95% CI*** |
| Intercept (model without predictors) | 0.08 | 0.01 | 0.26 | .753 | [-0.43, 0.59] | **0.88** | **0.00** | **0.19** | **<.001** | **[0.50, 1.26]** |
| Daily solitude-affect slope ^b^ | 0.37 | 0.14 | 0.27 | .178 | [-0.17, 0.92] | 0.17 | 0.13 | 0.13 | .219 | [-0.10, 0.43] |
| Intercept (model with predictors) | 0.47 | 0.00 | 0.27 | .085 | [-0.07, 1.01] | **0.89** | **0.02** | **0.19** | **<.001** | **[0.51, 1.26]** |
| Daily solitude-affect slope | -0.06 | -0.02 | 0.29 | .842 | [-0.63, 0.51] | 0.17 | 0.13 | 0.13 | .204 | [-0.09, 0.44] |
| Introversion | **-0.26** | **-0.27** | **0.11** | **.020** | **[-0.47, -0.04]** | -0.24 | -0.22 | 0.13 | .059 | [-0.50, 0.01] |
| Self-determined solitude | -0.26 | -0.20 | 0.14 | .062 | [-0.54, 0.01] | -0.05 | -0.03 | 0.16 | .772 | [-0.38, 0.28] |
| Not-self-determined solitude | 0.05 | 0.04 | 0.17 | .762 | [-0.28, 0.38] | 0.09 | 0.06 | 0.19 | .658 | [-0.30, 0.47] |
| Independent self-construal | -0.03 | -0.02 | 0.16 | .861 | [-0.34, 0.28] | **-0.41** | **-0.25** | **0.19** | **.035** | **[-0.79, -0.03]** |
| Interdependent self-construal | 0.23 | 0.17 | 0.15 | .135 | [-0.07, 0.54] | -0.09 | -0.06 | 0.18 | .600 | [-0.44, 0.26] |
| Mean affect ^b^ | **0.17** | **0.34** | **0.05** | **.002** | **[0.06, 0.27]** | **0.17** | **0.29** | **0.06** | **.008** | **[0.05, 0.30]** |
| Mean daily hours in solitude | -0.01 | -0.02 | 0.03 | .872 | [-0.07, 0.06] | 0.00 | 0.01 | 0.04 | .947 | [-0.07, 0.08] |
| Days elapsed since start | -0.01 | -0.09 | 0.01 | .358 | [-0.03, 0.01] | -0.02 | -0.15 | 0.01 | .144 | [-0.04, 0.01] |
| Age (years) | -0.04 | -0.19 | 0.02 | .068 | [-0.09, 0.00] | -0.03 | -0.12 | 0.03 | .257 | [-0.08, 0.02] |
| Gender (1 = Female) | -0.16 | -0.08 | 0.22 | .470 | [-0.61, 0.28] | -0.05 | -0.02 | 0.27 | .863 | [-0.58, 0.49] |
| Ethnicity (1 = European) | 0.32 | 0.16 | 0.24 | .182 | [-0.15, 0.78] | 0.43 | 0.18 | 0.27 | .116 | [-0.11, 0.97] |
| Subjective social status (1-10) | 0.02 | 0.04 | 0.06 | .683 | [-0.09, 0.14] | 0.06 | 0.09 | 0.07 | .371 | [-0.08, 0.20] |
| Living situation (1 = Alone) | 0.45 | 0.14 | 0.38 | .238 | [-0.30, 1.20] | 0.12 | 0.03 | 0.45 | .795 | [-0.78, 1.01] |

^a^ For each model, the dependent variable is the retrospective solitude-affect slope for that affective state (0-100 scale). Sample size is less than 104 for some models due to missing data for specific affect items. All predictors are grand-mean centered except for daily solitude-affect slope.

^b^ Daily solitude-affect slope and mean affect refer to the same affective state as the dependent variable.

# Supplementary Materials B: Main Study Supplementary Tables

Table S4

*Main study sample descriptive statistics and bivariate correlations for variables used in planned confirmatory analyses*

|  | ***M*(*SD*) or *%*** | **1** | **2** | **3** | **4** | **5** | **6** | **7** | **8** | **9** | **10** | **11** | **12** | **13** | **14** | **15** | **16** | **17** | **18** | **19** | **20** | **21** | **22** |
| --- | --- | --- | --- | --- | --- | --- | --- | --- | --- | --- | --- | --- | --- | --- | --- | --- | --- | --- | --- | --- | --- | --- | --- |
| 1. Introversion  (1-5) | 3.18 (0.79) |  |  |  |  |  |  |  |  |  |  |  |  |  |  |  |  |  |  |  |  |  |  |
| 2. SDS (1-5) | 2.72 (0.66) | .07 |  |  |  |  |  |  |  |  |  |  |  |  |  |  |  |  |  |  |  |  |  |
| 3. NSDS (1-5) | 1.90 (0.70) | .37 *** | .21 *** |  |  |  |  |  |  |  |  |  |  |  |  |  |  |  |  |  |  |  |  |
| 4. Independent SC (1-6) | 4.16 (0.61) | .07 | .18 ** | .21 *** |  |  |  |  |  |  |  |  |  |  |  |  |  |  |  |  |  |  |  |
| 5. Interdependent SC (1-6) | 4.31 (0.74) | -.28 *** | .09 | -.15 ** | .12 |  |  |  |  |  |  |  |  |  |  |  |  |  |  |  |  |  |  |
| 6. Retro HAPA discrepancy | 0.28 (1.28) | .02 | .03 | .00 | .06 | .00 |  |  |  |  |  |  |  |  |  |  |  |  |  |  |  |  |  |
| 7. Retro LAPA discrepancy | -0.09 (1.33) | .12 | .00 | -.08 | .04 | .13 | .50 *** |  |  |  |  |  |  |  |  |  |  |  |  |  |  |  |  |
| 8. Retro HANA discrepancy | 0.46 (1.53) | .00 | .03 | .02 | -.07 | -.06 | -.03 | -.13 |  |  |  |  |  |  |  |  |  |  |  |  |  |  |  |
| 9. Retro LANA discrepancy | 0.38 (1.47) | .05 | -.05 | .05 | -.01 | .03 | -.04 | .04 | .54 *** |  |  |  |  |  |  |  |  |  |  |  |  |  |  |
| 10. Peak HAPA (0-10) | 8.45 (1.28) | -.27 *** | .24 *** | -.15 ** | .08 | .29 *** | -.09 | -.09 | .01 | .01 |  |  |  |  |  |  |  |  |  |  |  |  |  |
|  | ***M*(*SD*) or *%*** | **1** | **2** | **3** | **4** | **5** | **6** | **7** | **8** | **9** | **10** | **11** | **12** | **13** | **14** | **15** | **16** | **17** | **18** | **19** | **20** | **21** | **22** |
| 11. Peak LAPA (0-10) | 8.77 (1.14) | -.18 ** | .18 ** | -.18 *** | .10 | .22 *** | .01 | -.03 | .07 | .06 | .76 *** |  |  |  |  |  |  |  |  |  |  |  |  |
| 12. Peak HANA (0-10) | 7.03 (2.31) | .13 | .08 | .33 *** | .12 | -.10 | -.10 | -.11 | .06 | .06 | .13 | .04 |  |  |  |  |  |  |  |  |  |  |  |
| 13. Peak LANA (0-10) | 7.40 (1.85) | .16 ** | -.01 | .36 *** | .19 *** | -.07 | -.06 | -.09 | .03 | .05 | .16 ** | .11 | .69 *** |  |  |  |  |  |  |  |  |  |  |
| 14. Recent HAPA (0-10) | 5.27 (1.99) | -.33 *** | .19 *** | -.28 *** | -.01 | .30 *** | -.03 | .07 | -.17 ** | -.19 *** | .42 *** | .30 *** | -.20 *** | -.22 *** |  |  |  |  |  |  |  |  |  |
| 15. Recent LAPA (0-10) | 5.90 (2.01) | -.26 *** | .12 | -.27 *** | -.03 | .26 *** | .01 | .03 | -.10 | -.19 *** | .37 *** | .40 *** | -.27 *** | -.22 *** | .70 *** |  |  |  |  |  |  |  |  |
| 16. Recent HANA (0-10) | 3.01 (2.04) | .15 ** | -.01 | .36 *** | .10 | -.08 | -.16 ** | -.14 | -.13 | -.04 | -.11 | -.23 *** | .54 *** | .47 *** | -.27 *** | -.40 *** |  |  |  |  |  |  |  |
| 17. Recent LANA (0-10) | 3.86 (1.91) | .24 *** | -.10 | .34 *** | .10 | -.16 ** | -.12 | -.13 | -.11 | -.09 | -.15 ** | -.20 *** | .46 *** | .55 *** | -.37 *** | -.31 *** | .66 *** |  |  |  |  |  |  |
| 18. HAPA discre- pancy for solitude | 0.30 (1.82) | .06 | .13 | .09 | -.04 | .06 | .23 *** | .20 *** | .11 | .05 | -.01 | .01 | .15 ** | .02 | -.10 | -.12 | .07 | .03 |  |  |  |  |  |
| 19. LAPA discre- pancy for solitude | 0.54 (1.97) | .11 | .20 *** | .03 | .00 | -.05 | .19 ** | .27 *** | .18 ** | .14 | .00 | .02 | .12 | .00 | -.10 | -.10 | -.02 | .04 | .65 *** |  |  |  |  |
| 20. HANA discre- pancy for solitude | -0.36 (1.71) | -.07 | -.13 | -.04 | -.01 | .06 | .09 | .01 | .24 *** | .22 *** | .04 | .04 | -.13 | -.03 | .03 | .05 | -.20 *** | -.16 ** | -.30 *** | -.35 *** |  |  |  |
| 21. LANA discre- pancy for solitude | -0.09 (1.94) | -.05 | -.09 | -.01 | -.08 | .15 ** | .02 | -.04 | .18 ** | .42 *** | .12 | .09 | .08 | .07 | .09 | .07 | -.02 | -.05 | -.25 *** | -.18 ** | .44 *** |  |  |
| 22. Peak HAPA solitude (0-10) | 7.09 (1.80) | -.13 | .28 *** | -.15 | .10 | .19 *** | -.04 | .04 | .00 | .11 | .55 *** | .50 *** | .00 | .01 | .38 *** | .38 *** | -.17 ** | -.16 ** | -.25 *** | -.11 | .20 *** | .23 *** |  |
| 23. Peak LAPA solitude (0-10) | 7.88 (1.66) | -.17 ** | .16 ** | -.17 ** | .08 | .14 | .05 | .04 | .04 | .08 | .45 *** | .68 *** | -.10 | -.01 | .27 *** | .44 *** | -.28 *** | -.21 *** | -.13 | -.16 ** | .15 ** | .10 | .69 *** |
|  | ***M*(*SD*) or *%*** | **1** | **2** | **3** | **4** | **5** | **6** | **7** | **8** | **9** | **10** | **11** | **12** | **13** | **14** | **15** | **16** | **17** | **18** | **19** | **20** | **21** | **22** |
| 24. Peak HANA solitude (0-10) | 5.27 (2.61) | .23 *** | .03 | .30 *** | .17 ** | -.08 | -.11 | -.06 | .09 | .17 ** | -.04 | -.10 | .74 *** | .56 *** | -.31 *** | -.33 *** | .57 *** | .50 *** | .18 ** | .16 ** | -.24 *** | .03 | .02 |
| 25. Peak LANA solitude (0-10) | 6.04 (2.19) | .24 *** | -.13 | .34 *** | .25 *** | -.09 | -.02 | .02 | .08 | .17 ** | -.08 | -.07 | .49 *** | .71 *** | -.34 *** | -.30 *** | .43 *** | .56 *** | .09 | .13 | -.07 | -.09 | .01 |
| 26. Recent HAPA solitude (0-10) | 4.93 (2.09) | -.29 *** | .25 *** | -.27 *** | -.05 | .23 *** | .01 | .02 | -.17 ** | -.20 *** | .34 *** | .23 *** | -.23 *** | -.24 *** | .78 *** | .58 *** | -.25 *** | -.35 *** | -.21 *** | -.16 ** | .06 | .14 | .51 *** |
| 27. Recent LAPA solitude (0-10) | 5.85 (2.18) | -.23 *** | .15 | -.22 *** | -.11 | .23 *** | .08 | .04 | -.08 | -.10 | .34 *** | .36 *** | -.31 *** | -.23 *** | .59 *** | .73 *** | -.40 *** | -.34 *** | -.10 | -.18 ** | .11 | .12 | .41 |
| 28. Recent HANA solitude (0-10) | 2.98 (2.23) | .18 ** | -.05 | .27 *** | .10 | -.09 | -.12 | -.17 ** | -.07 | -.02 | -.10 | -.23 *** | .50 *** | .44 *** | -.28 *** | -.36 *** | .83 *** | .58 *** | .04 | .02 | -.27 *** | .03 | -.20 *** |
| 29. Recent LANA solitude (0-10) | 4.02 (1.97) | .23 *** | -.17 ** | .31 *** | .14 | -.19 ** | -.03 | -.08 | .07 | .07 | -.19 ** | -.22 *** | .36 *** | .53 *** | -.41 *** | -.36 *** | .52 *** | .76 *** | .03 | .04 | -.06 | -.10 | -.24 *** |
| 30. Curr. HAPA at retro (0-10) | 5.11 (2.17) | -.27 *** | .15 ** | -.23 *** | .00 | .31 *** | .17 ** | .15 ** | -.08 | -.11 | .39 *** | .33 *** | -.20 *** | -.23 *** | .54 *** | .45 *** | -.19 *** | -.30 *** | .02 | -.04 | .04 | .03 | .33 *** |
| 31. Current LAPA at retro (0-10) | 5.99 (2.34) | -.15 ** | .09 | -.22 *** | .04 | .27 *** | .20 *** | .24 *** | -.12 | -.04 | .31 *** | .37 *** | -.24 *** | -.20 *** | .45 *** | .47 *** | -.29 *** | -.32 *** | -.03 | .03 | .01 | .05 | .29 *** |
| 32. Curr. HANA at retro (0-10) | 2.51 (2.13) | .13 | .05 | .31 *** | .11 | -.13 | -.24 *** | -.25 *** | .20 *** | .13 | -.13 | -.26 *** | .41 *** | .34 *** | -.21 *** | -.26 *** | .54 *** | .41 *** | .03 | -.01 | .03 | .11 | -.12 |
| 33. Curr. LANA at retro (0-10) | 3.70 (2.28) | .15 ** | -.09 | .26 *** | .13 | -.10 | -.11 | -.10 | .16 ** | .30 *** | -.12 | -.19 *** | .32 *** | .42 *** | -.27 *** | -.25 *** | .35 *** | .47 *** | .07 | .07 | .11 | .14 | -.10 |
| 34. Days elapsed ESM-retrospect. | 6.12 (10.41) | .08 | .06 | .09 | -.09 | -.09 | -.01 | -.10 | .08 | .07 | -.01 | .00 | .03 | -.06 | -.07 | -.03 | -.06 | -.06 | .05 | -.01 | -.06 | -.04 | -.08 |
| 35. Proportion assessments solit. | 0.28 (0.22) | .16 ** | -.09 | .05 | .10 | -.01 | .09 | .14 | .02 | .05 | -.31 *** | -.18 ** | -.03 | -.04 | -.21 | -.15 | -.04 | .02 | -.01 | .02 | .06 | .03 | .18 |
| 36. Age (years) | 32.90 (9.05) | .01 | .04 | -.11 | -.12 | .12 | .01 | .00 | -.11 | -.14 | -.12 | -.03 | -.23 *** | -.26 *** | .07 | .11 | -.08 | -.15 ** | .07 | -.03 | -.02 | -.18 ** | -.03 |
|  | ***M*(*SD*) or *%*** | **1** | **2** | **3** | **4** | **5** | **6** | **7** | **8** | **9** | **10** | **11** | **12** | **13** | **14** | **15** | **16** | **17** | **18** | **19** | **20** | **21** | **22** |
| 37. Gender  (1 = male) | 42.9% | -.06 | -.10 | .00 | .17 ** | .07 | -.13 | -.11 | -.13 | -.01 | .09 | .04 | -.11 | -.04 | .23 *** | .21 *** | .01 | .04 | -.10 | -.04 | .03 | -.03 | .16 ** |
| 38. Location (1 = Hong Kong) | 49.8% | .21 *** | .21 *** | .23 *** | .19 *** | -.20 *** | .08 | -.01 | -.08 | -.05 | -.09 | -.02 | -.08 | -.02 | -.21 *** | -.18 ** | -.06 | -.01 | -.02 | -.03 | -.03 | -.26 *** | -.04 |
| 39. Years in current location | 27.89 (12.68) | .04 | -.06 | -.08 | -.02 | .05 | .03 | .02 | -.10 | -.09 | -.08 | -.01 | -.19 ** | -.17 | .04 | .06 | -.09 | -.05 | .05 | -.03 | -.01 | -.20 *** | -.02 |
| 40. Ethnicity  (1 = Asian) | 61.1% | .24 *** | .27 *** | .26 *** | .19 *** | -.13 | .07 | .02 | -.06 | -.03 | -.07 | -.02 | -.07 | -.02 | -.22 *** | -.16 ** | -.03 | -.01 | -.02 | -.03 | -.04 | -.21 *** | -.06 |
| 41. Living alone  (1 = yes) | 8.5% | -.02 | -.02 | .02 | .08 | -.13 | .06 | .05 | -.01 | -.01 | -.04 | -.03 | .00 | .03 | .00 | .07 | .06 | .07 | .09 | .02 | -.02 | .00 | .00 |
| 42. Subj. social status (1-10) | 5.48 (1.62) | -.15 ** | .09 | -.16 ** | -.04 | .08 | .02 | -.04 | -.01 | -.10 | .17 ** | .12 | -.11 | -.13 | .18 *** | .17 ** | -.14 | -.15 ** | -.02 | -.05 | .08 | .12 | .12 |
| 43. Marital status  (1 = married) | 29.2% | -.10 | -.08 | .01 | .07 | -.02 | .04 | .03 | .02 | .00 | .08 | .03 | .06 | .11 | .06 | .00 | -.04 | .01 | .03 | .05 | -.04 | .02 | -.04 |
| 44. Educ. (1 = post-secondary) | 84.0% | .11 | .08 | .09 | .14 | -.01 | -.12 | .00 | .05 | .09 | -.05 | .02 | .06 | .01 | -.09 | -.07 | .10 | .10 | .03 | .07 | -.08 | -.10 | -.05 |
| 45. Household income (1 = at or above median) | 57.9% | .03 | .01 | .05 | -.03 | .02 | .05 | -.02 | .13 | .07 | .01 | -.04 | .02 | -.04 | -.06 | -.09 | -.03 | -.04 | .03 | .07 | -.01 | -.09 | -.12 |

*Note:* ***p* < .01, ****p* < .001

Table S4, continued

|  | **23** | **24** | **25** | **26** | **27** | **28** | **29** | **30** | **31** | **32** | **33** | **34** | **35** | **36** | **37** | **38** | **39** | **40** | **41** | **42** | **43** | **44** |
| --- | --- | --- | --- | --- | --- | --- | --- | --- | --- | --- | --- | --- | --- | --- | --- | --- | --- | --- | --- | --- | --- | --- |
| 24. Peak HANA solitude (0-10) | -.11 |  |  |  |  |  |  |  |  |  |  |  |  |  |  |  |  |  |  |  |  |  |
| 25. Peak LANA solitude (0-10) | -.01 | .64 *** |  |  |  |  |  |  |  |  |  |  |  |  |  |  |  |  |  |  |  |  |
| 26. Recent HAPA solitude (0-10) | .30 *** | -.32 *** | -.38 *** |  |  |  |  |  |  |  |  |  |  |  |  |  |  |  |  |  |  |  |
| 27. Recent LAPA solitude (0-10) | .52 *** | -.39 *** | -.31 *** | .67 *** |  |  |  |  |  |  |  |  |  |  |  |  |  |  |  |  |  |  |
| 28. Recent HANA solitude (0-10) | -.34 *** | .62 *** | .40 *** | -.29 *** | -.47 *** |  |  |  |  |  |  |  |  |  |  |  |  |  |  |  |  |  |
| 29. Recent LANA solitude (0-10) | -.25 *** | .42 *** | .67 *** | -.45 *** | -.37 *** | .56 *** |  |  |  |  |  |  |  |  |  |  |  |  |  |  |  |  |
| 30. Current HAPA at retro (0-10) | .28 *** | -.25 *** | -.28 *** | .50 *** | .42 *** | -.19 ** | -.30 *** |  |  |  |  |  |  |  |  |  |  |  |  |  |  |  |
| 31. Current LAPA at retro (0-10) | .39 *** | -.27 *** | -.19 ** | .34 *** | .43 *** | -.27 *** | -.28 *** | .62 *** |  |  |  |  |  |  |  |  |  |  |  |  |  |  |
| 32. Current HANA at retro (0-10) | -.23 *** | .43 *** | .29 *** | -.17 ** | -.21 *** | .51 *** | .36 *** | -.39 *** | -.54 *** |  |  |  |  |  |  |  |  |  |  |  |  |  |
| 33. Current LANA at retro (0-10) | -.17 ** | .41 *** | .47 *** | -.29 *** | -.25 *** | .37 *** | .50 *** | -.50 *** | -.44 *** | .56 *** |  |  |  |  |  |  |  |  |  |  |  |  |
| 34. Days elapsed ESM-retrospect. | -.01 | -.03 | -.03 | -.07 | -.02 | -.09 | -.11 | -.03 | -.07 | .01 | -.03 |  |  |  |  |  |  |  |  |  |  |  |
| 35. Proportion assessments solit. | .14 | .32 *** | .34 *** | -.16 ** | -.15 | -.02 | .06 | -.13 | -.04 | -.05 | .04 | -.03 |  |  |  |  |  |  |  |  |  |  |

|  | **23** | **24** | **25** | **26** | **27** | **28** | **29** | **30** | **31** | **32** | **33** | **34** | **35** | **36** | **37** | **38** | **39** | **40** | **41** | **42** | **43** | **44** |
| --- | --- | --- | --- | --- | --- | --- | --- | --- | --- | --- | --- | --- | --- | --- | --- | --- | --- | --- | --- | --- | --- | --- |
| 36. Age (years) | .01 | -.14 | -.21 *** | .16 ** | .08 | -.08 | -.22 *** | .16 ** | .14 ** | -.14 | -.19 *** | -.15 ** | .02 |  |  |  |  |  |  |  |  |  |
| 37. Gender  (1 = male) | .07 | -.07 | -.01 | .16 ** | .17 ** | .00 | .03 | .19 *** | .18 ** | .01 | -.02 | -.09 | -.01 | .19 *** |  |  |  |  |  |  |  |  |
| 38. Location (1 = HK) | .00 | -.13 | -.04 | -.13 | -.11 | -.16 ** | -.04 | -.13 | -.12 | -.01 | -.05 | .34 *** | -.03 | .01 | -.08 |  |  |  |  |  |  |  |
| 39. Years in current location | .03 | -.15 | -.10 | .10 | .09 | -.10 | -.08 | .14 | .12 | -.12 | -.10 | -.09 | .04 | .72 *** | .19 *** | .10 |  |  |  |  |  |  |
| 40. Ethnicity  (1 = Asian) | .01 | -.09 | -.03 | -.12 | -.08 | -.11 | -.05 | -.10 | -.07 | .00 | -.05 | .25 *** | .02 | .01 | -.07 | .78 *** | .06 |  |  |  |  |  |
| 41. Living alone  (1 = yes) | .04 | .00 | -.01 | .00 | .07 | -.02 | .04 | .00 | .01 | -.01 | .00 | -.05 | .10 | -.06 | .05 | -.06 | -.05 | .01 |  |  |  |  |
| 42. Subjective social status (1-10) | .07 | -.16 ** | -.19 ** | .21 *** | .20 *** | -.11 | -.09 | .22 *** | .15 ** | -.04 | -.18 ** | -.08 | -.14 | .10 | -.02 | -.08 | .03 | -.02 | -.01 |  |  |  |
| 43. Marital status  (1 = married) | -.13 | -.03 | .01 | -.02 | -.03 | -.01 | .04 | -.05 | -.07 | .06 | .09 | -.09 | -.17 ** | -.28 *** | .01 | -.05 | -.26 *** | -.11 | .03 | -.01 |  |  |
| 44. Educ. (1 = post-secondary) | .01 | .07 | .06 | -.07 | -.05 | .01 | .01 | -.02 | -.07 | .14 | .03 | .10 | .04 | -.04 | .05 | .15 ** | -.01 | .21 *** | .01 | -.09 | -.02 |  |
| 45. Household income (1 = at or above median) | -.17 | -.03 | -.06 | -.05 | -.04 | .01 | -.01 | .04 | -.07 | .06 | -.05 | .06 | -.07 | .04 | -.11 | .08 | .03 | .10 | -.22 *** | .14 | .06 | .12 |

*Note:* ***p* < .01, ****p* < .001

Table S5

*Model predicting discrepancy between retrospective and mean momentary reports of overall happiness*

| **Variable** | ***b*** | ***SE*** | ***β*** | ***p*** | ***95% CI(l)*** | ***95% CI(u)*** |
| --- | --- | --- | --- | --- | --- | --- |
| Intercept | **0.29** | **0.08** | **0.00** | **0.000** | **0.14** | **0.45** |
| Introversion (1-5) | 0.07 | 0.12 | 0.04 | 0.521 | -0.15 | 0.30 |
| Self-determined solitude (1-5) | 0.02 | 0.13 | 0.01 | 0.893 | -0.25 | 0.28 |
| Not-self-determined solitude (1-5) | -0.15 | 0.13 | -0.07 | 0.262 | -0.41 | 0.11 |
| Independent self-construal (1-6) | 0.22 | 0.15 | 0.09 | 0.139 | -0.07 | 0.51 |
| Interdependent self-construal (1-6) | -0.01 | 0.12 | 0.00 | 0.954 | -0.25 | 0.23 |
| Peak happiness (0-10) | **-0.22** | **0.07** | **-0.20** | **0.002** | **-0.37** | **-0.08** |
| Recent happiness (0-10) | 0.04 | 0.05 | 0.06 | 0.411 | -0.06 | 0.14 |
| Current happiness (0-10) | **0.14** | **0.04** | **0.24** | **0.001** | **0.06** | **0.22** |
| Days elapsed since ESM | -0.01 | 0.01 | -0.07 | 0.278 | -0.03 | 0.01 |
| Age (years) | 0.00 | 0.01 | -0.01 | 0.852 | -0.02 | 0.02 |
| Gender (1 = male) | **-0.51** | **0.18** | **-0.17** | **0.004** | **-0.85** | **-0.16** |
| Location (1 = Hong Kong) | **0.56** | **0.19** | **0.19** | **0.004** | **0.18** | **0.93** |
| Living alone (1 = yes) | 0.47 | 0.30 | 0.09 | 0.115 | -0.12 | 1.05 |
| Subjective social status (1-10) | -0.03 | 0.05 | -0.03 | 0.574 | -0.13 | 0.07 |
| Marital status (1 = married) | 0.15 | 0.19 | 0.05 | 0.430 | -0.22 | 0.52 |
| Education (1 = some post-secondary) | -0.42 | 0.22 | -0.10 | 0.063 | -0.86 | 0.02 |
| Income (1 = at or above median) | 0.01 | 0.17 | 0.00 | 0.946 | -0.32 | 0.35 |

Table S6

*Model predicting discrepancy between retrospective and mean momentary reports of overall energy*

| **Variable** | ***b*** | ***SE*** | ***β*** | ***p*** | ***95% CI(l)*** | ***95% CI(u)*** |
| --- | --- | --- | --- | --- | --- | --- |
| Intercept | **0.27** | **0.09** | **0.00** | **0.003** | **0.09** | **0.44** |
| Introversion (1-5) | 0.01 | 0.13 | 0.01 | 0.918 | -0.24 | 0.27 |
| Self-determined solitude (1-5) | 0.13 | 0.15 | 0.05 | 0.413 | -0.18 | 0.43 |
| Not-self-determined solitude (1-5) | 0.04 | 0.15 | 0.02 | 0.794 | -0.25 | 0.32 |
| Independent self-construal (1-6) | 0.14 | 0.16 | 0.05 | 0.391 | -0.18 | 0.45 |
| Interdependent self-construal (1-6) | 0.07 | 0.13 | 0.03 | 0.625 | -0.20 | 0.33 |
| Peak energy (0-10) | -0.16 | 0.07 | -0.15 | 0.022 | -0.30 | -0.02 |
| Recent energy (0-10) | **-0.15** | **0.05** | **-0.20** | **0.003** | **-0.24** | **-0.05** |
| Current energy (0-10) | **0.20** | **0.04** | **0.31** | **0.000** | **0.12** | **0.28** |
| Days elapsed since ESM | 0.00 | 0.01 | 0.01 | 0.832 | -0.02 | 0.02 |
| Age (years) | 0.00 | 0.01 | -0.02 | 0.730 | -0.03 | 0.02 |
| Gender (1 = male) | -0.19 | 0.20 | -0.06 | 0.329 | -0.58 | 0.19 |
| Location (1 = Hong Kong) | -0.06 | 0.21 | -0.02 | 0.789 | -0.46 | 0.35 |
| Living alone (1 = yes) | 0.21 | 0.33 | 0.04 | 0.520 | -0.43 | 0.85 |
| Subjective social status (1-10) | 0.00 | 0.06 | 0.00 | 0.955 | -0.11 | 0.12 |
| Marital status (1 = married) | 0.21 | 0.20 | 0.06 | 0.303 | -0.19 | 0.61 |
| Education (1 = some post-secondary) | **-0.72** | **0.25** | **-0.16** | **0.004** | **-1.21** | **-0.23** |
| Income (1 = at or above median) | 0.24 | 0.19 | 0.07 | 0.211 | -0.13 | 0.61 |

Table S7

*Model predicting discrepancy between retrospective and mean momentary reports of overall calm*

| **Variable** | ***b*** | ***SE*** | ***β*** | ***p*** | ***95% CI(l)*** | ***95% CI(u)*** |
| --- | --- | --- | --- | --- | --- | --- |
| Intercept | -0.09 | 0.08 | 0.00 | 0.275 | -0.25 | 0.07 |
| Introversion (1-5) | **0.31** | **0.12** | **0.16** | **0.009** | **0.08** | **0.54** |
| Self-determined solitude (1-5) | -0.02 | 0.13 | -0.01 | 0.854 | -0.29 | 0.24 |
| Not-self-determined solitude (1-5) | -0.26 | 0.14 | -0.12 | 0.062 | -0.53 | 0.01 |
| Independent self-construal (1-6) | -0.01 | 0.15 | 0.00 | 0.950 | -0.30 | 0.28 |
| Interdependent self-construal (1-6) | 0.22 | 0.12 | 0.11 | 0.073 | -0.02 | 0.46 |
| Peak calm (0-10) | -0.13 | 0.08 | -0.11 | 0.083 | -0.29 | 0.02 |
| Recent calm (0-10) | -0.03 | 0.05 | -0.04 | 0.584 | -0.12 | 0.07 |
| Current calm (0-10) | **0.13** | **0.04** | **0.22** | **0.001** | **0.05** | **0.20** |
| Days elapsed since ESM | -0.02 | 0.01 | -0.11 | 0.067 | -0.03 | 0.00 |
| Age (years) | -0.01 | 0.01 | -0.06 | 0.312 | -0.03 | 0.01 |
| Gender (1 = male) | -0.33 | 0.18 | -0.11 | 0.064 | -0.68 | 0.02 |
| Location (1 = Hong Kong) | 0.22 | 0.19 | 0.07 | 0.260 | -0.16 | 0.59 |
| Living alone (1 = yes) | 0.07 | 0.30 | 0.01 | 0.823 | -0.53 | 0.67 |
| Subjective social status (1-10) | -0.06 | 0.05 | -0.07 | 0.236 | -0.17 | 0.04 |
| Marital status (1 = married) | 0.06 | 0.19 | 0.02 | 0.737 | -0.31 | 0.44 |
| Education (1 = some post-secondary) | 0.21 | 0.23 | 0.05 | 0.364 | -0.24 | 0.66 |
| Income (1 = at or above median) | -0.09 | 0.17 | -0.03 | 0.623 | -0.43 | 0.26 |

Table S8

*Model predicting discrepancy between retrospective and mean momentary reports of overall relaxation*

| **Variable** | ***b*** | ***SE*** | ***β*** | ***p*** | ***95% CI(l)*** | ***95% CI(u)*** |
| --- | --- | --- | --- | --- | --- | --- |
| Intercept | -0.10 | 0.09 | 0.00 | 0.264 | -0.27 | 0.07 |
| Introversion (1-5) | **0.36** | **0.13** | **0.18** | **0.005** | **0.11** | **0.61** |
| Self-determined solitude (1-5) | -0.01 | 0.15 | 0.00 | 0.968 | -0.29 | 0.28 |
| Not-self-determined solitude (1-5) | -0.24 | 0.15 | -0.11 | 0.094 | -0.53 | 0.04 |
| Independent self-construal (1-6) | 0.13 | 0.16 | 0.05 | 0.421 | -0.19 | 0.44 |
| Interdependent self-construal (1-6) | **0.35** | **0.13** | **0.16** | **0.010** | **0.08** | **0.61** |
| Peak relaxation (0-10) | -0.14 | 0.08 | -0.10 | 0.101 | -0.30 | 0.03 |
| Recent relaxation (0-10) | -0.04 | 0.05 | -0.05 | 0.447 | -0.13 | 0.06 |
| Current relaxation (0-10) | **0.16** | **0.04** | **0.26** | **0.000** | **0.08** | **0.24** |
| Days elapsed since ESM | -0.01 | 0.01 | -0.07 | 0.266 | -0.03 | 0.01 |
| Age (years) | 0.00 | 0.01 | 0.01 | 0.836 | -0.02 | 0.02 |
| Gender (1 = male) | -0.48 | 0.19 | -0.15 | 0.013 | -0.86 | -0.10 |
| Location (1 = Hong Kong) | 0.08 | 0.20 | 0.02 | 0.704 | -0.32 | 0.48 |
| Living alone (1 = yes) | 0.65 | 0.33 | 0.11 | 0.048 | 0.00 | 1.29 |
| Subjective social status (1-10) | -0.03 | 0.06 | -0.03 | 0.643 | -0.14 | 0.08 |
| Marital status (1 = married) | 0.26 | 0.20 | 0.07 | 0.198 | -0.14 | 0.66 |
| Education (1 = some post-secondary) | -0.14 | 0.25 | -0.03 | 0.560 | -0.63 | 0.34 |
| Income (1 = at or above median) | 0.06 | 0.19 | 0.02 | 0.747 | -0.31 | 0.43 |

Table S9

*Model predicting discrepancy between retrospective and mean momentary reports of overall irritation*

| **Variable** | ***b*** | ***SE*** | ***β*** | ***p*** | ***95% CI(l)*** | ***95% CI(u)*** |
| --- | --- | --- | --- | --- | --- | --- |
| Intercept | **0.36** | **0.10** | **0.00** | **0.000** | **0.17** | **0.55** |
| Introversion (1-5) | -0.18 | 0.14 | -0.08 | 0.201 | -0.45 | 0.10 |
| Self-determined solitude (1-5) | 0.26 | 0.16 | 0.10 | 0.100 | -0.05 | 0.57 |
| Not-self-determined solitude (1-5) | 0.00 | 0.16 | 0.00 | 0.989 | -0.32 | 0.32 |
| Independent self-construal (1-6) | -0.03 | 0.18 | -0.01 | 0.875 | -0.38 | 0.32 |
| Interdependent self-construal (1-6) | -0.16 | 0.14 | -0.07 | 0.269 | -0.44 | 0.12 |
| Peak irritation (0-10) | 0.03 | 0.04 | 0.04 | 0.550 | -0.06 | 0.11 |
| Recent irritation (0-10) | **-0.20** | **0.06** | **-0.24** | **0.000** | **-0.31** | **-0.09** |
| Current irritation (0-10) | **0.25** | **0.05** | **0.30** | **0.000** | **0.15** | **0.35** |
| Days elapsed since ESM | 0.01 | 0.01 | 0.05 | 0.365 | -0.01 | 0.03 |
| Age (years) | 0.01 | 0.01 | 0.04 | 0.515 | -0.02 | 0.03 |
| Gender (1 = male) | -0.21 | 0.21 | -0.06 | 0.305 | -0.63 | 0.20 |
| Location (1 = Hong Kong) | -0.39 | 0.23 | -0.11 | 0.093 | -0.85 | 0.07 |
| Living alone (1 = yes) | 0.02 | 0.36 | 0.00 | 0.964 | -0.69 | 0.73 |
| Subjective social status (1-10) | -0.12 | 0.06 | -0.11 | 0.047 | -0.25 | 0.00 |
| Marital status (1 = married) | -0.04 | 0.23 | -0.01 | 0.867 | -0.48 | 0.41 |
| Education (1 = some post-secondary) | 0.11 | 0.27 | 0.02 | 0.686 | -0.43 | 0.65 |
| Income (1 = at or above median) | 0.38 | 0.21 | 0.11 | 0.067 | -0.03 | 0.79 |

Table S10

*Model predicting discrepancy between retrospective and mean momentary reports of overall anxiety*

| **Variable** | ***b*** | ***SE*** | ***β*** | ***p*** | ***95% CI(l)*** | ***95% CI(u)*** |
| --- | --- | --- | --- | --- | --- | --- |
| Intercept | **0.55** | **0.10** | **0.00** | **0.000** | **0.35** | **0.74** |
| Introversion (1-5) | 0.10 | 0.14 | 0.04 | 0.503 | -0.19 | 0.38 |
| Self-determined solitude (1-5) | -0.11 | 0.16 | -0.04 | 0.510 | -0.43 | 0.21 |
| Not-self-determined solitude (1-5) | 0.03 | 0.18 | 0.01 | 0.863 | -0.32 | 0.38 |
| Independent self-construal (1-6) | -0.17 | 0.18 | -0.06 | 0.346 | -0.53 | 0.19 |
| Interdependent self-construal (1-6) | -0.04 | 0.15 | -0.02 | 0.767 | -0.34 | 0.25 |
| Peak anxiety (0-10) | 0.07 | 0.05 | 0.10 | 0.163 | -0.03 | 0.18 |
| Recent anxiety (0-10) | **-0.25** | **0.06** | **-0.32** | **0.000** | **-0.36** | **-0.14** |
| Current anxiety (0-10) | **0.23** | **0.05** | **0.33** | **0.000** | **0.14** | **0.31** |
| Days elapsed since ESM | 0.01 | 0.01 | 0.06 | 0.312 | -0.01 | 0.03 |
| Age (years) | -0.03 | 0.01 | -0.13 | 0.036 | -0.05 | 0.00 |
| Gender (1 = male) | -0.37 | 0.21 | -0.10 | 0.086 | -0.79 | 0.05 |
| Location (1 = Hong Kong) | -0.53 | 0.23 | -0.14 | 0.025 | -0.99 | -0.07 |
| Living alone (1 = yes) | 0.26 | 0.37 | 0.04 | 0.484 | -0.47 | 0.99 |
| Subjective social status (1-10) | 0.03 | 0.06 | 0.03 | 0.627 | -0.10 | 0.16 |
| Marital status (1 = married) | -0.22 | 0.23 | -0.05 | 0.335 | -0.68 | 0.23 |
| Education (1 = some post-secondary) | 0.17 | 0.28 | 0.03 | 0.538 | -0.38 | 0.73 |
| Income (1 = at or above median) | 0.29 | 0.21 | 0.08 | 0.171 | -0.13 | 0.71 |

Table S11

*Model predicting discrepancy between retrospective and mean momentary reports of overall loneliness*

| **Variable** | ***b*** | ***SE*** | ***β*** | ***p*** | ***95% CI(l)*** | ***95% CI(u)*** |
| --- | --- | --- | --- | --- | --- | --- |
| Intercept | 0.13 | 0.09 | 0.00 | 0.132 | -0.04 | 0.30 |
| Introversion (1-5) | 0.06 | 0.12 | 0.03 | 0.652 | -0.19 | 0.30 |
| Self-determined solitude (1-5) | -0.30 | 0.14 | -0.12 | 0.038 | -0.57 | -0.02 |
| Not-self-determined solitude (1-5) | 0.17 | 0.15 | 0.07 | 0.261 | -0.13 | 0.46 |
| Independent self-construal (1-6) | -0.09 | 0.16 | -0.03 | 0.583 | -0.40 | 0.23 |
| Interdependent self-construal (1-6) | 0.11 | 0.13 | 0.05 | 0.393 | -0.14 | 0.36 |
| Peak loneliness (0-10) | -0.03 | 0.04 | -0.05 | 0.492 | -0.10 | 0.05 |
| Recent loneliness (0-10) | **-0.15** | **0.05** | **-0.24** | **0.002** | **-0.25** | **-0.06** |
| Current loneliness (0-10) | **0.17** | **0.04** | **0.30** | **0.000** | **0.09** | **0.25** |
| Days elapsed since ESM | 0.01 | 0.01 | 0.08 | 0.168 | -0.01 | 0.03 |
| Age (years) | -0.01 | 0.01 | -0.06 | 0.374 | -0.03 | 0.01 |
| Gender (1 = male) | 0.05 | 0.19 | 0.02 | 0.792 | -0.32 | 0.42 |
| Location (1 = Hong Kong) | -0.31 | 0.20 | -0.10 | 0.127 | -0.71 | 0.09 |
| Living alone (1 = yes) | 0.00 | 0.32 | 0.00 | 0.991 | -0.63 | 0.64 |
| Subjective social status (1-10) | -0.01 | 0.06 | -0.01 | 0.864 | -0.12 | 0.10 |
| Marital status (1 = married) | -0.06 | 0.20 | -0.02 | 0.769 | -0.46 | 0.34 |
| Education (1 = some post-secondary) | 0.38 | 0.24 | 0.09 | 0.124 | -0.10 | 0.85 |
| Income (1 = at or above median) | 0.14 | 0.19 | 0.04 | 0.438 | -0.22 | 0.51 |

Table S12

*Model predicting discrepancy between retrospective and mean momentary reports of overall tiredness*

| **Variable** | ***b*** | ***SE*** | ***β*** | ***p*** | ***95% CI(l)*** | ***95% CI(u)*** |
| --- | --- | --- | --- | --- | --- | --- |
| Intercept | **0.59** | **0.10** | **0.00** | **0.000** | **0.39** | **0.80** |
| Introversion (1-5) | 0.15 | 0.15 | 0.06 | 0.312 | -0.14 | 0.45 |
| Self-determined solitude (1-5) | 0.06 | 0.17 | 0.02 | 0.725 | -0.27 | 0.39 |
| Not-self-determined solitude (1-5) | 0.00 | 0.17 | 0.00 | 0.994 | -0.34 | 0.34 |
| Independent self-construal (1-6) | -0.18 | 0.19 | -0.06 | 0.337 | -0.56 | 0.19 |
| Interdependent self-construal (1-6) | 0.18 | 0.15 | 0.07 | 0.241 | -0.12 | 0.49 |
| Peak tiredness (0-10) | 0.04 | 0.08 | 0.03 | 0.634 | -0.12 | 0.19 |
| Recent tiredness (0-10) | **-0.21** | **0.05** | **-0.26** | **0.000** | **-0.32** | **-0.11** |
| Current tiredness (0-10) | **0.26** | **0.04** | **0.38** | **0.000** | **0.18** | **0.33** |
| Days elapsed since ESM | 0.00 | 0.01 | 0.00 | 0.990 | -0.02 | 0.02 |
| Age (years) | **-0.03** | **0.01** | **-0.16** | **0.009** | **-0.06** | **-0.01** |
| Gender (1 = male) | 0.21 | 0.23 | 0.05 | 0.356 | -0.24 | 0.66 |
| Location (1 = Hong Kong) | 0.00 | 0.24 | 0.00 | 0.991 | -0.48 | 0.48 |
| Living alone (1 = yes) | 0.31 | 0.39 | 0.04 | 0.421 | -0.45 | 1.07 |
| Subjective social status (1-10) | -0.08 | 0.07 | -0.07 | 0.226 | -0.21 | 0.05 |
| Marital status (1 = married) | -0.33 | 0.24 | -0.08 | 0.175 | -0.81 | 0.15 |
| Education (1 = some post-secondary) | 0.38 | 0.29 | 0.07 | 0.196 | -0.20 | 0.96 |
| Income (1 = at or above median) | 0.42 | 0.22 | 0.10 | 0.062 | -0.02 | 0.85 |

Table S13

*Model predicting discrepancy between retrospective and mean momentary reports of happiness in solitude (no in-person or virtual interaction)*

| **Variable** | ***b*** | ***SE*** | ***β*** | ***p*** | ***95% CI(l)*** | ***95% CI(u)*** |
| --- | --- | --- | --- | --- | --- | --- |
| Intercept | 0.21 | 0.12 | 0.00 | 0.070 | -0.02 | 0.44 |
| Introversion (1-5) | 0.00 | 0.17 | 0.00 | 0.995 | -0.34 | 0.34 |
| Self-determined solitude (1-5) | **0.84** | **0.20** | **0.26** | **0.000** | **0.45** | **1.23** |
| Not-self-determined solitude (1-5) | -0.15 | 0.19 | -0.05 | 0.418 | -0.53 | 0.22 |
| Independent self-construal (1-6) | -0.24 | 0.22 | -0.07 | 0.273 | -0.67 | 0.19 |
| Interdependent self-construal (1-6) | 0.08 | 0.18 | 0.03 | 0.660 | -0.28 | 0.44 |
| Peak happiness in solitude (0-10) | **-0.27** | **0.08** | **-0.24** | **0.001** | **-0.42** | **-0.11** |
| Recent happiness in solitude (0-10) | **-0.19** | **0.06** | **-0.23** | **0.002** | **-0.32** | **-0.07** |
| Current happiness (0-10) | 0.08 | 0.06 | 0.10 | 0.141 | -0.03 | 0.19 |
| Days elapsed since ESM | 0.01 | 0.01 | 0.06 | 0.340 | -0.01 | 0.04 |
| Age (years) | 0.02 | 0.01 | 0.10 | 0.094 | 0.00 | 0.05 |
| Gender (1 = male) | -0.16 | 0.26 | -0.04 | 0.535 | -0.66 | 0.35 |
| Location (1 = Hong Kong) | -0.23 | 0.28 | -0.05 | 0.405 | -0.77 | 0.31 |
| Living alone (1 = yes) | 1.05 | 0.44 | 0.14 | 0.019 | 0.17 | 1.92 |
| Subjective social status (1-10) | -0.05 | 0.08 | -0.04 | 0.558 | -0.20 | 0.11 |
| Marital status (1 = married) | 0.44 | 0.28 | 0.09 | 0.117 | -0.11 | 0.98 |
| Education (1 = some post-secondary) | -0.18 | 0.33 | -0.03 | 0.580 | -0.83 | 0.47 |
| Income (1 = at or above median) | -0.06 | 0.25 | -0.01 | 0.819 | -0.55 | 0.44 |
| Proportion assessments in solitude | 0.50 | 0.61 | 0.05 | 0.412 | -0.70 | 1.70 |

Table S14

*Model predicting discrepancy between retrospective and mean momentary reports of energy in solitude (no in-person or virtual interaction)*

| **Variable** | ***b*** | ***SE*** | ***β*** | ***p*** | ***95% CI(l)*** | ***95% CI(u)*** |
| --- | --- | --- | --- | --- | --- | --- |
| Intercept | **0.43** | **0.12** | **0.01** | **0.000** | **0.20** | **0.66** |
| Introversion (1-5) | 0.12 | 0.17 | 0.04 | 0.498 | -0.22 | 0.46 |
| Self-determined solitude (1-5) | **0.54** | **0.20** | **0.17** | **0.008** | **0.14** | **0.94** |
| Not-self-determined solitude (1-5) | 0.20 | 0.19 | 0.06 | 0.305 | -0.18 | 0.58 |
| Independent self-construal (1-6) | -0.11 | 0.22 | -0.03 | 0.621 | -0.54 | 0.32 |
| Interdependent self-construal (1-6) | **0.49** | **0.18** | **0.17** | **0.007** | **0.13** | **0.85** |
| Peak energy in solitude (0-10) | **-0.25** | **0.07** | **-0.25** | **0.001** | **-0.39** | **-0.11** |
| Recent energy in solitude (0-10) | **-0.21** | **0.07** | **-0.23** | **0.002** | **-0.35** | **-0.08** |
| Current energy (0-10) | **0.17** | **0.06** | **0.20** | **0.002** | **0.06** | **0.28** |
| Days elapsed since ESM | 0.00 | 0.01 | 0.02 | 0.770 | -0.02 | 0.03 |
| Age (years) | 0.01 | 0.01 | 0.05 | 0.419 | -0.02 | 0.04 |
| Gender (1 = male) | -0.19 | 0.26 | -0.04 | 0.462 | -0.70 | 0.32 |
| Location (1 = Hong Kong) | -0.43 | 0.28 | -0.10 | 0.122 | -0.97 | 0.12 |
| Living alone (1 = yes) | 0.44 | 0.45 | 0.06 | 0.329 | -0.45 | 1.32 |
| Subjective social status (1-10) | -0.04 | 0.08 | -0.03 | 0.601 | -0.20 | 0.11 |
| Marital status (1 = married) | 0.19 | 0.28 | 0.04 | 0.504 | -0.37 | 0.75 |
| Education (1 = some post-secondary) | 0.14 | 0.34 | 0.02 | 0.672 | -0.52 | 0.80 |
| Income (1 = at or above median) | 0.02 | 0.26 | 0.01 | 0.927 | -0.49 | 0.53 |
| Proportion assessments in solitude | 0.02 | 0.62 | 0.00 | 0.975 | -1.20 | 1.24 |

Table S15

*Model predicting discrepancy between retrospective and mean momentary reports of calm in solitude (no in-person or virtual interaction)*

| **Variable** | ***b*** | ***SE*** | ***β*** | ***p*** | ***95% CI(l)*** | ***95% CI(u)*** |
| --- | --- | --- | --- | --- | --- | --- |
| Intercept | **0.34** | **0.12** | **-0.01** | **0.004** | **0.11** | **0.57** |
| Introversion (1-5) | 0.26 | 0.17 | 0.10 | 0.135 | -0.08 | 0.60 |
| Self-determined solitude (1-5) | **0.88** | **0.19** | **0.28** | **0.000** | **0.50** | **1.26** |
| Not-self-determined solitude (1-5) | -0.36 | 0.19 | -0.12 | 0.058 | -0.74 | 0.01 |
| Independent self-construal (1-6) | -0.07 | 0.22 | -0.02 | 0.752 | -0.50 | 0.36 |
| Interdependent self-construal (1-6) | -0.11 | 0.18 | -0.04 | 0.537 | -0.47 | 0.25 |
| Peak calm in solitude (0-10) | -0.17 | 0.09 | -0.14 | 0.055 | -0.34 | 0.00 |
| Recent calm in solitude (0-10) | -0.16 | 0.07 | -0.18 | 0.014 | -0.29 | -0.03 |
| Current calm (0-10) | **0.16** | **0.05** | **0.20** | **0.003** | **0.05** | **0.27** |
| Days elapsed since ESM | 0.00 | 0.01 | -0.01 | 0.834 | -0.03 | 0.02 |
| Age (years) | -0.01 | 0.01 | -0.04 | 0.487 | -0.04 | 0.02 |
| Gender (1 = male) | 0.18 | 0.26 | 0.04 | 0.496 | -0.34 | 0.69 |
| Location (1 = Hong Kong) | -0.52 | 0.27 | -0.13 | 0.060 | -1.06 | 0.02 |
| Living alone (1 = yes) | 0.04 | 0.45 | 0.01 | 0.927 | -0.84 | 0.92 |
| Subjective social status (1-10) | -0.13 | 0.08 | -0.10 | 0.091 | -0.29 | 0.02 |
| Marital status (1 = married) | 0.23 | 0.28 | 0.05 | 0.404 | -0.32 | 0.79 |
| Education (1 = some post-secondary) | -0.02 | 0.33 | 0.00 | 0.950 | -0.67 | 0.63 |
| Income (1 = at or above median) | 0.33 | 0.26 | 0.08 | 0.202 | -0.18 | 0.83 |
| Proportion assessments in solitude | 0.47 | 0.61 | 0.05 | 0.441 | -0.73 | 1.68 |

Table S16

*Model predicting discrepancy between retrospective and mean momentary reports of relaxation in solitude (no in-person or virtual interaction)*

| **Variable** | ***b*** | ***SE*** | ***β*** | ***p*** | ***95% CI(l)*** | ***95% CI(u)*** |
| --- | --- | --- | --- | --- | --- | --- |
| Intercept | **0.72** | **0.13** | **0.00** | **0.000** | **0.46** | **0.97** |
| Introversion (1-5) | 0.07 | 0.19 | 0.02 | 0.709 | -0.30 | 0.44 |
| Self-determined solitude (1-5) | **0.91** | **0.21** | **0.27** | **0.000** | **0.50** | **1.32** |
| Not-self-determined solitude (1-5) | -0.21 | 0.21 | -0.06 | 0.311 | -0.62 | 0.20 |
| Independent self-construal (1-6) | -0.31 | 0.24 | -0.08 | 0.200 | -0.78 | 0.16 |
| Interdependent self-construal (1-6) | -0.09 | 0.20 | -0.03 | 0.664 | -0.48 | 0.30 |
| Peak relaxation in solitude (0-10) | -0.15 | 0.09 | -0.12 | 0.091 | -0.33 | 0.02 |
| Recent relaxation in solitude (0-10) | **-0.26** | **0.07** | **-0.27** | **0.000** | **-0.39** | **-0.13** |
| Current relaxation (0-10) | 0.10 | 0.06 | 0.12 | 0.064 | -0.01 | 0.21 |
| Days elapsed since ESM | 0.00 | 0.01 | -0.01 | 0.860 | -0.03 | 0.02 |
| Age (years) | -0.01 | 0.02 | -0.02 | 0.697 | -0.04 | 0.02 |
| Gender (1 = male) | -0.02 | 0.28 | 0.00 | 0.945 | -0.57 | 0.53 |
| Location (1 = Hong Kong) | -0.24 | 0.30 | -0.05 | 0.424 | -0.82 | 0.35 |
| Living alone (1 = yes) | 0.67 | 0.49 | 0.08 | 0.171 | -0.29 | 1.63 |
| Subjective social status (1-10) | -0.07 | 0.08 | -0.05 | 0.428 | -0.23 | 0.10 |
| Marital status (1 = married) | 0.28 | 0.31 | 0.06 | 0.356 | -0.32 | 0.88 |
| Education (1 = some post-secondary) | 0.61 | 0.36 | 0.10 | 0.091 | -0.10 | 1.33 |
| Income (1 = at or above median) | 0.24 | 0.28 | 0.05 | 0.396 | -0.31 | 0.78 |
| Proportion assessments in solitude | 0.14 | 0.65 | 0.01 | 0.828 | -1.14 | 1.43 |

Table S17

*Model predicting discrepancy between retrospective and mean momentary reports of irritation in solitude (no in-person or virtual interaction)*

| **Variable** | ***b*** | ***SE*** | ***β*** | ***p*** | ***95% CI(l)*** | ***95% CI(u)*** |
| --- | --- | --- | --- | --- | --- | --- |
| Intercept | **-0.58** | **0.11** | **-0.02** | **0.000** | **-0.80** | **-0.37** |
| Introversion (1-5) | -0.19 | 0.16 | -0.08 | 0.224 | -0.50 | 0.12 |
| Self-determined solitude (1-5) | -0.37 | 0.17 | -0.12 | 0.034 | -0.72 | -0.03 |
| Not-self-determined solitude (1-5) | 0.33 | 0.18 | 0.12 | 0.062 | -0.02 | 0.68 |
| Independent self-construal (1-6) | 0.28 | 0.20 | 0.09 | 0.161 | -0.11 | 0.68 |
| Interdependent self-construal (1-6) | -0.18 | 0.17 | -0.07 | 0.291 | -0.50 | 0.15 |
| Peak irritation in solitude (0-10) | **-0.20** | **0.05** | **-0.31** | **0.000** | **-0.29** | **-0.11** |
| Recent irritation in solitude (0-10) | **-0.20** | **0.06** | **-0.25** | **0.001** | **-0.32** | **-0.09** |
| Current irritation (0-10) | **0.16** | **0.06** | **0.18** | **0.004** | **0.05** | **0.28** |
| Days elapsed since ESM | 0.00 | 0.01 | 0.02 | 0.788 | -0.02 | 0.03 |
| Age (years) | -0.01 | 0.01 | -0.02 | 0.700 | -0.03 | 0.02 |
| Gender (1 = male) | 0.19 | 0.23 | 0.05 | 0.423 | -0.27 | 0.65 |
| Location (1 = Hong Kong) | -0.34 | 0.26 | -0.09 | 0.194 | -0.86 | 0.18 |
| Living alone (1 = yes) | -0.14 | 0.41 | -0.02 | 0.735 | -0.95 | 0.67 |
| Subjective social status (1-10) | 0.10 | 0.07 | 0.09 | 0.145 | -0.04 | 0.25 |
| Marital status (1 = married) | -0.21 | 0.26 | -0.05 | 0.431 | -0.72 | 0.31 |
| Education (1 = some post-secondary) | -0.40 | 0.31 | -0.07 | 0.197 | -1.01 | 0.21 |
| Income (1 = at or above median) | 0.02 | 0.24 | 0.00 | 0.935 | -0.45 | 0.48 |
| Proportion assessments in solitude | 1.44 | 0.58 | 0.16 | 0.013 | 0.30 | 2.57 |

Table S18

*Model predicting discrepancy between retrospective and mean momentary reports of anxiety in solitude (no in-person or virtual interaction)*

| **Variable** | ***b*** | ***SE*** | ***β*** | ***p*** | ***95% CI(l)*** | ***95% CI(u)*** |
| --- | --- | --- | --- | --- | --- | --- |
| Intercept | -0.19 | 0.12 | 0.00 | 0.094 | -0.42 | 0.03 |
| Introversion (1-5) | 0.21 | 0.17 | 0.08 | 0.215 | -0.12 | 0.54 |
| Self-determined solitude (1-5) | -0.46 | 0.19 | -0.14 | 0.015 | -0.82 | -0.09 |
| Not-self-determined solitude (1-5) | 0.06 | 0.19 | 0.02 | 0.765 | -0.32 | 0.44 |
| Independent self-construal (1-6) | 0.01 | 0.22 | 0.00 | 0.967 | -0.42 | 0.43 |
| Interdependent self-construal (1-6) | 0.34 | 0.18 | 0.12 | 0.057 | -0.01 | 0.69 |
| Peak anxiety in solitude (0-10) | -0.08 | 0.06 | -0.11 | 0.183 | -0.21 | 0.04 |
| Recent anxiety in solitude (0-10) | **-0.32** | **0.07** | **-0.39** | **0.000** | **-0.45** | **-0.18** |
| Current anxiety (0-10) | **0.25** | **0.05** | **0.32** | **0.000** | **0.14** | **0.35** |
| Days elapsed since ESM | -0.03 | 0.01 | -0.13 | 0.033 | -0.05 | 0.00 |
| Age (years) | -0.02 | 0.01 | -0.09 | 0.136 | -0.05 | 0.01 |
| Gender (1 = male) | -0.22 | 0.25 | -0.05 | 0.387 | -0.71 | 0.28 |
| Location (1 = Hong Kong) | -0.10 | 0.27 | -0.02 | 0.722 | -0.63 | 0.44 |
| Living alone (1 = yes) | -0.42 | 0.44 | -0.06 | 0.336 | -1.28 | 0.44 |
| Subjective social status (1-10) | 0.05 | 0.08 | 0.04 | 0.491 | -0.10 | 0.20 |
| Marital status (1 = married) | -0.43 | 0.28 | -0.09 | 0.120 | -0.97 | 0.11 |
| Education (1 = some post-secondary) | -0.42 | 0.33 | -0.07 | 0.201 | -1.07 | 0.23 |
| Income (1 = at or above median) | -0.12 | 0.25 | -0.03 | 0.625 | -0.61 | 0.37 |
| Proportion assessments in solitude | 0.21 | 0.62 | 0.02 | 0.734 | -1.01 | 1.43 |

Table S19

*Model predicting discrepancy between retrospective and mean momentary reports of loneliness in solitude (no in-person or virtual interaction)*

| **Variable** | ***b*** | ***SE*** | ***β*** | ***p*** | ***95% CI(l)*** | ***95% CI(u)*** |
| --- | --- | --- | --- | --- | --- | --- |
| Intercept | **0.63** | **0.13** | **0.00** | **0.000** | **0.38** | **0.89** |
| Introversion (1-5) | 0.16 | 0.19 | 0.05 | 0.409 | -0.22 | 0.53 |
| Self-determined solitude (1-5) | **-0.69** | **0.21** | **-0.20** | **0.002** | **-1.11** | **-0.26** |
| Not-self-determined solitude (1-5) | **0.63** | **0.22** | **0.19** | **0.004** | **0.20** | **1.07** |
| Independent self-construal (1-6) | -0.17 | 0.25 | -0.05 | 0.486 | -0.66 | 0.32 |
| Interdependent self-construal (1-6) | **0.70** | **0.20** | **0.23** | **0.001** | **0.30** | **1.10** |
| Peak loneliness in solitude (0-10) | -0.10 | 0.07 | -0.14 | 0.140 | -0.24 | 0.03 |
| Recent loneliness in solitude (0-10) | -0.10 | 0.08 | -0.11 | 0.228 | -0.26 | 0.06 |
| Current loneliness (0-10) | 0.11 | 0.06 | 0.13 | 0.078 | -0.01 | 0.23 |
| Days elapsed since ESM | 0.01 | 0.01 | 0.02 | 0.714 | -0.02 | 0.03 |
| Age (years) | **-0.06** | **0.02** | **-0.23** | **0.000** | **-0.09** | **-0.03** |
| Gender (1 = male) | -0.06 | 0.28 | -0.01 | 0.825 | -0.62 | 0.49 |
| Location (1 = Hong Kong) | -0.07 | 0.30 | -0.01 | 0.825 | -0.67 | 0.53 |
| Living alone (1 = yes) | 0.35 | 0.50 | 0.04 | 0.483 | -0.63 | 1.33 |
| Subjective social status (1-10) | 0.15 | 0.09 | 0.11 | 0.082 | -0.02 | 0.32 |
| Marital status (1 = married) | -0.23 | 0.31 | -0.05 | 0.462 | -0.84 | 0.38 |
| Education (1 = some post-secondary) | 0.10 | 0.37 | 0.02 | 0.781 | -0.63 | 0.83 |
| Income (1 = at or above median) | -0.14 | 0.28 | -0.03 | 0.612 | -0.70 | 0.41 |
| Proportion assessments in solitude | 0.55 | 0.67 | 0.05 | 0.411 | -0.77 | 1.87 |

Table S20

*Model predicting discrepancy between retrospective and mean momentary reports of tiredness in solitude (no in-person or virtual interaction)*

| **Variable** | ***b*** | ***SE*** | ***β*** | ***p*** | ***95% CI(l)*** | ***95% CI(u)*** |
| --- | --- | --- | --- | --- | --- | --- |
| Intercept | **-0.93** | **0.13** | **-0.02** | **0.000** | **-1.18** | **-0.68** |
| Introversion (1-5) | 0.09 | 0.19 | 0.03 | 0.644 | -0.28 | 0.45 |
| Self-determined solitude (1-5) | -0.06 | 0.21 | -0.01 | 0.780 | -0.47 | 0.35 |
| Not-self-determined solitude (1-5) | 0.15 | 0.20 | 0.04 | 0.475 | -0.26 | 0.55 |
| Independent self-construal (1-6) | -0.06 | 0.24 | -0.01 | 0.804 | -0.52 | 0.40 |
| Interdependent self-construal (1-6) | 0.01 | 0.19 | 0.00 | 0.946 | -0.37 | 0.40 |
| Peak tiredness in solitude (0-10) | **-0.43** | **0.08** | **-0.37** | **0.000** | **-0.58** | **-0.27** |
| Recent tiredness in solitude (0-10) | -0.15 | 0.06 | -0.15 | 0.017 | -0.28 | -0.03 |
| Current tiredness (0-10) | **0.23** | **0.05** | **0.26** | **0.000** | **0.13** | **0.32** |
| Days elapsed since ESM | 0.00 | 0.01 | -0.01 | 0.858 | -0.03 | 0.02 |
| Age (years) | -0.04 | 0.02 | -0.13 | 0.015 | -0.07 | -0.01 |
| Gender (1 = male) | 0.00 | 0.28 | 0.00 | 0.989 | -0.54 | 0.55 |
| Location (1 = Hong Kong) | **-1.65** | **0.30** | **-0.32** | **0.000** | **-2.24** | **-1.06** |
| Living alone (1 = yes) | -0.82 | 0.48 | -0.09 | 0.090 | -1.77 | 0.13 |
| Subjective social status (1-10) | 0.21 | 0.08 | 0.13 | 0.014 | 0.04 | 0.38 |
| Marital status (1 = married) | -0.22 | 0.30 | -0.04 | 0.469 | -0.81 | 0.38 |
| Education (1 = some post-secondary) | -0.47 | 0.36 | -0.07 | 0.196 | -1.17 | 0.24 |
| Income (1 = at or above median) | -0.32 | 0.27 | -0.06 | 0.241 | -0.86 | 0.22 |
| Proportion assessments in solitude | 1.43 | 0.67 | 0.12 | 0.035 | 0.10 | 2.76 |

# Supplementary Materials C: Exploratory analyses of in-person social interaction

We examined whether solitude-related self-concepts shape participants’ recall of how they felt during in-person social interaction (instead of solitude), in a set of models that parallel those used in our main analysis. Retrospective reports of affect during in-person interaction were estimated for each affective state by taking the average of *retrospective [happy] during close interaction* and *retrospective [happy] during not-close interaction*, from the study post-survey. We computed each participant’s mean momentary affect at moments of in-person interaction during the ESM period. Then, we subtracted mean momentary reports from the respective retrospective reports to compute retrospective report discrepancies (for in-person interaction) for each affective state, as reported in *Table S21*.

Models predicting retrospective report discrepancies are reported in *Tables S22-S29*; these include the same predictors as the main study models, except they control for peak and recent affect during in-person interaction, and proportion of ESM assessments spent in in-person interaction. When recalling how they felt during in-person social interaction, individuals tended to underestimate their feelings of calm, relaxation, and tiredness - all low-arousal affective states. Higher NSDS was associated with overestimating anxiety and irritation during interaction. Notably also, individuals living in the HK (vs. UK) were more prone to underestimate their irritation and tiredness during interaction.

Table S21

*Retrospective report discrepancies (retrospective report minus mean of momentary reports) during in-person social interaction for eight affective states*

| **Affective state** | **Retrospective report  *M* (*SD*)** | **Mean momentary report  *M* (*SD*)** | **Retrospective report discrepancy *M* (*SD*)** |
| --- | --- | --- | --- |
| Happy | 5.75 (1.71) | 5.91 (1.80) | -0.16 (1.66) |
| Energised | 4.99 (1.86) | 5.03 (1.66) | -0.04 (1.70) |
| Calm | 5.57 (1.91) | 6.00 (1.74) | -0.43 (1.74) |
| Relaxed | 5.59 (1.89) | 5.91 (1.67) | -0.32 (1.81) |
| Anxious | 3.20 (2.07) | 2.99 (1.89) | 0.20 (1.63) |
| Irritated | 2.59 (1.82) | 2.59 (1.66) | -0.01 (1.59) |
| Lonely | 2.28 (1.99) | 2.26 (1.87) | 0.02 (1.53) |
| Tired | 4.38 (2.02) | 4.90 (1.67) | -0.53 (1.80) |

*Note:* Retrospective and mean momentary reports are on a scale from 0 to 10

Table S22

*Model predicting discrepancy between retrospective and mean momentary reports of happiness during social interaction (in-person interaction)*

| **Variable** | ***b*** | ***SE*** | ***β*** | ***p*** | ***95% CI(l)*** | ***95% CI(u)*** |
| --- | --- | --- | --- | --- | --- | --- |
| Intercept | -0.15 | 0.08 | 0.00 | 0.060 | -0.31 | 0.01 |
| Introversion (1-5) | -0.15 | 0.12 | -0.07 | 0.216 | -0.38 | 0.09 |
| Self-determined solitude (1-5) | 0.03 | 0.14 | 0.01 | 0.818 | -0.24 | 0.31 |
| Not-self-determined solitude (1-5) | -0.21 | 0.14 | -0.09 | 0.125 | -0.48 | 0.06 |
| Independent self-construal (1-6) | 0.04 | 0.15 | 0.02 | 0.787 | -0.26 | 0.34 |
| Interdependent self-construal (1-6) | 0.28 | 0.13 | 0.12 | 0.028 | 0.03 | 0.52 |
| Peak happiness in social interaction (0-10) | **-0.34** | **0.07** | **-0.36** | **0.000** | **-0.47** | **-0.21** |
| Recent happiness in social interaction (0-10) | **-0.28** | **0.05** | **-0.36** | **0.000** | **-0.38** | **-0.18** |
| Current happiness (0-10) | 0.08 | 0.04 | 0.12 | 0.062 | 0.00 | 0.16 |
| Days elapsed since ESM | -0.01 | 0.01 | -0.04 | 0.459 | -0.02 | 0.01 |
| Age (years) | 0.01 | 0.01 | 0.04 | 0.477 | -0.01 | 0.03 |
| Gender (1 = male) | -0.19 | 0.18 | -0.06 | 0.298 | -0.54 | 0.17 |
| Location (1 = Hong Kong) | -0.37 | 0.20 | -0.11 | 0.060 | -0.76 | 0.02 |
| Living alone (1 = yes) | -0.04 | 0.31 | -0.01 | 0.899 | -0.66 | 0.58 |
| Subjective social status (1-10) | -0.02 | 0.05 | -0.02 | 0.682 | -0.13 | 0.08 |
| Marital status (1 = married) | -0.09 | 0.20 | -0.02 | 0.647 | -0.47 | 0.29 |
| Education (1 = some post-secondary) | -0.06 | 0.23 | -0.01 | 0.798 | -0.52 | 0.40 |
| Income (1 = at or above median) | -0.13 | 0.18 | -0.04 | 0.456 | -0.48 | 0.22 |
| Proportion assessments in social interaction | 1.03 | 0.41 | 0.15 | 0.012 | 0.23 | 1.83 |

Table S23

*Model predicting discrepancy between retrospective and mean momentary reports of feeling energised during social interaction (in-person interaction)*

| **Variable** | ***b*** | ***SE*** | ***β*** | ***p*** | ***95% CI(l)*** | ***95% CI(u)*** |
| --- | --- | --- | --- | --- | --- | --- |
| Intercept | -0.04 | 0.09 | 0.00 | 0.637 | -0.21 | 0.13 |
| Introversion (1-5) | -0.07 | 0.13 | -0.03 | 0.608 | -0.32 | 0.19 |
| Self-determined solitude (1-5) | 0.12 | 0.15 | 0.05 | 0.423 | -0.18 | 0.42 |
| Not-self-determined solitude (1-5) | -0.20 | 0.15 | -0.08 | 0.175 | -0.48 | 0.09 |
| Independent self-construal (1-6) | -0.15 | 0.16 | -0.06 | 0.347 | -0.47 | 0.17 |
| Interdependent self-construal (1-6) | 0.33 | 0.13 | 0.14 | 0.014 | 0.07 | 0.59 |
| Peak energy in social interaction (0-10) | **-0.37** | **0.06** | **-0.40** | **0.000** | **-0.49** | **-0.24** |
| Recent energy in social interaction (0-10) | **-0.14** | **0.05** | **-0.18** | **0.008** | **-0.24** | **-0.04** |
| Current energy (0-10) | 0.09 | 0.04 | 0.13 | 0.028 | 0.01 | 0.17 |
| Days elapsed since ESM | 0.00 | 0.01 | 0.01 | 0.908 | -0.02 | 0.02 |
| Age (years) | 0.00 | 0.01 | -0.02 | 0.678 | -0.03 | 0.02 |
| Gender (1 = male) | -0.08 | 0.20 | -0.02 | 0.693 | -0.46 | 0.31 |
| Location (1 = Hong Kong) | -0.14 | 0.21 | -0.04 | 0.510 | -0.54 | 0.27 |
| Living alone (1 = yes) | 0.52 | 0.34 | 0.09 | 0.122 | -0.14 | 1.19 |
| Subjective social status (1-10) | 0.05 | 0.06 | 0.05 | 0.406 | -0.07 | 0.16 |
| Marital status (1 = married) | 0.21 | 0.21 | 0.06 | 0.310 | -0.20 | 0.62 |
| Education (1 = some post-secondary) | -0.17 | 0.25 | -0.04 | 0.487 | -0.66 | 0.32 |
| Income (1 = at or above median) | -0.07 | 0.19 | -0.02 | 0.700 | -0.45 | 0.30 |
| Proportion assessments in social interaction | 0.90 | 0.43 | 0.13 | 0.037 | 0.06 | 1.74 |

Table S24

*Model predicting discrepancy between retrospective and mean momentary reports of calm during social interaction (in-person interaction)*

| **Variable** | ***b*** | ***SE*** | ***β*** | ***p*** | ***95% CI(l)*** | ***95% CI(u)*** |
| --- | --- | --- | --- | --- | --- | --- |
| Intercept | **-0.42** | **0.09** | **0.00** | **0.000** | **-0.60** | **-0.25** |
| Introversion (1-5) | -0.03 | 0.13 | 0.00 | 0.824 | -0.28 | 0.23 |
| Self-determined solitude (1-5) | 0.20 | 0.15 | 0.08 | 0.188 | -0.10 | 0.49 |
| Not-self-determined solitude (1-5) | -0.28 | 0.15 | 0.10 | 0.062 | -0.58 | 0.01 |
| Independent self-construal (1-6) | -0.33 | 0.16 | -0.03 | 0.043 | -0.66 | -0.01 |
| Interdependent self-construal (1-6) | 0.00 | 0.14 | -0.07 | 0.992 | -0.27 | 0.27 |
| Peak calm in social interaction (0-10) | **-0.31** | **0.07** | **-0.22** | **0.000** | **-0.44** | **-0.17** |
| Recent calm in social interaction (0-10) | **-0.19** | **0.06** | **-0.28** | **0.001** | **-0.30** | **-0.08** |
| Current calm (0-10) | **0.12** | **0.04** | **0.30** | **0.003** | **0.04** | **0.20** |
| Days elapsed since ESM | -0.01 | 0.01 | 0.04 | 0.188 | -0.03 | 0.01 |
| Age (years) | 0.01 | 0.01 | -0.04 | 0.573 | -0.02 | 0.03 |
| Gender (1 = male) | 0.03 | 0.20 | -0.10 | 0.889 | -0.36 | 0.41 |
| Location (1 = Hong Kong) | -0.26 | 0.21 | -0.19 | 0.209 | -0.68 | 0.15 |
| Living alone (1 = yes) | 0.06 | 0.34 | 0.01 | 0.861 | -0.62 | 0.74 |
| Subjective social status (1-10) | -0.04 | 0.06 | 0.00 | 0.478 | -0.16 | 0.07 |
| Marital status (1 = married) | 0.10 | 0.21 | 0.05 | 0.650 | -0.32 | 0.51 |
| Education (1 = some post-secondary) | 0.32 | 0.25 | -0.05 | 0.206 | -0.18 | 0.82 |
| Income (1 = at or above median) | -0.04 | 0.19 | 0.07 | 0.824 | -0.42 | 0.34 |
| Proportion assessments in social interaction | -0.19 | 0.44 | 0.01 | 0.663 | -1.06 | 0.68 |

Table S25

*Model predicting discrepancy between retrospective and mean momentary reports of relaxation during social interaction (in-person interaction)*

| **Variable** | ***b*** | ***SE*** | ***β*** | ***p*** | ***95% CI(l)*** | ***95% CI(u)*** |
| --- | --- | --- | --- | --- | --- | --- |
| Intercept | **-0.31** | **0.09** | **0.00** | **0.001** | **-0.48** | **-0.13** |
| Introversion (1-5) | -0.04 | 0.13 | -0.02 | 0.740 | -0.30 | 0.21 |
| Self-determined solitude (1-5) | 0.25 | 0.15 | 0.09 | 0.102 | -0.05 | 0.54 |
| Not-self-determined solitude (1-5) | -0.26 | 0.15 | -0.10 | 0.089 | -0.55 | 0.04 |
| Independent self-construal (1-6) | -0.08 | 0.16 | -0.03 | 0.630 | -0.40 | 0.25 |
| Interdependent self-construal (1-6) | 0.19 | 0.14 | 0.08 | 0.166 | -0.08 | 0.46 |
| Peak relaxation in social interaction (0-10) | **-0.50** | **0.07** | **-0.47** | **0.000** | **-0.64** | **-0.35** |
| Recent relaxation in social interaction (0-10) | -0.08 | 0.05 | -0.10 | 0.129 | -0.19 | 0.02 |
| Current relaxation (0-10) | 0.10 | 0.04 | 0.14 | 0.015 | 0.02 | 0.17 |
| Days elapsed since ESM | -0.01 | 0.01 | -0.05 | 0.361 | -0.03 | 0.01 |
| Age (years) | 0.02 | 0.01 | 0.11 | 0.041 | 0.00 | 0.04 |
| Gender (1 = male) | -0.42 | 0.20 | -0.11 | 0.036 | -0.81 | -0.03 |
| Location (1 = Hong Kong) | -0.48 | 0.21 | -0.13 | 0.024 | -0.90 | -0.06 |
| Living alone (1 = yes) | -0.11 | 0.35 | -0.02 | 0.758 | -0.79 | 0.57 |
| Subjective social status (1-10) | 0.03 | 0.06 | 0.02 | 0.648 | -0.09 | 0.14 |
| Marital status (1 = married) | 0.42 | 0.21 | 0.10 | 0.052 | 0.00 | 0.84 |
| Education (1 = some post-secondary) | 0.17 | 0.25 | 0.03 | 0.512 | -0.33 | 0.67 |
| Income (1 = at or above median) | -0.39 | 0.19 | -0.11 | 0.044 | -0.78 | -0.01 |
| Proportion assessments in social interaction | 0.60 | 0.44 | 0.08 | 0.174 | -0.27 | 1.46 |

Table S26

*Model predicting discrepancy between retrospective and mean momentary reports of irritation during social interaction (in-person interaction)*

| **Variable** | ***b*** | ***SE*** | ***β*** | ***p*** | ***95% CI(l)*** | ***95% CI(u)*** |
| --- | --- | --- | --- | --- | --- | --- |
| Intercept | -0.01 | 0.08 | 0.00 | 0.950 | -0.17 | 0.16 |
| Introversion (1-5) | 0.01 | 0.12 | 0.01 | 0.912 | -0.22 | 0.25 |
| Self-determined solitude (1-5) | -0.06 | 0.13 | -0.03 | 0.629 | -0.33 | 0.20 |
| Not-self-determined solitude (1-5) | **0.39** | **0.14** | **0.17** | **0.005** | **0.12** | **0.66** |
| Independent self-construal (1-6) | -0.05 | 0.15 | -0.02 | 0.734 | -0.35 | 0.24 |
| Interdependent self-construal (1-6) | -0.16 | 0.12 | -0.08 | 0.185 | -0.40 | 0.08 |
| Peak irritation in social interaction (0-10) | **-0.13** | **0.03** | **-0.24** | **0.000** | **-0.20** | **-0.06** |
| Recent irritation in social interaction (0-10) | **-0.24** | **0.05** | **-0.30** | **0.000** | **-0.35** | **-0.14** |
| Current irritation (0-10) | **0.18** | **0.04** | **0.24** | **0.000** | **0.10** | **0.27** |
| Days elapsed since ESM | 0.01 | 0.01 | 0.09 | 0.128 | 0.00 | 0.03 |
| Age (years) | 0.00 | 0.01 | 0.01 | 0.848 | -0.02 | 0.02 |
| Gender (1 = male) | -0.08 | 0.18 | -0.03 | 0.650 | -0.43 | 0.27 |
| Location (1 = Hong Kong) | **-0.58** | **0.20** | **-0.18** | **0.003** | **-0.97** | **-0.19** |
| Living alone (1 = yes) | 0.09 | 0.31 | 0.02 | 0.780 | -0.53 | 0.71 |
| Subjective social status (1-10) | 0.00 | 0.05 | 0.00 | 0.968 | -0.11 | 0.10 |
| Marital status (1 = married) | -0.06 | 0.19 | -0.02 | 0.776 | -0.44 | 0.33 |
| Education (1 = some post-secondary) | -0.45 | 0.23 | -0.10 | 0.056 | -0.90 | 0.01 |
| Income (1 = at or above median) | 0.16 | 0.18 | 0.05 | 0.358 | -0.19 | 0.51 |
| Proportion assessments in social interaction | -0.07 | 0.39 | -0.01 | 0.858 | -0.83 | 0.69 |

Table S27

*Model predicting discrepancy between retrospective and mean momentary reports of anxiety during social interaction (in-person interaction)*

| **Variable** | ***b*** | ***SE*** | ***β*** | ***p*** | ***95% CI(l)*** | ***95% CI(u)*** |
| --- | --- | --- | --- | --- | --- | --- |
| Intercept | 0.18 | 0.08 | 0.00 | 0.028 | 0.02 | 0.34 |
| Introversion (1-5) | 0.31 | 0.12 | 0.15 | 0.011 | 0.07 | 0.54 |
| Self-determined solitude (1-5) | -0.08 | 0.13 | -0.03 | 0.549 | -0.35 | 0.18 |
| Not-self-determined solitude (1-5) | **0.40** | **0.14** | **0.17** | **0.006** | **0.12** | **0.69** |
| Independent self-construal (1-6) | 0.03 | 0.15 | 0.01 | 0.845 | -0.27 | 0.33 |
| Interdependent self-construal (1-6) | 0.16 | 0.12 | 0.07 | 0.193 | -0.08 | 0.41 |
| Peak anxiety in social interaction (0-10) | -0.08 | 0.04 | -0.14 | 0.047 | -0.16 | 0.00 |
| Recent anxiety in social interaction (0-10) | **-0.25** | **0.05** | **-0.36** | **0.000** | **-0.34** | **-0.15** |
| Current anxiety (0-10) | **0.11** | **0.04** | **0.18** | **0.004** | **0.04** | **0.18** |
| Days elapsed since ESM | 0.00 | 0.01 | -0.01 | 0.791 | -0.02 | 0.02 |
| Age (years) | -0.03 | 0.01 | -0.15 | 0.011 | -0.05 | -0.01 |
| Gender (1 = male) | 0.06 | 0.18 | 0.02 | 0.753 | -0.30 | 0.41 |
| Location (1 = Hong Kong) | -0.56 | 0.20 | -0.17 | 0.004 | -0.95 | -0.18 |
| Living alone (1 = yes) | -0.48 | 0.32 | -0.08 | 0.131 | -1.10 | 0.14 |
| Subjective social status (1-10) | -0.03 | 0.05 | -0.03 | 0.530 | -0.14 | 0.07 |
| Marital status (1 = married) | -0.13 | 0.20 | -0.04 | 0.509 | -0.53 | 0.26 |
| Education (1 = some post-secondary) | -0.42 | 0.24 | -0.09 | 0.078 | -0.89 | 0.05 |
| Income (1 = at or above median) | 0.33 | 0.18 | 0.10 | 0.067 | -0.02 | 0.69 |
| Proportion assessments in social interaction | -0.79 | 0.40 | -0.11 | 0.048 | -1.56 | -0.01 |

Table S28

*Model predicting discrepancy between retrospective and mean momentary reports of loneliness during social interaction (in-person interaction)*

| **Variable** | ***b*** | ***SE*** | ***β*** | ***p*** | ***95% CI(l)*** | ***95% CI(u)*** |
| --- | --- | --- | --- | --- | --- | --- |
| Intercept | 0.03 | 0.08 | 0.00 | 0.725 | -0.13 | 0.18 |
| Introversion (1-5) | 0.12 | 0.11 | 0.06 | 0.293 | -0.10 | 0.34 |
| Self-determined solitude (1-5) | -0.02 | 0.13 | -0.01 | 0.856 | -0.28 | 0.23 |
| Not-self-determined solitude (1-5) | 0.19 | 0.14 | 0.09 | 0.155 | -0.07 | 0.46 |
| Independent self-construal (1-6) | -0.02 | 0.15 | -0.01 | 0.869 | -0.31 | 0.26 |
| Interdependent self-construal (1-6) | 0.02 | 0.12 | 0.01 | 0.844 | -0.21 | 0.25 |
| Peak loneliness in social interaction (0-10) | **-0.19** | **0.04** | **-0.37** | **0.000** | **-0.26** | **-0.12** |
| Recent loneliness in social interaction (0-10) | **-0.13** | **0.05** | **-0.20** | **0.007** | **-0.23** | **-0.04** |
| Current loneliness (0-10) | **0.20** | **0.04** | **0.36** | **0.000** | **0.13** | **0.28** |
| Days elapsed since ESM | 0.01 | 0.01 | 0.04 | 0.451 | -0.01 | 0.02 |
| Age (years) | 0.00 | 0.01 | 0.02 | 0.704 | -0.02 | 0.02 |
| Gender (1 = male) | -0.08 | 0.17 | -0.02 | 0.656 | -0.42 | 0.26 |
| Location (1 = Hong Kong) | 0.07 | 0.18 | 0.02 | 0.719 | -0.30 | 0.43 |
| Living alone (1 = yes) | -0.26 | 0.30 | -0.05 | 0.393 | -0.85 | 0.33 |
| Subjective social status (1-10) | -0.08 | 0.05 | -0.08 | 0.119 | -0.18 | 0.02 |
| Marital status (1 = married) | 0.31 | 0.19 | 0.09 | 0.093 | -0.05 | 0.68 |
| Education (1 = some post-secondary) | -0.19 | 0.22 | -0.04 | 0.404 | -0.62 | 0.25 |
| Income (1 = at or above median) | 0.21 | 0.17 | 0.07 | 0.225 | -0.13 | 0.54 |
| Proportion assessments in social interaction | 0.35 | 0.37 | 0.05 | 0.351 | -0.38 | 1.08 |

Table S29

*Model predicting discrepancy between retrospective and mean momentary reports of tiredness during social interaction (in-person interaction)*

| **Variable** | ***b*** | ***SE*** | ***β*** | ***p*** | ***95% CI(l)*** | ***95% CI(u)*** |
| --- | --- | --- | --- | --- | --- | --- |
| Intercept | **-0.53** | **0.09** | **0.00** | **0.000** | **-0.71** | **-0.36** |
| Introversion (1-5) | 0.00 | 0.13 | 0.00 | 0.995 | -0.26 | 0.26 |
| Self-determined solitude (1-5) | 0.21 | 0.15 | 0.08 | 0.157 | -0.08 | 0.51 |
| Not-self-determined solitude (1-5) | 0.25 | 0.15 | 0.10 | 0.104 | -0.05 | 0.54 |
| Independent self-construal (1-6) | -0.08 | 0.17 | -0.03 | 0.637 | -0.41 | 0.25 |
| Interdependent self-construal (1-6) | -0.17 | 0.14 | -0.07 | 0.202 | -0.44 | 0.09 |
| Peak tiredness in social interaction (0-10) | **-0.20** | **0.06** | **-0.22** | **0.001** | **-0.31** | **-0.09** |
| Recent tiredness in social interaction (0-10) | **-0.21** | **0.05** | **-0.28** | **0.000** | **-0.31** | **-0.12** |
| Current tiredness (0-10) | **0.19** | **0.03** | **0.30** | **0.000** | **0.12** | **0.25** |
| Days elapsed since ESM | 0.01 | 0.01 | 0.04 | 0.442 | -0.01 | 0.03 |
| Age (years) | -0.01 | 0.01 | -0.04 | 0.490 | -0.03 | 0.01 |
| Gender (1 = male) | -0.36 | 0.20 | -0.10 | 0.070 | -0.75 | 0.03 |
| Location (1 = Hong Kong) | **-0.69** | **0.21** | **-0.19** | **0.001** | **-1.12** | **-0.27** |
| Living alone (1 = yes) | 0.07 | 0.35 | 0.01 | 0.848 | -0.62 | 0.75 |
| Subjective social status (1-10) | 0.00 | 0.06 | 0.00 | 0.983 | -0.12 | 0.12 |
| Marital status (1 = married) | 0.18 | 0.22 | 0.05 | 0.404 | -0.24 | 0.61 |
| Education (1 = some post-secondary) | -0.23 | 0.26 | -0.05 | 0.379 | -0.74 | 0.28 |
| Income (1 = at or above median) | 0.25 | 0.20 | 0.07 | 0.212 | -0.14 | 0.64 |
| Proportion assessments in social interaction | 0.11 | 0.44 | 0.01 | 0.806 | -0.76 | 0.97 |
